# Supplementary material for: Design and development of lipid nanoparticle formulations for brain gene therapy
Source: Mol Ther Nucleic Acids. 2026 Jul 20;37(3):103025. doi: 10.1016/j.omtn.2026.103025 (PMC13469846; doi:10.1016/j.omtn.2026.103025)
Supplement: Supplementary file 1 [file mmc1.pdf]

OMTN, Volume *37*

Supplemental information

Design and development of lipid nanoparticle formulations for brain gene therapy

Sarah B. Thomson, Alissandra de Moura Gomes, Pardis Kazemian, Daniel Z. Kurek, Jayesh A. Kulkarni, Fariba Sadaati, Pamela K. Wagner, Terri L. Petkau, Marco A. Ciufolini, Pieter R. Cullis, and Blair R. Leavitt

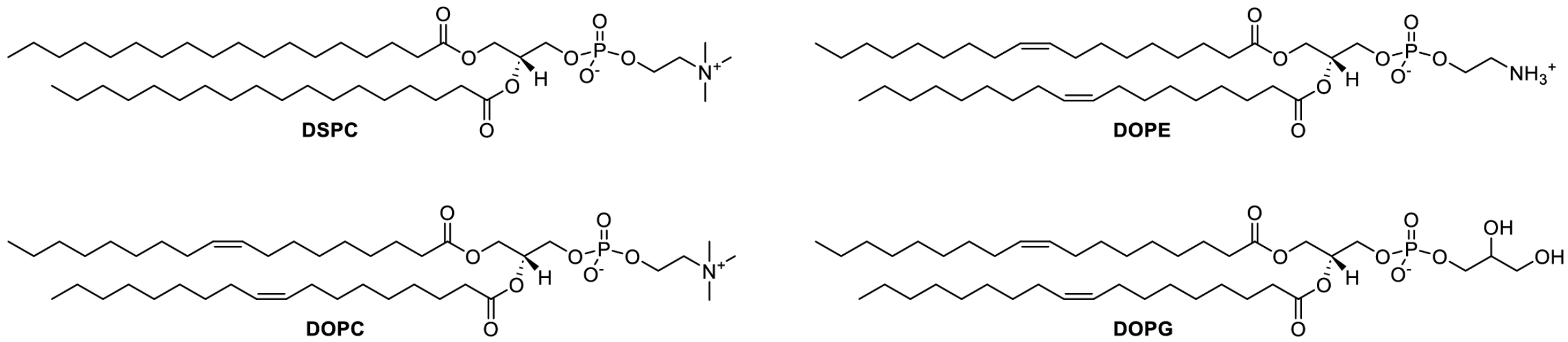

Figure S1. Structures of the phospholipids used for LNP formulation screening.

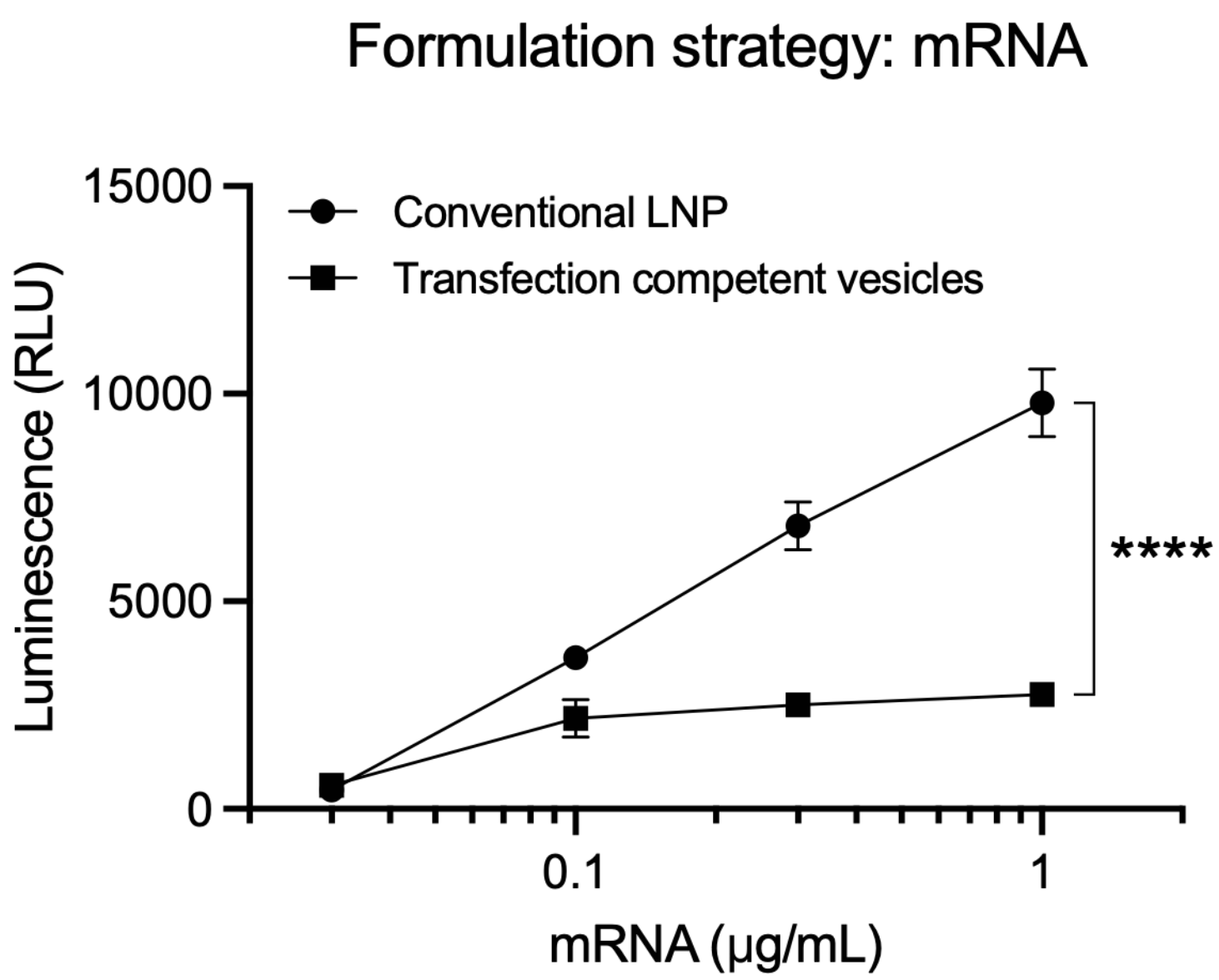

Figure S2. Rapid mixing and benchtop mixing strategies for LNP loading with FLuc mRNA were evaluated. LNPs prepared using rapid mixing had improved activity in primary neurons compared to LNPs loaded using benchtop mixing (2-way ANOVA, Loading method: $F_{(1, 24)} = 472.1$, $p<0.0001$). N=3-4 biological replicates (N=1 technical replicate) per condition. mRNA treatments were performed at 0.03-1.0 μg/mL FLuc mRNA. Data represent the mean (SD).

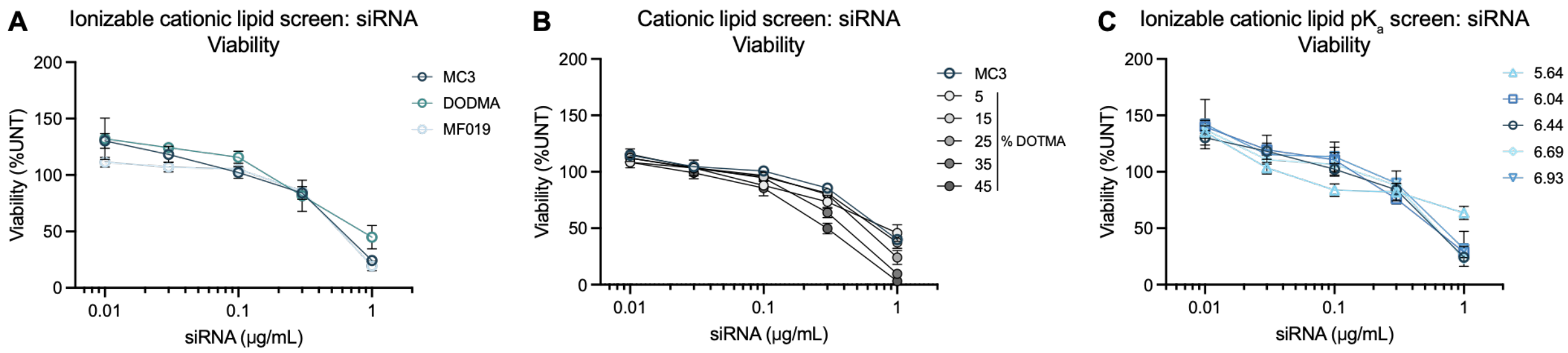

Figure S3. siRNA-containing LNPs prepared with distinct ionizable cationic lipid species differentially affect primary neuron viability. (A) Neuronal viability was impacted by ionizable cationic lipid species and siRNA dose (2-way ANOVA, Composition: $F_{(2,45)}$ = 16.5, p<0.0001; Dose: $F_{(4,45)}$ = 296.2, p<0.0001). (B) Increasing the DOTMA content of MC3 LNPs progressively worsened neuronal viability (2-way ANOVA, Composition: $F_{(4,75)}$ = 45.59, p<0.0001). (C) Ionizable cationic lipid apparent pK_a did not differentially affect neuronal viability (2-way ANOVA, pK_a: $F_{(4,75)}$ = 1.551, p=0.1962). The viability of treated neurons is normalized to the viability measured in untreated primary neurons. N=4 biological replicates (N=1 technical replicate) per condition. siRNA treatments were performed at 0.01-1.0 µg/mL GFP siRNA. Data represent the mean (SD).

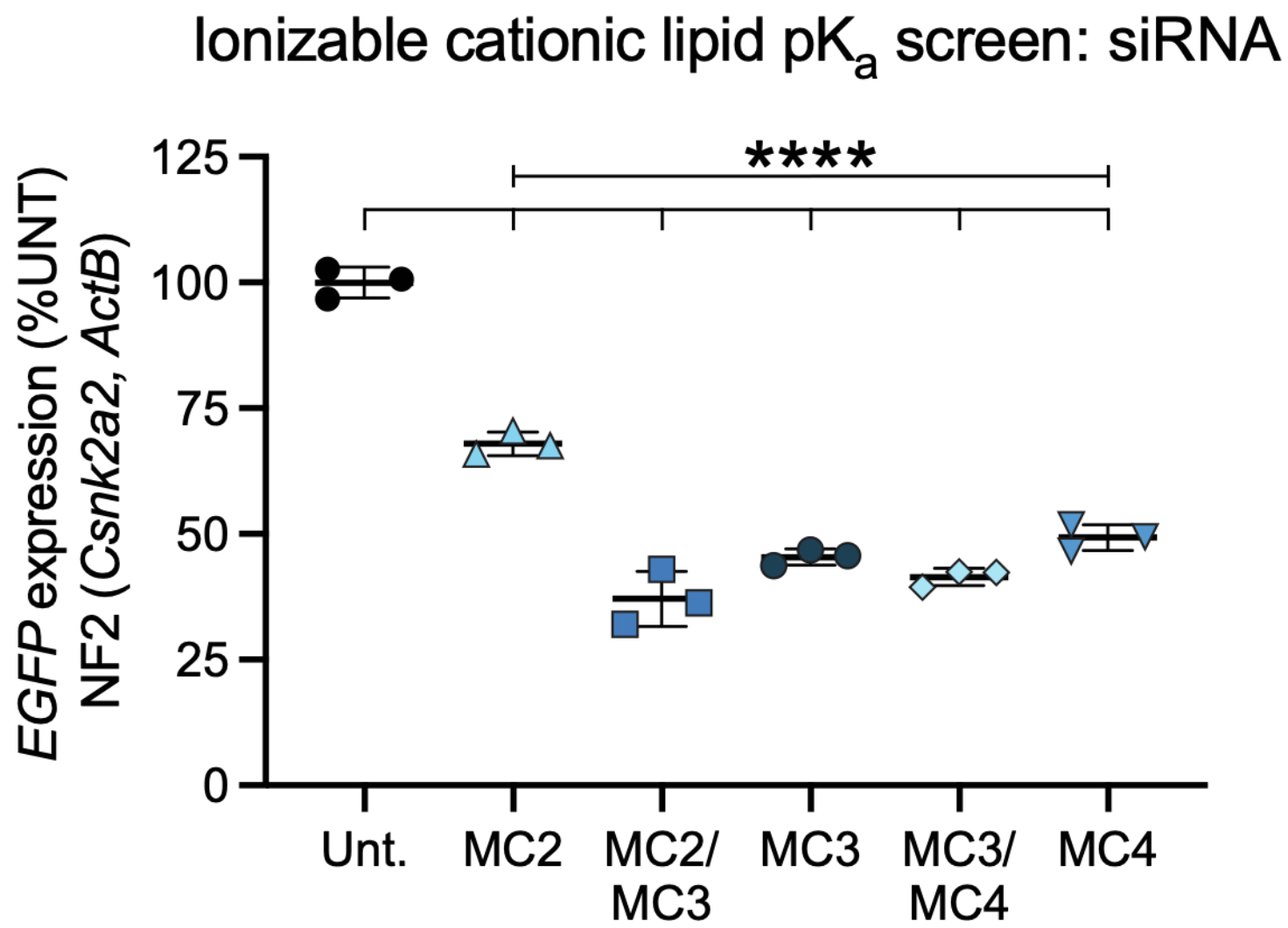

Figure S4. Ionizable cationic lipid apparent pK$_a$ variably impacts the potency of siRNA-containing LNPs in primary neurons. siRNA-containing LNPs prepared using ionizable cationic lipids with a range of apparent pK$_a$s (MC2=5.64; MC2/MC3=6.04; MC3=6.44); MC3/MC4=6.69; MC4=6.93) and containing GFP siRNA knock down *EGFP* in primary neurons with variable efficacy. *EGFP* expression in treated neurons is normalized to the level of *EGFP* expression measured in untreated primary neurons. N=3 biological replicates (N=2 technical replicates) per condition. siRNA treatments were performed at 0.01 µg/mL GFP siRNA. Data represent the mean (SD).

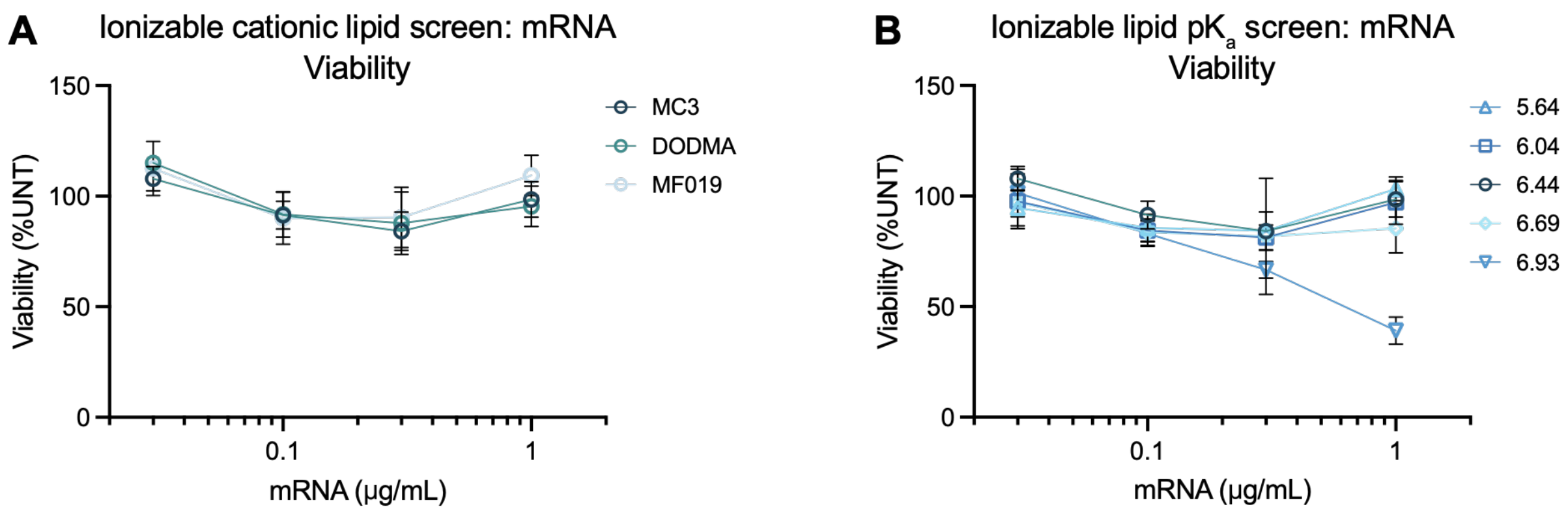

Figure S5. mRNA-containing LNPs prepared with distinct ionizable cationic lipid species differentially affect the viability of primary neurons. (A) Neuronal viability was not impacted by ionizable cationic lipid species (2-way ANOVA, Composition: $F_{(2,36)}$ = 1.108, p=0.3413), but worsened with increased mRNA dose (2-way ANOVA, Dose: $F_{(3,36)}$ = 14.88, p<0.0001). (B) Neuronal viability was affected by both ionizable cationic lipid apparent pK_a (2-way ANOVA, pK_a: $F_{(4,60)}$ = 14.65, p<0.0001) and mRNA dose (2-way ANOVA, pK_a: $F_{(3,60)}$ = 17.39, p<0.0001). N=4 biological replicates (N=1 technical replicate) per condition. mRNA treatments were performed at 0.03-1.0 µg/mL FLuc mRNA. Data represent the mean (SD).

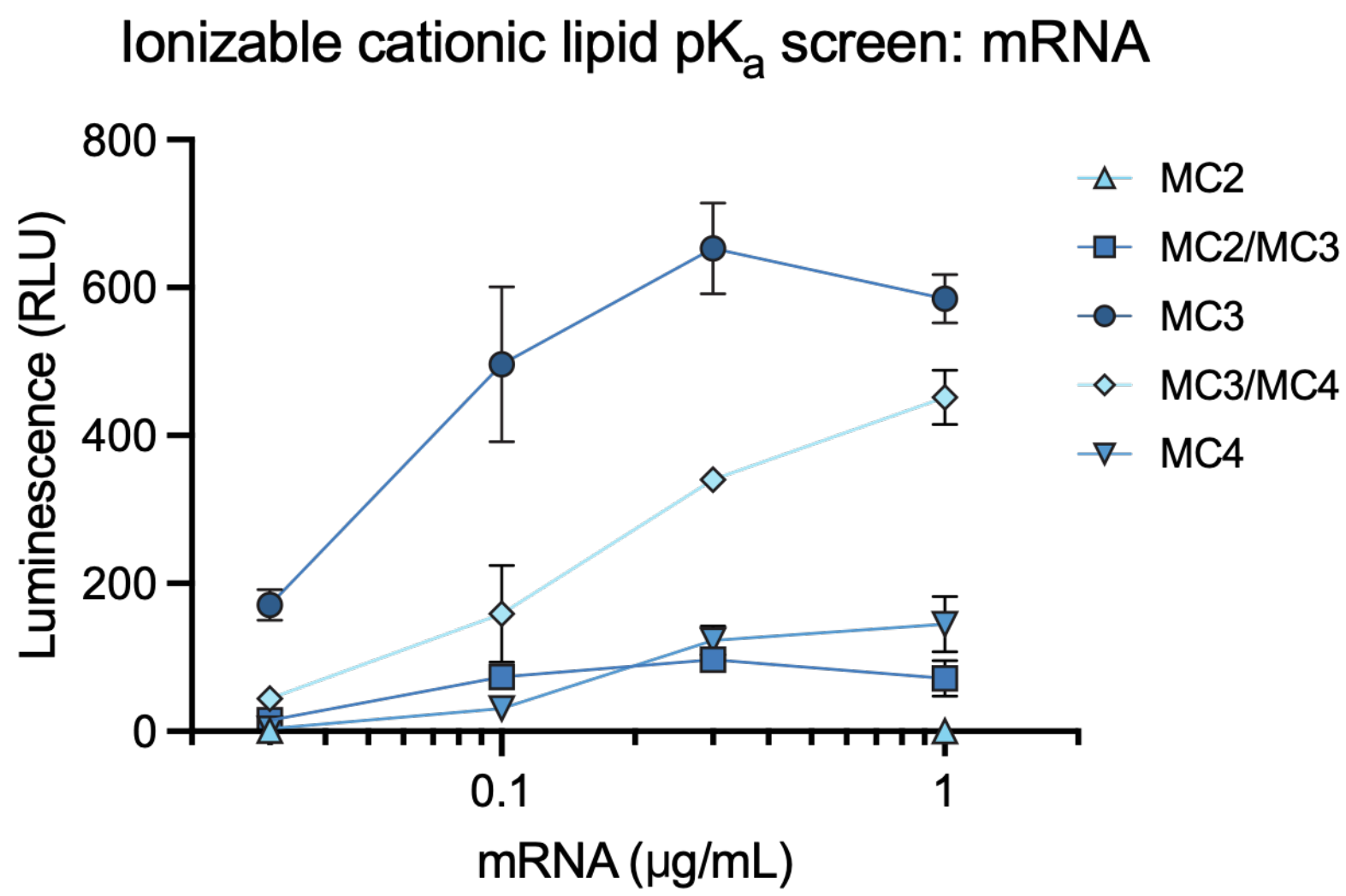

Figure S6. Ionizable cationic lipid apparent pK$_a$ variably impacts the potency of mRNA-containing LNPs in primary neurons. Ionizable cationic lipid apparent pK$_a$ variably impacts the efficacy of LNP-mediated mRNA delivery to primary neurons. LNPs prepared using ionizable cationic lipids with varied apparent pK$_a$s (MC2 = 5.64; MC2/MC3 = 6.03; MC3 = 6.44; MC3/MC4 = 6.69; MC4 = 6.93) and containing FLuc mRNA induced luciferase expression in primary neurons with pK$_a$-dependent efficacy (1-way ANOVA with Dunnett post-test, MC2, MC2/MC3, MC3/MC4, and MC4 vs. MC3 $p<0.0001$). N=4 biological replicates (N=1 technical replicate) per condition. mRNA treatments were performed at 0.03-1.0 µg/mL FLuc mRNA. Data represent the mean (SD).

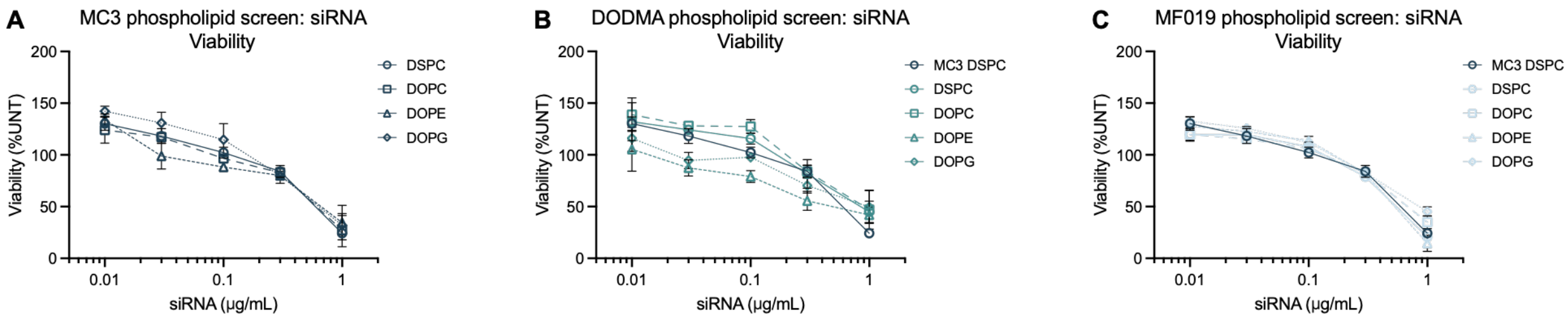

Figure S7. siRNA-containing LNPs prepared with distinct phospholipid species differentially affect primary neuron viability. (A) Neuronal viability was affected by phospholipid species (2-way ANOVA, Phospholipid: $F_{(3,60)}$ = 7.639, p=0.0002) and GFP siRNA dose (2-way ANOVA, Dose: $F_{(4,60)}$ = 311.2, p<0.0001) in the MC3 phospholipid screen. (B) Neuronal viability was affected by phospholipid species (2-way ANOVA, Phospholipid: $F_{(3,60)}$ = 30.48, p<0.0001) and GFP siRNA dose (2-way ANOVA, Dose: $F_{(4,60)}$ = 119.8, p<0.0001) in the DODMA phospholipid screen. (C) Neuronal viability was affected by phospholipid species (2-way ANOVA, Phospholipid: $F_{(3,60)}$ = 22.58, p<0.0001) and GFP siRNA dose (2-way ANOVA, Dose: $F_{(4,60)}$ = 1422, p<0.0001) in the MF019 phospholipid screen. For all viability assays, N=4 biological replicates (N=1 technical replicate) per condition. siRNA treatments were performed at 0.01-1.0 µg/mL GFP siRNA. Data represent the mean (SD).

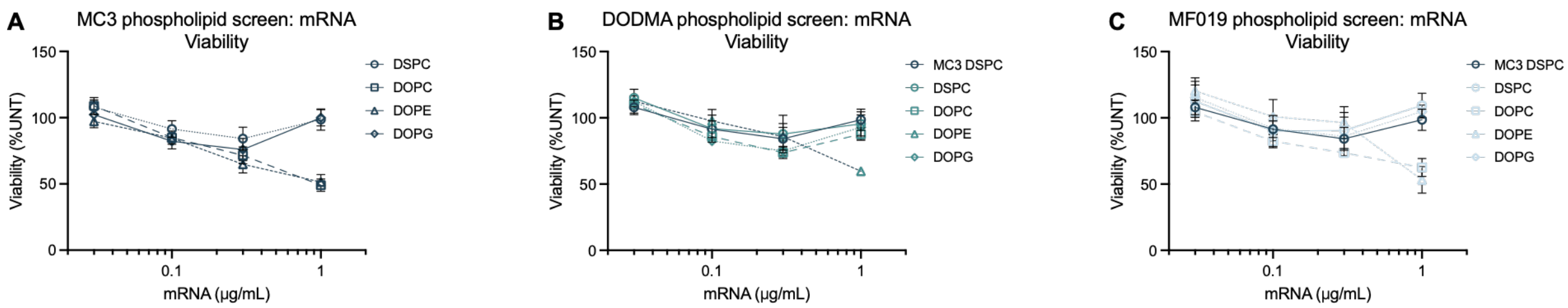

Figure S8. mRNA-containing LNPs prepared with distinct phospholipid species differentially affect primary neuron viability. (A) In the MC3 phospholipid screen, neuronal viability was affected by phospholipid species (2-way ANOVA, Phospholipid: $F_{(3,48)}$ = 36.72, p<0.0001) and mRNA dose (2-way ANOVA, Dose: $F_{(3,48)}$ = 74.97, p<0.0001). (B) In the DODMA phospholipid screen, neuronal viability was affected by phospholipid species (2-way ANOVA, Phospholipid: $F_{(3,48)}$ = 5.042, p=0.0041) and FLuc mRNA dose (2-way ANOVA, Dose: $F_{(3,48)}$ = 64.65, p<0.0001). (C) In the MF019 phospholipid screen, neuronal viability was affected by phospholipid species (2-way ANOVA, Phospholipid: $F_{(3,47)}$ = 12.75, p<0.0001) and FLuc mRNA dose (2-way ANOVA, Dose: $F_{(3,47)}$ = 28.51, p<0.0001). N=4 biological replicates (N=1 technical replicate) per condition. mRNA treatments were performed at 0.03-1.0 µg/mL FLuc mRNA. Data represent the mean (SD).

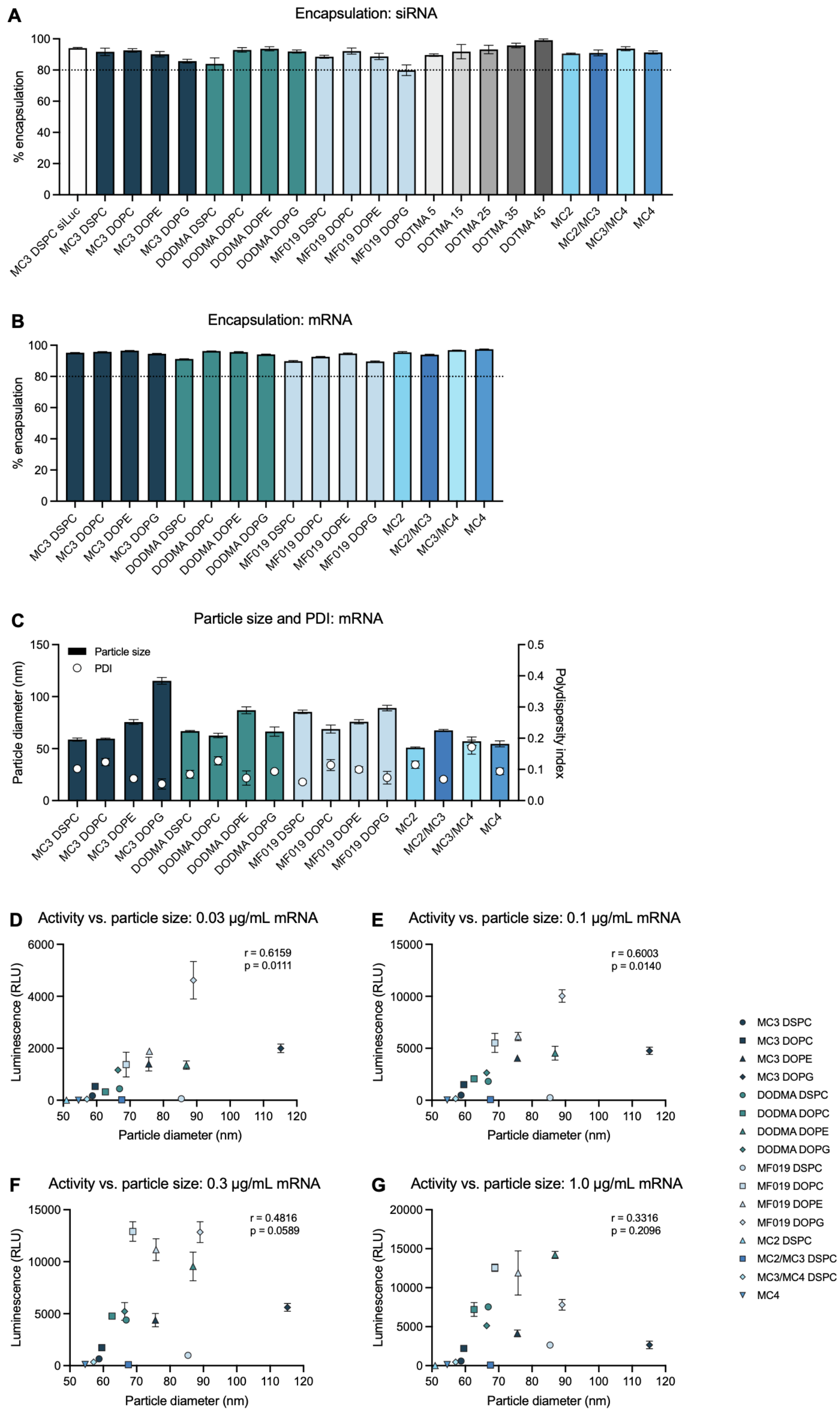

A
Encapsulation: siRNA
% encapsulation
MC3 DSPC siLuc
MC3 DSPC
MC3 DOPC
MC3 DOPE
MC3 DOPG
DODMA DSPC
DODMA DOPC
DODMA DOPE
DODMA DOPG
MF019 DSPC
MF019 DOPC
MF019 DOPE
MF019 DOPG
DOTMA 5
DOTMA 15
DOTMA 25
DOTMA 35
DOTMA 45
MC2
MC2/MC3
MC3/MC4
MC4
B
Encapsulation: mRNA
% encapsulation
C
Particle size and PDI: mRNA
Particle diameter (nm)
Polydispersity index
Particle size
PDI
D
Activity vs. particle size: 0.03 µg/mL mRNA
r = 0.6159
p = 0.0111
E
Activity vs. particle size: 0.1 µg/mL mRNA
r = 0.6003
p = 0.0140
F
Activity vs. particle size: 0.3 µg/mL mRNA
r = 0.4816
p = 0.0589
G
Activity vs. particle size: 1.0 µg/mL mRNA
r = 0.3316
p = 0.2096
Luminescence (RLU)
Particle diameter (nm)
MC3 DSPC
MC3 DOPC
MC3 DOPE
MC3 DOPG
DODMA DSPC
DODMA DOPC
DODMA DOPE
DODMA DOPG
MF019 DSPC
MF019 DOPC
MF019 DOPE
MF019 DOPG
MC2 DSPC
MC2/MC3 DSPC
MC3/MC4 DSPC
MC4

Figure S9. LNP biophysical characteristics are not associated with the activity of GFP siRNA- or FLuc mRNA-containing formulations in *ex vivo* primary neurons. (A) RNA entrapment for all GFP siRNA formulations and (B) FLuc mRNA formulations screened in primary neurons was highly efficient (≥80%) for all formulations. (C) Number-weighted particle diameters and polydispersity indices for all FLuc mRNA formulations. (D-G) In FLuc LNP-treated primary neurons, the relationship between LNP activity and particle diameter depends on FLuc mRNA dose. Particle diameter is positively correlated with activity at 0.03 and 0.1 µg/mL mRNA (Pearson correlation: 0.03 µg/mL r=0.6159 and p=0.0111; 0.1 µg/mL r=0.6003 and p=0.0140), while no correlation was observed at 0.3 µg/mL and 1.0 µg/mL mRNA (Pearson correlation: 0.3 µg/mL r=0.4816 and p=0.0589; 1.0 µg/mL r=0.3316 and p=0.2096). N=3 technical replicates for all FLuc entrapment, particle diameter and PDI measurements. N=4 biological replicates (N=1 technical replicate) for all luminescence measurements. mRNA treatments were performed at 0.03-1.0 µg/mL FLuc mRNA. Data represent the mean (SD).

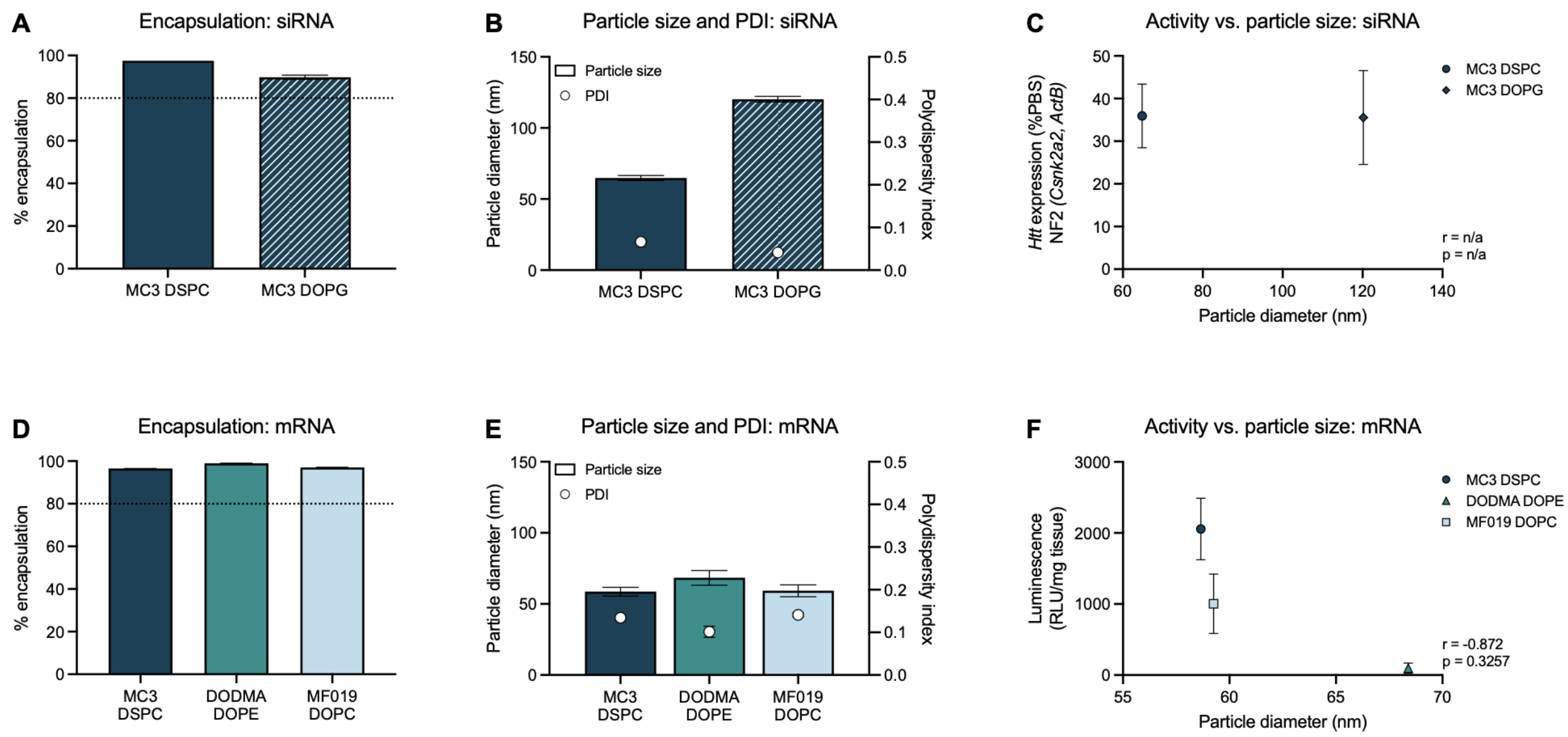

Figure S10. Biophysical characteristics of optimized and benchmark Htt siRNA and FLuc mRNA LNPs screened *in vivo* in striatum. (A) Htt siRNA entrapment was highly efficient (≥80%). (B) Number-weighted particle diameters and polydispersity indices for Htt siRNA formulations. (C) Marked differences in particle diameter between MC3 DSPC and MC3 DOPG LNPs did not impact *in vivo* potency in striatum. 1250 ng of Htt siRNA was delivered per 0.5 µL injection. Brains were collected for analysis seven days post-treatment. (D) FLuc mRNA entrapment was highly efficient (≥80%). (E) Number-weighted particle diameters and polydispersity indices for FLuc mRNA formulations. (F) FLuc mRNA particle diameter is not correlated with *in vivo* potency in striatum (Pearson correlation, r=-0.872 and p=0.3257). 500 ng of FLuc mRNA was delivered per 0.5 µL injection. Brains were collected for analysis four hours post-treatment. N=3 technical replicates for all entrapment, particle diameter and PDI measurements. For activity, N=3-4 biological replicates (for Htt siRNA, N=2 technical replicates; for FLuc mRNA, N=1 technical replicate) per condition. Data represent the mean (SD).

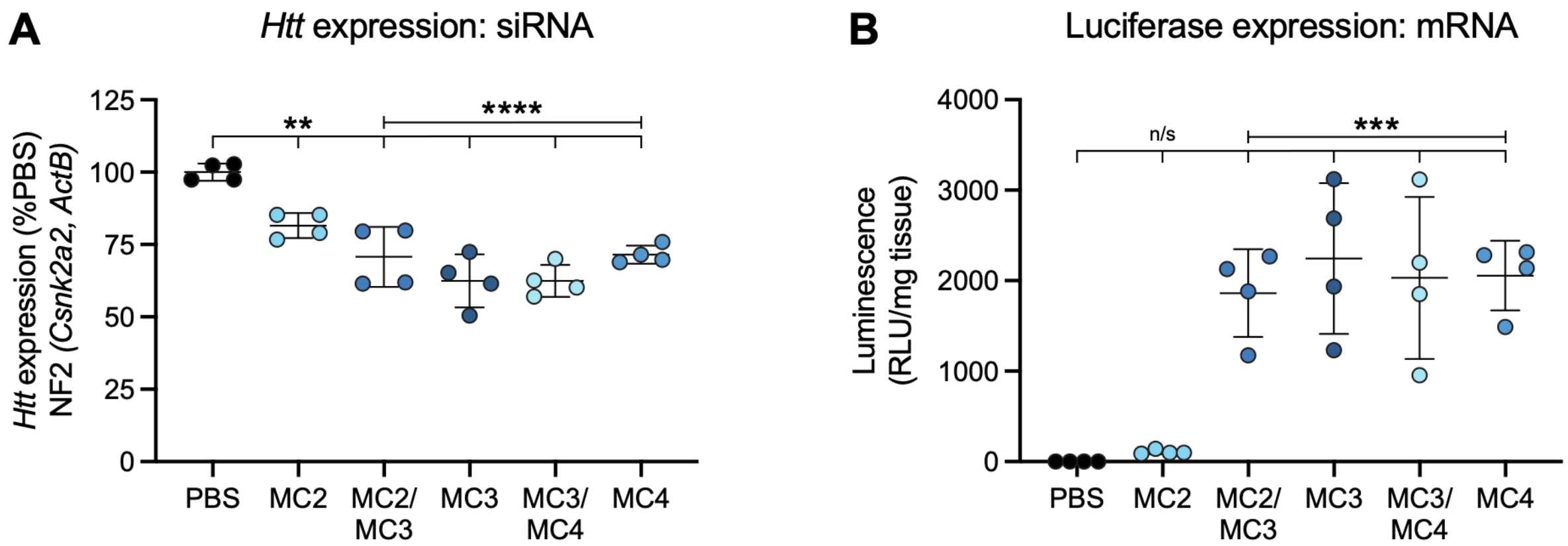

Figure S11. pK$_a$-variant siRNA- and mRNA-containing LNPs are differentially potent *in vivo* in striatum compared to a PBS-injected control group. LNPs were prepared using ionizable cationic lipids with varied apparent pK$_a$s (MC2 = 5.64; MC2/MC3 = 6.03; MC3 = 6.44; MC3/MC4 = 6.69; MC4 = 6.93. (A) Compared to the PBS-injected control group, pK$_a$-variant LNPs containing Htt siRNA knocked down striatal *Htt* with variable efficacy (1-way ANOVA with Dunnett post-test, PBS vs. MC2 p=0.0039; PBS vs. MC2/MC3, MC3, MC3/MC4, and MC4 p<0.0001). *Htt* expression in the striata of treated mice is normalized to the level of *Htt* expression measured in the striata of PBS-injected control mice. 1250 ng of Htt siRNA was delivered per 0.5 µL injection. Brains were collected for analysis seven days post-treatment. (B) Compared to the PBS-injected control group, FLuc mRNA-containing LNPs prepared with MC2/MC3, MC3, MC3/MC4, and MC4 induced striatal luciferase expression (1-way ANOVA with Dunnett post-test, PBS vs. MC2/MC3 p=0.0008; PBS vs. MC3 p=0.0001; PBS vs. MC3/MC3 p=0.0003; PBS vs. MC4 p=0.0003), while the MC2 formulation did not (1-way ANOVA with Dunnett post-test, PBS vs. MC2 p=0.9989). Luminescence in the striata of treated mice is shown relative to the background signal measured in the striata of PBS-injected control mice and normalized to RLU/mg tissue. 500 ng of FLuc mRNA was delivered per 0.5 µL injection. Brains were collected for analysis at four hours post-treatment. For quantitative RT-PCR, N=4 biological replicates (N=2

technical replicates) per condition. For luciferase assays, N=4 biological replicates (N=1 technical replicate) per condition. Data represent the mean (SD).

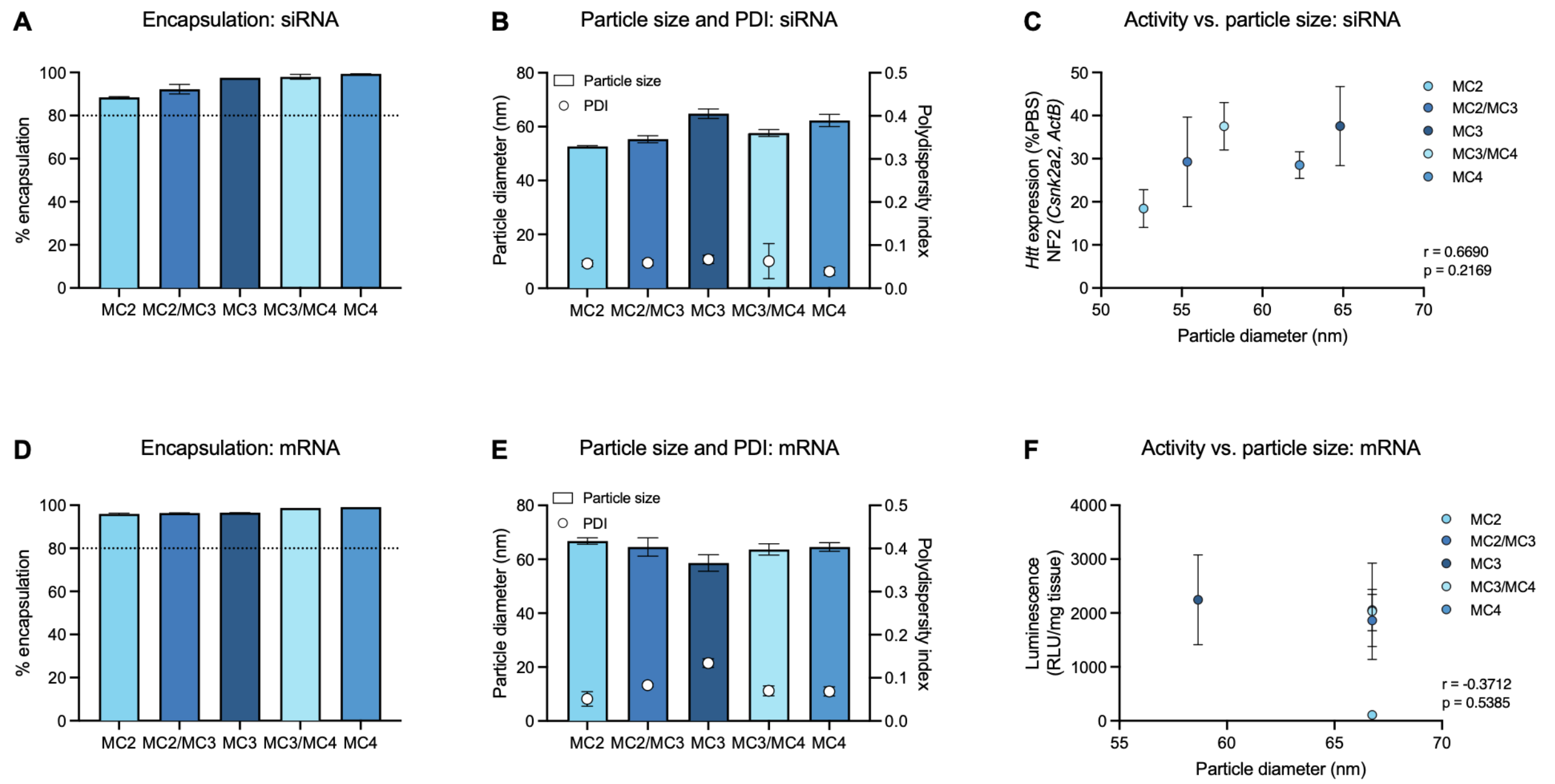

Figure S12. Biophysical characteristics of pK_a variant siRNA and mRNA LNPs screened in striatum *in vivo*. (A) pK_a variant Htt siRNA entrapment was highly efficient (≥80%). (B) Number-weighted particle diameters and polydispersity indices for pK_a variant Htt siRNA formulations. (C) pK_a variant Htt siRNA particle diameter is not correlated with *in vivo* potency in striatum (Pearson correlation, r=0.6690 and p=0.2169). *Htt* expression in the striata of treated mice is normalized to the level of *Htt* expression measured in the striata of PBS-injected control mice. 1250 ng of Htt siRNA was delivered per 0.5 µL injection. Brains were collected for analysis seven days post-treatment. (D) pK_a-variant FLuc mRNA entrapment was highly efficient (≥80%). (E) Number-weighted particle diameters and polydispersity indices for pK_a variant FLuc mRNA formulations. (F) pK_a variant FLuc mRNA particle diameter is not correlated with *in vivo* potency in striatum (Pearson correlation, r=-0.3712 and p=0.5385). Luminescence in the striata of treated mice is shown relative to the background signal measured in the striata of PBS-injected control mice and normalized to RLU/mg tissue. 500 ng of FLuc mRNA was delivered per 0.5 µL injection. Brains were collected for analysis at four hours post-treatment. N=3 technical replicates for all

entrapment, particle diameter and PDI measurements. For activity, N=4 biological replicates (for Htt siRNA, N=2 technical replicates; for FLuc mRNA, N=1 technical replicate) per condition. Data represent the mean (SD).

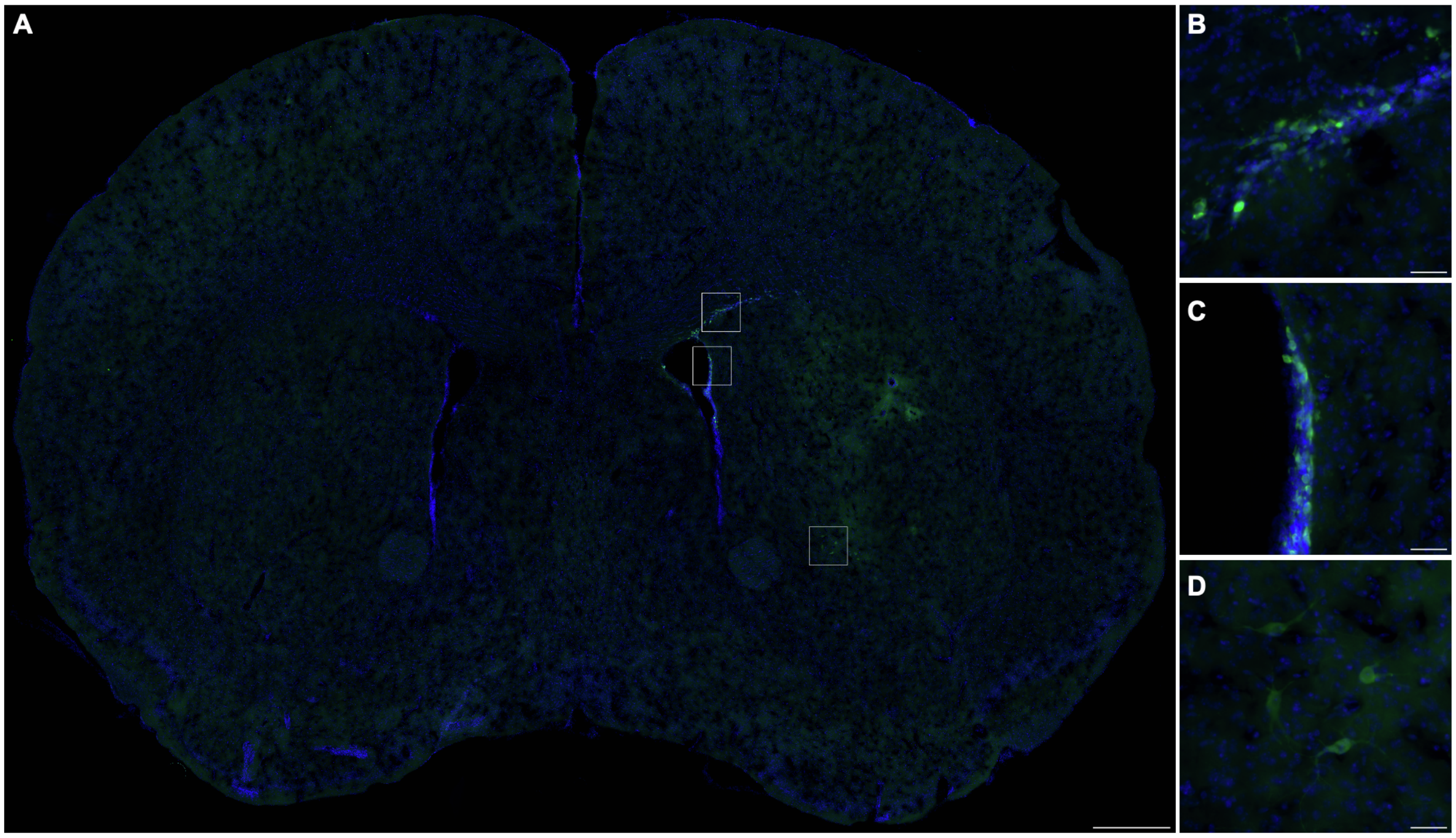

Figure S13. Direct intrastriatal LNP delivery results in luciferase expression in cells throughout and proximal to the striatum. (A) Luciferase expression is detectable in the brain following direct injection of MC3 DSPC LNPs containing FLuc mRNA. Luciferase-expressing cells are present in the corpus callosum (B), adjacent to the lateral ventricle (C) and throughout the striatum (D). Cell nuclei are shown in blue and luciferase-expressing cells are shown in green. 500 ng of FLuc mRNA was delivered per 0.5 µL injection. Brains were collected for analysis four hours post-treatment. The insets outlined in A are presented in the same vertical order in panels B-D. In A, scale bar = 500 µm; in B-D, scale bar = 50 µm.

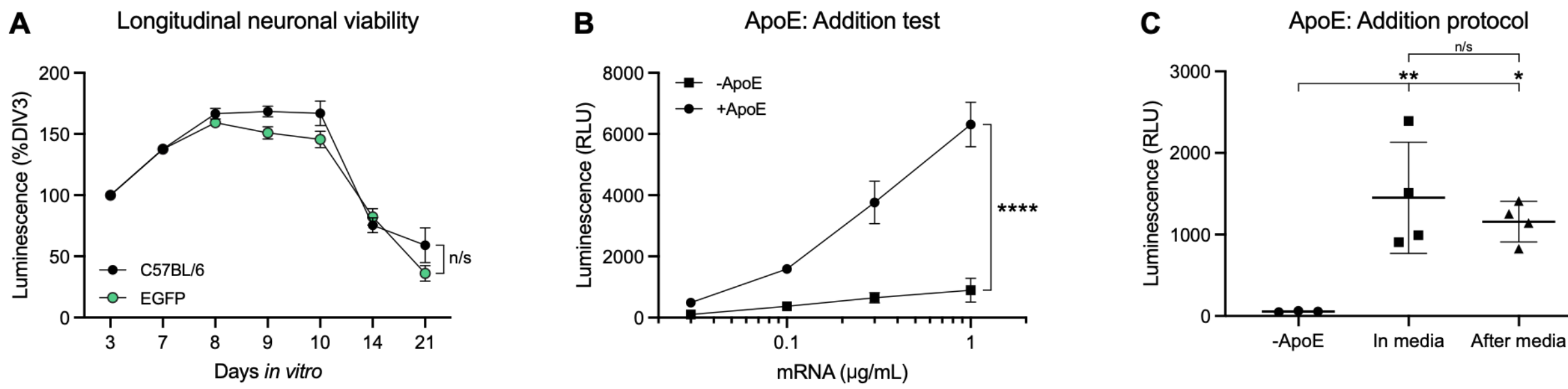

Figure S14. Assay development for LNP treatment of *ex vivo* primary neurons. (A) The viability of primary neurons isolated from C57BL/6 and transgenic *EGFP* E17.5 embryos was measured over 21 days *in vitro* (DIV) to identify appropriate time points for LNP treatment and sample collection. Time significantly impacted neuronal viability (2-way ANOVA, Days *in vitro*: $F_{(2.711, 27.11)} = 677.4$, p<0.0001), while genotype did not (2-way ANOVA, Genotype: $F_{(10, 60)} = 1.962$, p=0.0539). Data are presented as %DIV3. N=6 biological replicates for all measurements. (B) Luciferase expression in C57BL/6 neurons was assayed following treatment with LNPs containing FLuc mRNA in the absence or presence of ApoE to assess the impact of ApoE on neuronal LNP transfection (2-way ANOVA, ApoE: $F_{(1,19)} = 29.37$, p<0.0001). N=3 biological replicates for all measurements. (C) ApoE was added to primary neuron media before or immediately following LNP dilution to establish an ApoE addition protocol appropriate for neuronal LNP transfection. Primary neurons were treated with LNPs containing FLuc mRNA at 0.1 μg/mL. -ApoE = ApoE not added to media; In media = ApoE added to media before LNP dilution; After media = ApoE added to media following LNP dilution (1-way ANOVA with Tukey post-test, -ApoE vs. In media p=0.0084; -ApoE vs. After media p=0.0284; In media vs. After media p=0.6363). N=3-4 biological replicates for all measurements. Data represent the mean (SD).

Table S1. LNP activity is not significantly correlated with ionizable cationic lipid apparent pK_a. In *ex vivo* primary neurons and *in vivo* in the brain, LNP activity and apparent pK_a are not significantly correlated. Relationships between LNP activity and apparent pK_a were assessed by Pearson correlation for each RNA cargo and dose evaluated in the pK_a variant LNP formulation series (see also Figures 2C, 3B, S4, S6 and S12).

Experimental system (RNA)	Comparison	R	95% confidence interval	p
Ex vivo (*GFP* siRNA)	*GFP* knockdown vs. apparent pK_a	-0.5058	-0.9601 to 0.6779	0.3816
Ex vivo (Luc mRNA)	Luminescence vs. apparent pK_a (0.03 µg/mL mRNA)	0.1884	-0.8322 to 0.9181	0.7616
Ex vivo (Luc mRNA)	Luminescence vs. apparent pK_a (0.1 µg/mL mRNA)	0.2310	-0.8180 to 0.9248	0.7085
Ex vivo (Luc mRNA)	Luminescence vs. apparent pK_a (0.3 µg/mL mRNA)	0.4077	-0.7412 to 0.9487	0.4956
Ex vivo (Luc mRNA)	Luminescence vs. apparent pK_a (1.0 µg/mL mRNA)	0.5158	-0.6725 to 0.9608	0.3737
In vivo (*Htt* siRNA)	*Htt* knockdown vs. apparent pK_a	-0.6632	-0.9750 to 0.5281	0.2224
In vivo (Luc mRNA)	Luminescence vs. apparent pK_a	0.8006	-0.2781 to 0.9862	0.1036

Table S2. Quantitative RT-PCR primer sequences.

Target	Forward (5' to 3')	Reverse (5' to 3')
Csnk2a2	CCACATAGACCTAGATCCACACTTCA	AGGTGCCTGTTCTCACTATGGATAA
ActB	CCAGCCTTCCTTCTTGGGTAT	TGTGTTGGCATAGAGGTCTTTACG
EGFP	CAGAAGAACGGCATCAAGG	ACGAACTCCAGCAGGACCAT
Htt	TGGTAATGACAGTTGAGGCCC	CCAGGTTGTACTGCAATGGCT
