# Supplementary material for: Design and development of lipid nanoparticle formulations for brain gene therapy
Source: Mol Ther Nucleic Acids. 2026 Jul 20;37(3):103025. doi: 10.1016/j.omtn.2026.103025 (PMC13469846; doi:10.1016/j.omtn.2026.103025)
Supplement: Supplementary file 2 [file mmc2.pdf]

Molecular Therapy
Nucleic Acids
Original Article

Design and development of lipid nanoparticle formulations for brain gene therapy

Sarah B. Thomson,[1] Alissandra de Moura Gomes,[1] Pardis Kazemian,[1] Daniel Z. Kurek,[2] Jayesh A. Kulkarni,[2,3] Fariba Sadaati,[2,5] Pamela K. Wagner,[1,6] Terri L. Petkau,[1,4] Marco A. Ciufolini,[2,5] Pieter R. Cullis,[3] and Blair R. Leavitt[1,6]

[1]University of British Columbia, Department of Medical Genetics, C201-4500 Oak Street, Vancouver, BC V6H 3N1, Canada; [2]NanoVation Therapeutics, 2665 East Mall, 2nd Floor, Vancouver, BC V6T 1Z4, Canada; [3]University of British Columbia, Department of Biochemistry and Molecular Biology, 2350 Health Sciences Mall, Vancouver, BC V6T 1Z3, Canada; [4]Polymorphic BioSciences, Inc., 2665 East Mall, 2nd Floor, Vancouver, BC V6T 1Z4, Canada; [5]University of British Columbia, Department of Chemistry, 2036 Main Mall, Vancouver, BC V6T 1Z1, Canada; [6]Incisive Genetics, Inc., 200 – 980 George Street, Vancouver, BC V6A 0H9, Canada

Many genetic neurological diseases are caused by toxic gain-of-function of a mutant protein or loss-of-function of a wild-type protein. Treatment of these disorders may be feasible using gene therapy, which requires delivering therapeutic agents to affected brain regions and cells. Lipid nanoparticles (LNPs) have significant potential for this purpose: the clinical safety and efficacy of LNP systems is well-established, and neurons are amenable to LNP-mediated transfection. To adapt LNP technology for brain gene therapy applications, we iteratively designed LNPs to deliver nucleic acids to *ex vivo* primary neurons, and evaluated optimized formulations *in vivo* in the brain. Our approach improved *ex vivo* LNP potency over 1.7-fold for siRNA-containing systems and over 22-fold for mRNA-containing systems, and identified distinct compositions optimal for siRNA and mRNA delivery. We show that the activity of *ex vivo*-optimized LNPs is not always correlated with *in vivo* activity in murine striatum and establish the apparent pK$_a$ of ionizable cationic lipids as an important contributor to LNP efficacy in both model systems. Collectively, we demonstrate robust delivery of multiple macromolecular payloads to primary neurons *ex vivo* and to the brain *in vivo* and validate the utility of LNP systems for gene knockdown and protein replacement brain gene therapies.

INTRODUCTION

Although the prevalence of neurodegenerative disorders is rapidly increasing as the global population ages,[1] there is a notable paucity of effective, disease-modifying therapies available to treat them. Neurological disease etiologies are highly complex and include lifestyle and environmental factors; however, many genes harboring causative or modifier variants also contribute to pathogenesis, such as the CAG trinucleotide repeat expansion in *HTT* that causes Huntington's disease[2] and mutations in *SNCA* and *APOE* associated with Parkinson's and Alzheimer's diseases.[3,4] These genes may be useful therapeutic targets within a treatment paradigm in which gene knockdown addresses toxic gain-of-function disorders and protein replacement addresses wild-type loss-of-function disorders. Such regulatory corrections may be induced using therapeutic nucleic acids, provided they are effectively delivered to relevant cells and tissues in the brain. This approach is uniquely challenging in the context of the numerous neurological diseases that affect neurons, which are quiescent, post-mitotic, and particularly vulnerable to toxicity.[5] As a result, effective and translatable delivery systems that preserve neuronal health are urgently needed to enable the development of novel brain gene therapies.

Lipid nanoparticles (LNPs) are emerging as a promising modality for neuronal drug delivery. In circulation, the adsorption of apolipoprotein E (ApoE) to LNPs facilitates low-density lipoprotein (LDL) receptor-mediated uptake by hepatocytes.[6,7] While most applications of LNP technology focus on delivery to peripheral tissues, LNPs are also functional in the central nervous system due to the presence of the same physiological factors: ApoE is abundant in the brain, and LDL family receptors are highly expressed by neurons.[8–10] LNPs have enabled siRNA-mediated gene silencing *ex vivo* in rat primary neuron cultures and been used to deliver mRNA to mouse and rat neuronal cells *in vivo*[10,11]; thus, LNP functionality in the brain is established. Although the blood-brain barrier significantly limits delivery of systemically administered drugs to the brain, direct intraparenchymal infusion is an acceptable method of drug delivery used in clinical practice that effectively circumvents this limitation.[12] The rapid approval of multiple LNP mRNA COVID-19 vaccines also demonstrates their clinical suitability.[13,14]

Despite the clear utility of LNPs for brain drug delivery, little effort has been made to design bespoke LNP formulations for brain delivery. It is well-established that LNP lipid components drastically impact LNP potency and tolerability,[15,16] and we hypothesized that LNP composition could be tuned to enable efficient and non-toxic delivery of therapeutic nucleic acids to neurons. To optimize LNP formulation parameters for neuronal delivery and to characterize

Received 19 August 2025; accepted 17 July 2026;
https://doi.org/10.1016/j.omtn.2026.103025.

Correspondence: Blair R. Leavitt, 2020-950 West 28th Avenue, Vancouver, BC V5Z 4H4, Canada.
E-mail: bleavitt@cmmt.ubc.ca

© 2026 The Author(s). Published by Elsevier Inc. on behalf of The American Society of Gene and Cell Therapy.
This is an open access article under the CC BY license (http://creativecommons.org/licenses/by/4.0/).

relationships between lipid composition and LNP activity and safety in the context of brain gene therapy, we evaluated the activity and toxicity of 37 distinct LNP formulations of siRNA and mRNA using murine primary neurons *ex vivo*. A number of CNS-focused studies have recently reported consistent LNP activity trends between *ex vivo* and *in vivo* systems, suggesting that evaluating LNP potency in *ex vivo* primary cells may accurately model *in vivo* activity.[10,11,17]

Systematic screening of LNPs prepared with distinct combinations of ionizable cationic lipids and phospholipids identified formulations most suitable for delivering siRNA and mRNA to primary neurons. These screens also significantly improved the delivery efficacy of both nucleic acid payloads in the absence of overt neuronal toxicity, demonstrating that our optimization approach markedly increased LNP potency and safety. Subsequent evaluation of *ex vivo*-optimized LNPs in the brain revealed an alternative pattern of activity, and indicated that ionizable cationic lipid apparent pK_a is an important determinant of LNP potency in both primary neurons *ex vivo* and the brain *in vivo*. Collectively, this work establishes LNPs as a highly effective nucleic acid delivery platform for neurons and the central nervous system and supports the continued development and use of LNP systems for brain gene therapy.

RESULTS

We designed an iterative screening strategy to evaluate the impact of LNP composition on nucleic acid delivery to primary cortical neurons *ex vivo* (Figure 1A) and systematically evaluated the activity of LNP formulations containing both siRNA and mRNA prepared with a range of cationic lipids (Figure 1B) and phospholipids (Figure S1) to assess the effect of lipid composition on formulation potency and neuronal viability. To evaluate the specific impact of lipid species on LNP activity, formulations were prepared using a fixed molar ratio of 50:10:39:1 (mol% ionizable cationic lipid or permanent cationic lipid:phospholipid:cholesterol:PEG lipid), and reporter gene systems were used to avoid disrupting endogenous gene expression. Experiments evaluating LNP-mediated gene knockdown used siRNA targeting *EGFP* mRNA and were performed with primary neurons isolated from transgenic *EGFP* reporter mice,[18] and experiments evaluating protein replacement used mRNA encoding firefly luciferase (FLuc) and were performed with primary neurons isolated from wild-type C57BL/6 mice.

LNPs are typically prepared using a rapid-mixing process during which lipids dissolved in organic solvent are rapidly combined with nucleic acid dissolved in low pH aqueous buffer. A subsequent dialysis step removes organic solvent from the mixture, neutralizes formulation pH, and completes particle formation.[19,20] While this formulation approach was previously believed to achieve LNP formation and nucleic acid entrapment simultaneously, we have recently demonstrated that these processes can be decoupled.[21] Empty, transfection competent vesicles (TCVs) at pH 4 can efficiently encapsulate cargoes by pipette mixing on the benchtop,[22,23] and particle formation is completed by addition of a solution maintained at physiological pH (such as buffer or cell culture medium).[21] This method of LNP preparation is particularly useful for high throughput formulation screening applications because it enables a single preformed TCV formulation to be used for delivering multiple nucleic acid sequences, permits nucleic acid loading into LNPs immediately prior to treating cells, and significantly reduces the quantity of nucleic acid required for LNP production (as little as 20 ng of nucleic acid per formulation is required for TCV loading on the benchtop, compared with ∼250 μg for rapid mixing). We previously reported that the activity of rapid-mixed and benchtop-mixed siRNA-containing LNPs is equivalent in primary neurons[21]; thus, benchtop mixing is a suitable method for loading TCVs with siRNA. To assess the suitability of benchtop mixing for loading TCVs with mRNA, we measured luciferase expression in primary neurons treated with LNPs containing FLuc mRNA loaded using conventional rapid mixing or benchtop pipette mixing. Despite our previous siRNA loading results,[21] rapid-mixed mRNA LNPs were significantly more potent than those prepared using benchtop mixing (Figure S2). To ensure reagent conservation was prioritized without compromising baseline formulation efficacy, all siRNA formulation screening experiments were performed using LNPs loaded by benchtop mixing, and all mRNA formulation screening experiments were performed using LNPs prepared by rapid mixing.

Ionizable cationic lipid species substantially impacts the activity of siRNA- and mRNA-containing LNPs in a payload-dependent manner

Ionizable cationic lipids are critical for enabling two key aspects of LNP functionality: nucleic acid encapsulation and intracellular release.[24] To identify effective ionizable cationic lipids for neuronal RNA delivery, we evaluated the effect of lipid species on LNP formulation potency using both siRNA- and mRNA-containing LNPs. Benchmark control formulations were prepared using heptatriaconta-6,9,28,31-tetraen19-yl-4-(dimethylamino)butanoate (MC3), which enables highly efficient LNP siRNA-mediated hepatic gene silencing *in vivo*[15] and is a component of Onpattro, the first approved LNP siRNA drug.[25] Two additional formulations containing 1,2-dioleyloxy-3-dimethylaminopropane (DODMA), one of the first ionizable cationic lipids developed, and 6,8,26,28-tetrathiatritriacontan-17-yl(dimethylamino)butanoate (MF019)[26] were tested to evaluate the impact of distinct ionizable cationic lipid chemistries on neuronal gene silencing. All formulations were prepared using the phospholipid distearoylphosphatidylcholine (DSPC), which is also a component of Onpattro.[25]

Neuronal *EGFP* expression was quantified 72 h post-LNP treatment. *EGFP* expression was equivalent in untreated neurons and neurons treated with MC3 LNPs containing a non-targeting scramble siRNA, demonstrating MC3 LNP treatment alone did not impact neuronal *EGFP* expression (Figure 2A). Among formulations containing GFP siRNA, MC3 LNPs induced 54.6% (1.6%) *EGFP* knockdown, while DODMA and MF019 formulations were less effective and induced 18.5% (5.8%) and 33.3% (6.0%) *EGFP* knockdown (Figure 2A).

Because primary neurons are notably vulnerable to the toxicities of many widely used transfection reagents,[27] we evaluated neuronal

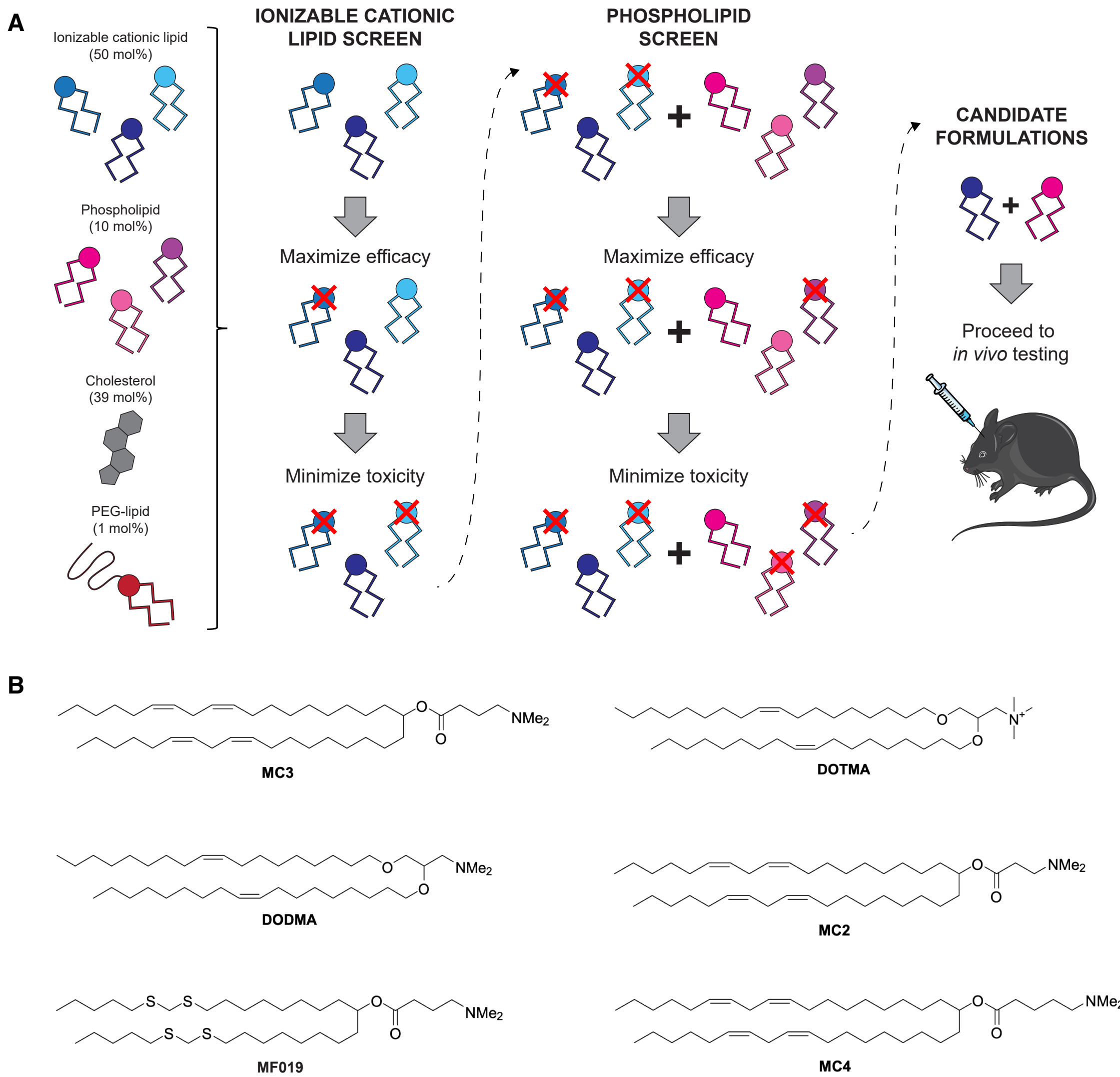

Figure 1. LNP formulation screening was performed in *ex vivo* in primary cortical neurons using an iterative screening strategy, and subsequently evaluated *in vivo* in the brain

(A) The workflow designed to optimize LNP composition for neuronal siRNA and mRNA delivery. (B) Structures of the ionizable cationic lipids and the permanently cationic lipid DOTMA used to prepare LNPs.

viability in parallel to *EGFP* expression. Viability was differentially affected by siRNA dose (Figure S3A); for all compositions, viability was well-preserved compared with untreated neurons at the 0.01 μg/mL siRNA screening dose, remained relatively stable at doses up to two orders of magnitude larger, and decreased at the highest tested siRNA dose (0.3 and 1.0 μg/mL) (Figure S3A). Viability was also impacted by ionizable cationic lipid species, and relationships between formulation composition and viability changed depending on siRNA dose. For example, neurons treated with MC3-containing LNPs at 0.01 μg/mL siRNA were healthier than those treated with MF019-containing LNPs, while similar levels of toxicity were measured between the two formulations at higher doses (Figure S3A). Higher siRNA doses reduced neuronal viability regardless of ionizable cationic lipid species. The degree of *EGFP* knockdown measured at the 0.01 μg/mL siRNA screening dose, however, indicates that more siRNA is not required for effective LNP-mediated neuronal gene knockdown.

Next, we prepared a series of siRNA LNPs using MC3 and increasing molar percentages of the cationic lipid 1,2-di-*O*-octadecenyl-3-trimethylammonium propane (DOTMA) to explore if incorporating a cationic lipid with a permanent positive charge would further improve MC3 formulation potency. The total cationic lipid content of the formulations was fixed at 50 mol%, and equivalent molar quantities of MC3 were replaced with DOTMA to produce a series of formulations containing 5%–45% DOTMA (and 45%–5%

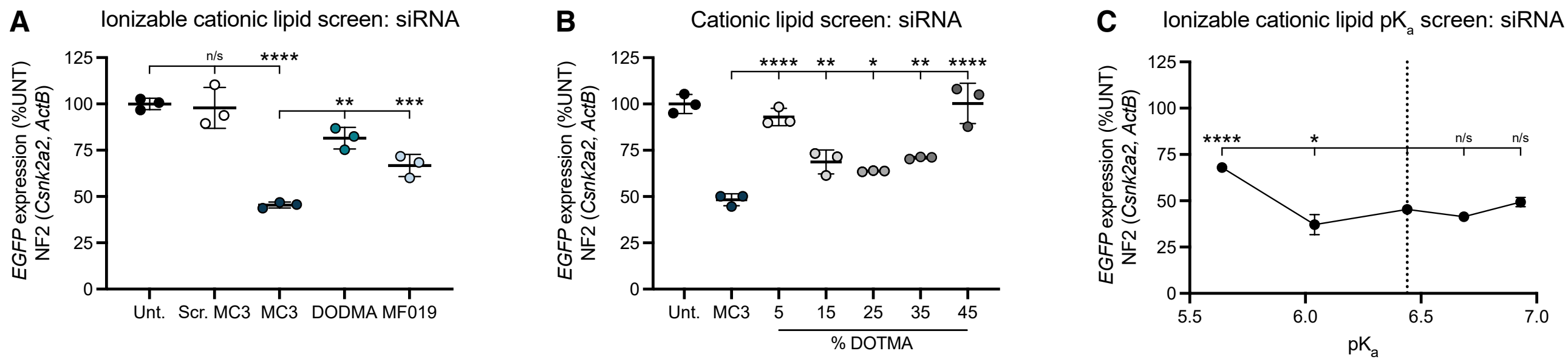

Figure 2. Ionizable cationic lipid species significantly affects the efficacy of LNP siRNA-mediated *EGFP* knockdown in primary neurons

(A) *EGFP* expression was unaffected in neurons treated with a benchmark LNP formulation containing the ionizable cationic lipid MC3, phospholipid DSPC, and non-targeting scramble siRNA (Scr.; unpaired *t* test, $p = 0.7643$). *EGFP* knockdown was achieved by treatment with the same benchmark formulation containing GFP siRNA (unpaired *t* test, $p < 0.0001$). The magnitude of *EGFP* knockdown was less robust following substitution of MC3 with the ionizable cationic lipids DODMA (one-way ANOVA with Dunnett post-test, $p = 0.0002$) and MF019 (one-way ANOVA with Dunnett post-test, $p = 0.0033$). (B) Relative to the benchmark MC3-only formulation, *EGFP* knockdown was worsened by addition of the permanently cationic lipid DOTMA to MC3-containing LNPs (one-way ANOVA with Dunnett post-test, $^{*}p < 0.05$, $^{**}p < 0.01$, $^{****}p < 0.0001$). (C) Ionizable cationic lipid apparent pK_a differentially affected *EGFP* knockdown (one-way ANOVA with Dunnett post-test, $^{*}p = 0.0262$, $^{****}p < 0.0001$). The vertical dotted line at X = 6.44 denotes the apparent pK_a of the MC3 benchmark formulation (see also Figure S4). In all images, *EGFP* expression in treated neurons is normalized to the level of *EGFP* expression measured in untreated primary neurons (abbreviated "Unt."). *N* = 3 biological replicates (*N* = 2 technical replicates) per condition. siRNA treatments were performed at 0.01 μg/mL GFP siRNA. Data represent the mean (SD).

MC3). Partial replacement of MC3 with DOTMA reduced the efficacy of all formulations, and while 29.0% (4.7%)–31.3% (0.6%) *EGFP* knockdown was achieved with formulations containing intermediate (15%–35%) DOTMA percentages, formulations containing 5% and 45% DOTMA failed to knock down *EGFP* (Figure 2B). None of the DOTMA-containing formulations was as effective as the MC3-only control, and DOTMA adversely impacted neuronal viability (Figure S3B), demonstrating that DOTMA is an unsuitable cationic lipid for LNP-mediated siRNA delivery to primary neurons.

The relationship between ionizable cationic lipid chemistry and LNP efficacy has been extensively investigated, and ionizable cationic lipid apparent pK_a has been identified as a significant predictor of *in vivo* LNP potency.[15] To evaluate the impact of ionizable cationic lipid apparent pK_a on siRNA delivery to primary neurons *ex vivo*, we tested a series of formulations containing ionizable cationic lipids with a range of apparent pK_as. LNPs were prepared using heptatriaconta-6,9,28,31-tetraen19-yl-4-(dimethylamino)propanoate (MC2; $pK_a = 5.64$), MC3 ($pK_a = 6.44$), or heptatriaconta-6,9,28,31-tetraen19-yl-4-(dimethylamino)pentanoate (MC4; $pK_a = 6.93$) individually, as well as with equimolar blends of MC2 and MC3 ($pK_a = 6.04$), and MC3 and MC4 ($pK_a = 6.69$), which produced a series of formulations with varied ionizable cationic lipid apparent pK_a values (hereafter referred to as "pK_a variant LNPs"). All pK_a variant LNPs induced significant *EGFP* knockdown (Figures 2C and S4), and the most robust *EGFP* knockdown was achieved using pK_a variant formulations with intermediate apparent pK_as of 6.04 (62.9% [5.5%] *EGFP* knockdown), 6.44 (54.6% [1.6%] *EGFP* knockdown), and 6.69 (58.8% [2.6%] *EGFP* knockdown), suggesting the efficacy of neuronal siRNA delivery may, in part, be governed by this variable. No significant effect of apparent pK_a on viability was measured (Figure S3C), indicating that neuronal health was primarily impacted by treatment dose.

Previous work has shown that differences in size and molecular conformation between siRNA and mRNA mean the two molecule classes often require different LNP compositions for optimal *in vivo* efficacy.[28] To explore this possibility in the context of *ex vivo* primary neuron transfection, we repeated the same series of ionizable cationic lipid screens (minus DOTMA-containing formulations, because DOTMA-containing LNPs failed to improve siRNA delivery and adversely impacted neuronal viability) using LNP formulations of mRNA. Primary neurons were first treated with FLuc mRNA-containing LNPs prepared with MC3, DODMA, or MF019 in combination with the phospholipid DSPC across a range of mRNA doses (0.03–1.0 μg/mL). In contrast with the results of our siRNA screens, DODMA LNPs were significantly more efficacious than the MC3 and MF019 formulations (Figure 3A). The less potent MC3- and MF019-containing formulations performed similarly, with slightly better activity measured in MF019-treated neurons at higher mRNA doses (Figure 3A). The impact of LNP treatment on neuronal viability was minimal (Figure S5A), and varied with dose but not ionizable cationic lipid species.

We also evaluated the effect of ionizable cationic lipid apparent pK_a upon mRNA delivery. In alignment with the results of our siRNA screens, ionizable cationic lipid apparent pK_a significantly impacted mRNA LNP activity (Figures 3B and S6). The most potent pK_a variant formulation had an ionizable cationic lipid apparent pK_a of 6.44, while formulations with lower and higher ionizable cationic lipid apparent pK_a values were less effective. Overall, formulations with higher ionizable cationic lipid apparent pK_a values performed better than those with lower apparent pK_a values, particularly at higher mRNA doses (Figures 3B and S6). While apparent pK_a minimally impacted neuronal health in this screen, the viability of neurons treated with the highest apparent pK_a (6.93) formulation was significantly lower (Figure S5B).

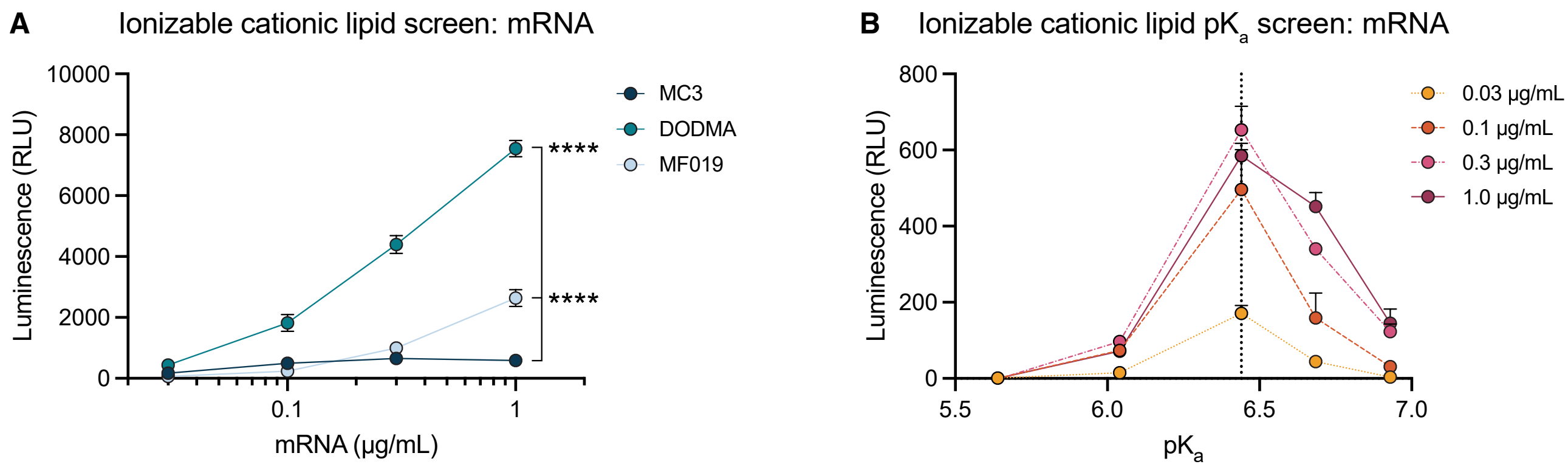

Figure 3. Ionizable cationic lipid species significantly affects the efficiency of LNP mRNA-mediated luciferase expression in primary neurons
(A) Luciferase expression was measurable following treatment with the benchmark LNP formulation containing FLuc mRNA, and MC3 substitution with DODMA and MF019 significantly improved formulation potency (two-way ANOVA with Tukey post-test, $p < 0.0001$). (B) Ionizable cationic lipid apparent pK_a variably impacted formulation potency (two-way ANOVA, pK_a: $F_{(4,60)} = 472.4$, $p < 0.0001$). pK_a variant formulations were prepared using MC2 ($pK_a = 5.64$), MC3 ($pK_a = 6.44$), MC4 ($pK_a = 6.93$) and equimolar blends of MC2 and MC3 ($pK_a = 6.04$), and MC3 and MC4 ($pK_a = 6.69$) (see also Figure S6). The vertical dotted line at X = 6.44 denotes the apparent pK_a of the MC3 benchmark formulation. N = 4 biological replicates (N = 1 technical replicate) per condition. mRNA treatments were performed at 0.03–1.0 µg/mL FLuc mRNA. Data represent the mean (SD).

Phospholipids augment the activity of siRNA- and mRNA-containing LNPs

All formulations evaluated in the ionizable cationic lipid screens were prepared using the phospholipid DSPC, which, like MC3, is present in Onpattro.[25] Because phospholipid species also impact LNP potency,[16] we compared the activity of DSPC-containing formulations to LNPs prepared with three alternative phospholipids with unique chemistries: 1,2-dioleoyl-sn-glycero-3-phosphocholine (DOPC), 1,2-dioleoyl-sn-glycero-3-phosphoethanolamine (DOPE), and 1,2-dioleoyl-sn-glycero-3-phospho-(1′-rac-glycerol) (DOPG). Compared with DSPC-containing LNPs, nearly all siRNA formulations prepared with alternative phospholipids were more potent (Figure 4). In the MC3 series, the 54.6% (1.6%) *EGFP* knockdown achieved using the benchmark formulation was progressively improved by substitution of DSPC with DOPC (75.0% [0.6%] *EGFP* knockdown), DOPE (88.2% [0.1%] *EGFP* knockdown), and DOPG (95.3% [0.2%] *EGFP* knockdown) (Figure 4A). Similar to the results of the ionizable cationic lipid screens, neuronal viability decreased with increasing siRNA dose and was differentially affected by phospholipid species (Figure S7A). Neurons treated with the MC3

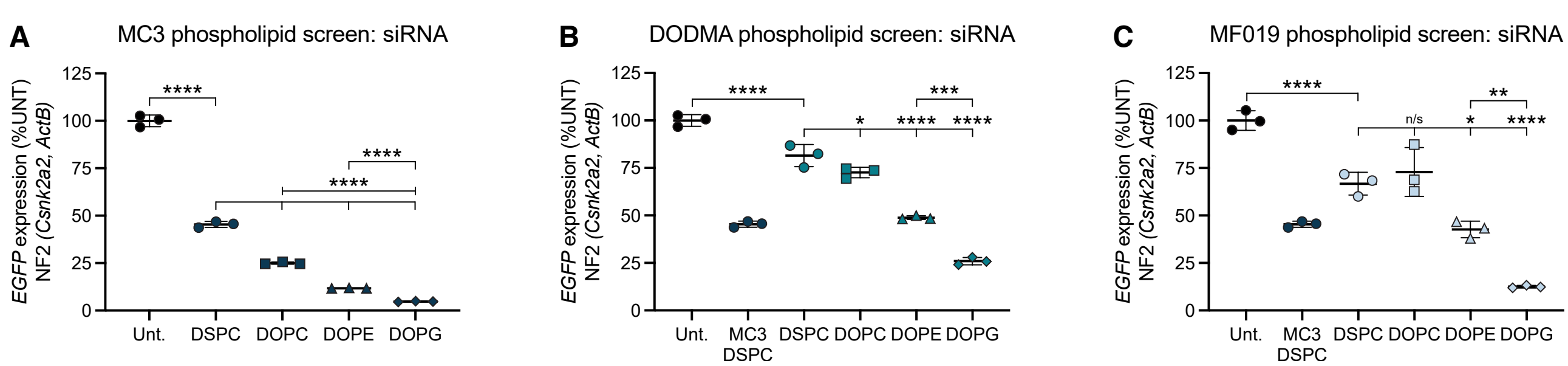

Figure 4. Phospholipid species significantly impacts the efficiency of LNP siRNA-mediated *EGFP* knockdown in primary neurons
(A) In MC3 LNPs, *EGFP* knockdown was improved by substitution of the phospholipid DSPC (unpaired t test, $p < 0.0001$) with DOPC (one-way ANOVA with Dunnett post-test, $p < 0.0001$), DOPE (one-way ANOVA with Dunnett post-test, $p < 0.0001$), and DOPG (one-way ANOVA with Dunnett post-test, $p < 0.0001$). The most effective *EGFP* knockdown was induced by the MC3 DOPG formulation (one-way ANOVA with Tukey post-test, DOPE vs. DOPG, $p < 0.0001$). (B) In DODMA LNPs, *EGFP* knockdown was differentially affected by DSPC (unpaired t test, $p < 0.0001$) substitution with DOPC (one-way ANOVA with Dunnett post-test, $p = 0.0317$), DOPE (one-way ANOVA with Dunnett post-test, $p < 0.0001$), and DOPG (one-way ANOVA with Dunnett post-test, $p < 0.0001$). The most effective *EGFP* knockdown was induced by the DODMA DOPG formulation (one-way ANOVA with Tukey post-test, DOPE vs. DOPG, $p = 0.0002$). (C) In MF019 LNPs, DSPC (unpaired t test, $p < 0.0001$) substitution with DOPC did not improve formulation potency (one-way ANOVA with Dunnett post-test, $p = 0.6324$), while potency improved following substitution of DSPC with DOPE (one-way ANOVA with Dunnett post-test, $p = 0.0103$) and DOPG (one-way ANOVA with Dunnett post-test, $p < 0.0001$). The most effective *EGFP* knockdown was induced by the MF019 DOPG formulation (one-way ANOVA with Tukey post-test, DOPE vs. DOPG, $p = 0.0048$). In all images, *EGFP* expression in treated neurons is normalized to the level of *EGFP* expression measured in untreated primary neurons (abbreviated "Unt."). N = 3 biological replicates (N = 2 technical replicates) per condition. siRNA treatments were performed at 0.01 µg/mL GFP siRNA. Data represent the mean (SD).

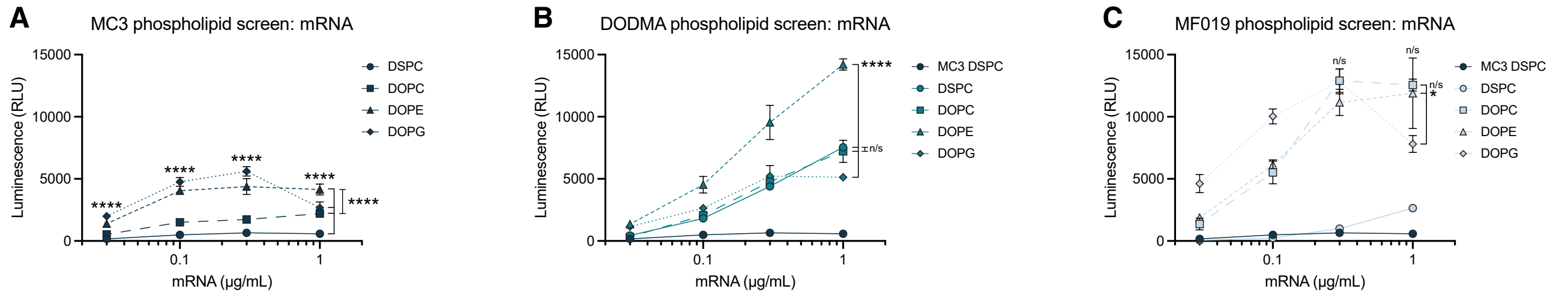

Figure 5. Phospholipid species significantly affects the magnitude of LNP mRNA-mediated luciferase expression in primary neurons

(A) In MC3 LNPs, DSPC substitution with DOPC, DOPE, and DOPG improved LNP activity (two-way ANOVA with Dunnett post-test, $p < 0.0001$). MC3 DOPG LNPs were most effective at the three lowest FLuc mRNA doses tested (two-way ANOVA with Tukey post-test, 0.01–0.3 μg/mL DOPG vs. DOPE, $p < 0.0001$), and the DOPE formulation was most effective at 1.0 μg/mL FLuc mRNA (two-way ANOVA with Tukey post-test, 1.0 μg/mL DOPE vs. DOPG, $p < 0.0001$). (B) In DODMA LNPs, formulation potency was robust and significantly affected by phospholipid species (two-way ANOVA, Phospholipid: $F_{(3,48)} = 211.9$, $p < 0.0001$), and the DODMA DOPE formulation was most effective (two-way ANOVA with Dunnett post-test, DODMA DOPE vs. DODMA DSPC, $p < 0.0001$). (C) In MF019-containing LNPs, phospholipid species also affected formulation potency (Phospholipid: $F_{(3,48)} = 244.2$, $p < 0.0001$). The DOPC- and DOPG-containing formulations were most effective (two-way ANOVA with Tukey post-test, DOPC vs. DOPG, $p = 0.1284$), while the DOPE formulation was less potent (two-way ANOVA with Tukey post-test, DOPC vs. DOPE, $p = 0.0126$). The DOPC- and DOPG-containing formulations performed equivalently at 0.3 μg/mL mRNA (two-way ANOVA with Tukey post-test, DOPC vs. DOPG, $p = 0.9996$), and the DOPC- and DOPE-containing formulations performed equivalently at 1.0 μg/mL mRNA (two-way ANOVA with Tukey post-test, DOPC vs. DOPE, $p = 0.7538$). $N = 4$ biological replicates ($N = 1$ technical replicate) per condition. mRNA treatments were performed at 0.03–1.0 μg/mL FLuc mRNA. Data represent the mean (SD).

DOPG formulation were also healthier than those treated with formulations containing DSPC, DOPC, and DOPE (Figure S7A). The DODMA phospholipid series induced less effective *EGFP* knockdown, but exhibited the same phospholipid-dependent trend as the MC3 series (Figure 4B). Treatment with the DODMA DSPC formulation resulted in 18.5% (5.8%) *EGFP* knockdown, while the most effective formulation contained DOPG and induced 74.1% (1.9%) *EGFP* knockdown (Figure 4B). The MF019 phospholipid series showed intermediate potency: the DSPC formulation induced 33.3% (6.0%) *EGFP* knockdown, replacement of DSPC with DOPC resulted in 27.1% (12.8%) *EGFP* knockdown, and formulations containing DOPE (57.4% [4.4%] *EGFP* knockdown) and DOPG (87.5% [0.7%] *EGFP* knockdown) followed the previously observed trend of increased potency (Figure 4C). Both phospholipid species and siRNA dose impacted neuronal viability following treatment with DODMA LNPs (Figure S7B). The viability of neurons treated with MF019-containing LNPs was affected by phospholipid species and siRNA dose, and as observed with the MC3 formulation series, MF019 DOPG-treated neurons were healthier than those treated with formulations containing DSPC, DOPC, and DOPE (Figure S7C).

Finally, phospholipid screens were repeated using LNP formulations of mRNA (Figure 5). MC3-containing LNPs prepared with DOPG were most potent at the three lowest mRNA doses tested, followed by those containing DOPE, DOPC, and DSPC, while the MC3 DOPG formulation was less effective than the MC3 DOPE formulation at 1.0 μg/mL mRNA (Figure 5A). Neuronal viability gradually decreased as mRNA dose increased (Figure S8A). At 1.0 μg/mL mRNA, viability stabilized in MC3 DSPC- and MC3 DOPG-treated neurons, and continued to decline in MC3 DOPC- and MC3 DOPE-treated neurons, demonstrating a variable effect of phospholipid species on neuronal health (Figure S8A). All DODMA-containing LNPs were significantly more potent than the MC3 DPSC control, and among the DODMA-containing formulations, phospholipid species significantly affected LNP efficacy (Figure 5B). Luminescence increased with mRNA dose for DSPC, DOPC, and DOPE formulations, while luminescence did not increase beyond 0.3 μg/mL FLuc mRNA delivered with DODMA DOPG LNPs (Figure 5B). DODMA DOPE LNPs were the most robust and, without adversely impacting neuronal viability, increased luciferase expression more than 22-fold at 0.3 μg/mL mRNA compared witho the MC3 DSPC control (Figures 5B and S8B). Except for DOPE-containing LNPs, we observed a similar trend in neuronal viability in DODMA LNP-treated neurons, with gradual phospholipid- and dose-dependent decreases in viability plateauing at 1.0 μg/mL mRNA (Figure S8B). The viability of DODMA DOPE-treated neurons decreased further at 1.0 μg/mL mRNA (Figure S8B). In the MF019 phospholipid screen, the DSPC-containing formulation performed similarly to the MC3 DSPC control, and substitution with other phospholipids significantly improved formulation activity (Figure 5C). MF019 formulation efficacy also changed with mRNA dose: at lower doses, DOPG-containing LNPs were more potent than those prepared with DOPC or DOPE and, as observed with other DOPG-containing formulations, the efficacy of MF019 DOPG LNPs dropped sharply at 1.0 μg/mL mRNA (Figure 5C). MF019 DOPC- and MF019 DOPE-containing formulations induced equivalent levels of luciferase expression (Figure 5C). Phospholipid species and mRNA dose also significantly impacted neuronal health in cells treated with MF019-containing LNPs (Figure S8C). The viability of neurons treated with MC3 DSPC LNPs was comparable with that of neurons treated with MF019 DSPC LNPs, and treatment with DSPC and DOPG formulations decreased neuronal viability with increasing mRNA dose, except at 1.0 μg/mL mRNA, where a small increase in viability was measured (Figure S8C). In contrast, MF019 formulations containing DOPC and DOPE caused progressive decreases in viability with increasing mRNA dose (Figure S8C).

LNP biophysical characteristics are not correlated with activity in *ex vivo* primary neurons

We evaluated biophysical characteristics of all screened LNP formulations to characterize relationships between LNP composition and potency. Regardless of lipid composition or the method used for LNP loading (rapid-mixing or benchtop mixing), all formulations had RNA encapsulation efficiencies of at least 80% (1.1%), with most formulations approaching near-total RNA entrapment (Figures S9A and S9B). Particle size and polydispersity index (PDI) were also assessed, but because LNP loading using benchtop mixing does not permit measurement of these features due to the relatively low concentration of particles produced by the method, these parameters were evaluated only for mRNA-containing formulations. The PDIs of mRNA-containing LNPs ranged from 0.05 to 0.17 (0.03), indicating the formulations were relatively homogenous (Figure S9C). Particle size measurements were more variable (57.9–117.0 [16.4] nm particle diameter), and although no trends between particle size and LNP composition were apparent, the largest particles within each phospholipid series were generally more potent (Figures S9D–S9G). Further analysis of the relationship between particle diameter and formulation potency revealed no consistent significant correlation between the factors (Figures S9D–S9G). Correlation coefficients also progressively approached zero with increasing mRNA dose, indicating an inconsistent relationship between particle size and efficacy (Figures S9D–S9G). In aggregate, our results show that entrapment of siRNA and mRNA was efficient and consistent across all formulations, and that mRNA LNP particle size and activity were not reliably correlated in this *ex vivo* system. Thus, these aspects of LNP morphology do not inform the patterns of delivery efficacy we report, indicating that alternative characteristics (such as lipid composition) dictate LNP potency in *ex vivo* primary neurons.

LNP activity in *ex vivo* primary neurons does not predict *in vivo* activity in the brain

Ex vivo formulation screening identified distinct LNP compositions most effective for delivering siRNA and mRNA to primary neurons, and indicated that lipid species is a primary determinant of the differential efficacies we report. Separate from the formulation characteristics that impact LNP transfection potency in *ex vivo* primary neurons, successful delivery of therapeutic nucleic acids to the brain requires translatable *in vivo* LNP functionality. Although the correlation between *in vitro* and *in vivo* LNP activity is typically poor in the periphery,[29] a growing number of central nervous system-focused LNP studies have demonstrated more consistent formulation efficacy across *in vitro* and *in vivo* systems; for example, Akita et al. showed equivalent potency of plasmid DNA-containing LNPs *in vitro* in KT-5 murine astrocyte-derived cells and *in vivo* in murine astrocytes and neurons.[17] An *in vitro*-optimized LNP formulation has also been used to successfully deliver FLuc mRNA to astrocytes and neurons *in vivo* via intracerebroventricular infusion,[10] and a single LNP composition has enabled therapeutic siRNA delivery to rat neurons *ex vivo* and *in vivo*.[11] Informed by these studies, we hypothesized that our *ex vivo* primary neuron LNP formulation screens would accurately model LNP activity in the brain *in vivo*.

To assess the correlation between LNP activity in *ex vivo* primary neurons and *in vivo* in the brain, we evaluated the activity of the most efficacious formulations identified by our *ex vivo* primary neuron screens in the brains of adult mice. For siRNA, we evaluated the activity of MC3 DOPG LNPs, and for mRNA, we evaluated the activity of both DODMA DOPE and MF019 DOPC LNPs. For both payloads, the activity of the optimized formulations was compared with the activity of benchmark MC3 DSPC LNPs. All formulations were delivered by stereotaxic bilateral intraparenchymal injection to striatum, a region of primary pathology for a number of genetic neurological disorders including Huntington's disease and GNAO1-dependent early infantile epileptic encephalopathy.[30,31] Due to extremely robust *EGFP* expression levels in transgenic *EGFP* reporter mice, *in vivo* LNP siRNA formulation screening was performed using siRNA targeting the endogenous gene *Htt* (we have previously demonstrated equivalent and effective *Htt* mRNA lowering in primary neurons treated with benchtop-mixed and rapid-mixed Htt siRNA-containing LNPs)[21]; in alignment with *ex vivo* screening studies, FLuc mRNA was used for *in vivo* LNP mRNA studies.

For siRNA, 35.6 (11.0%)–35.9% (7.5%) *Htt* knockdown was measured in striata treated with MC3 DSPC and MC3 DOPG LNPs (Figure 6A). The degree of knockdown achieved by the two formulations was nearly equivalent, demonstrating that *in vivo* LNP siRNA formulation activity was inconsistent with the activity we measured in *ex vivo* primary neurons (Figure 6A). We observed a similar trend in the mRNA screen, where the most robust luminescence measurements were recorded in striata injected with MC3 DSPC LNPs. Less robust luminescence was measured in striata injected with MF019 DOPC LNPs, and DODMA DOPE LNPs, which were the most efficacious for mRNA delivery in primary neurons *ex vivo*, failed to produce a signal distinguishable from the PBS-injected negative control (Figure 6B). Consistent with our *ex vivo* results, LNP biophysical characteristics were not clearly associated with *in vivo* activity (Figures S10A–S10F). Nucleic acid encapsulation exceeded 80% (0.5%) for all formulations (Figures S10A and S10D), and particle diameters were not prognostic of *in vivo* efficacy (Figures S10B, S10C, S10E, and S10F).

In these screens, the most efficacious *in vivo* LNPs contained ionizable cationic lipids with structural and physicochemical similarities: MC3 and MF019 have identical tertiary amine head groups and ester linkages, and highly similar apparent pK_as (6.44 and 6.41, respectively) (Figure 1B).[15,26] Within the pK_a variant LNP series, we evaluated *ex vivo*, the most effective formulations had ionizable cationic lipid apparent pK_as within the range that is also most effective for hepatic delivery *in vivo*,[15] suggesting that the *ex vivo* activity of pK_a variant LNPs may more closely inform *in vivo* LNP activity. To explore this possibility, we evaluated the impact of ionizable cationic lipid pK_a on LNP activity in the brain. As previously performed, LNPs were prepared with MC2, MC3, and MC4 and equimolar blends of MC2 and MC3, and MC3 and MC4, in combination with DSPC phospholipid, to generate five formulations containing

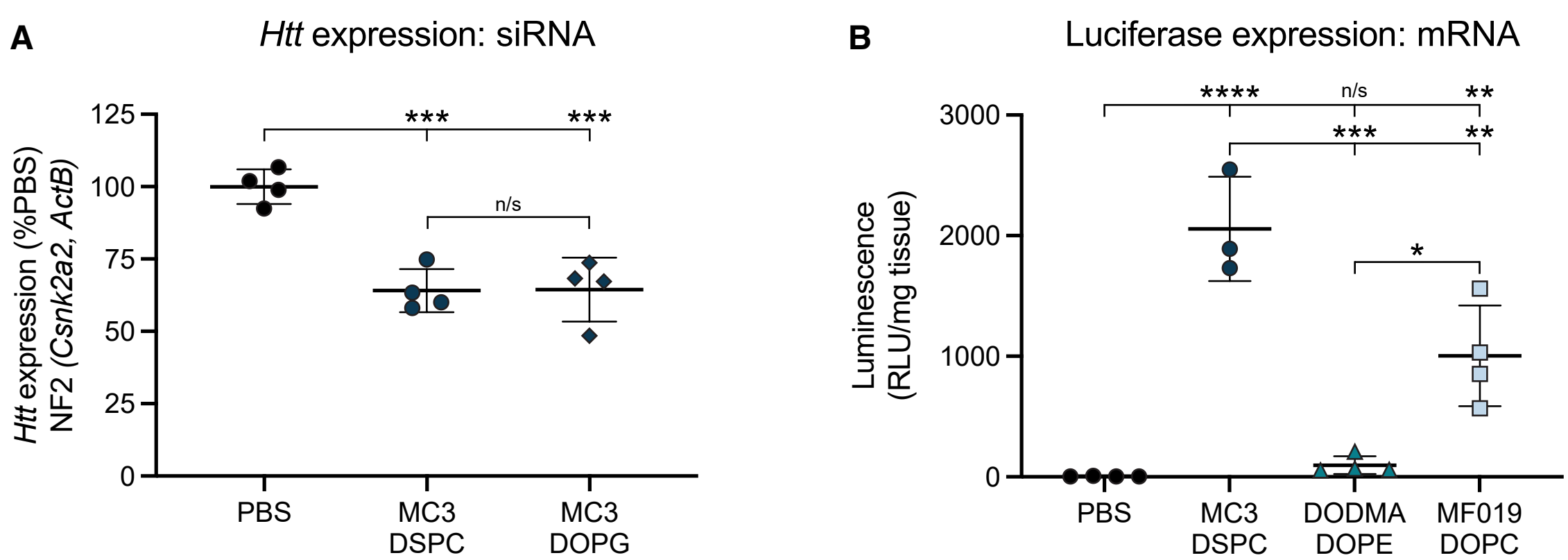

Figure 6. LNP compositions optimized for *ex vivo* primary neurons are not optimal *in vivo* in striatum

To assess correlations between *ex vivo* and *in vivo* LNP activity, the most effective LNP formulations for delivering (A) siRNA and (B) mRNA to *ex vivo* primary neurons were evaluated *in vivo* in murine striatum. (A) The *ex vivo*-optimized LNP siRNA formulation (MC3 DOPG) and benchmark formulation (MC3 DSPC) containing Htt siRNA lowered *Htt* in striatum (one-way ANOVA with Dunnett post-test, PBS vs. MC3 DSPC, $p = 0.0005$; PBS vs. MC3 DOPG, $p = 0.0006$), and the activity of the two formulations was nearly equivalent (unpaired *t* test, $p = 0.9589$). *Htt* expression in the striata of treated mice is normalized to the level of *Htt* expression measured in the striata of PBS-injected control mice. 1,250 ng of Htt siRNA was delivered per 0.5 μL injection. $N = 4$ biological replicates ($N = 2$ technical replicates) per condition. Brains were collected for analysis at seven days post-treatment. (B) The *ex vivo*-optimized LNP mRNA formulations (DODMA DOPE and MF019 DOPC), and benchmark formulation (MC3 DSPC) were compared *in vivo* in striatum. Only MC3 DSPC and MF019 DOPC LNPs produced detectable luminescence (one-way ANOVA with Dunnett post-test, PBS vs. MC3 DSPC, $p < 0.0001$; PBS vs. DODMA DOPE, $p = 0.9431$; and PBS vs. MF019 DOPC, $p = 0.0013$), and the MC3 DSPC formulation was most potent (one-way ANOVA with Tukey post-test, MC3 DSPC vs. DODMA DOPE, $p = 0.0002$; MF019 DOPC vs. DODMA DOPE, $p = 0.0131$; and MC3 DSPC vs. MF019 DOPC, $p = 0.0088$). Luminescence in the striata of treated mice is shown relative to the background signal measured in the striata of PBS-injected control mice and normalized to RLU/mg tissue. 500 ng of FLuc mRNA was delivered per 0.5 μL injection. $N = 4$ biological replicates ($N = 1$ technical replicate) per condition. Brains were collected for analysis at 4 h post-treatment. Data represent the mean (SD).

ionizable cationic lipids with a range of apparent pK_a values (5.64–6.93). siRNA-containing LNP treatment reduced striatal *Htt* expression between 18.4% (4.4%) and 37.6% (9.2%), and, although most formulations induced similar *Htt* lowering (excluding the less-effective MC2 LNPs), the most robust *Htt* knockdown (37.6% [9.2%] *Htt* knockdown) was induced by MC3 LNPs (Figures 7A and S11A). A nearly identical trend was observed with FLuc mRNA-containing LNPs: the strongest luminescent signal (2243.9 [833.5] RLU/mg tissue) was produced by treatment with MC3 LNPs, MC2 LNPs were least effective, and the activity of the MC2/MC3, MC3, MC3/MC4, and MC4 formulations was statistically comparable (Figures 7B and S11B). Consistent with our other results, the LNP biophysical characteristics we measured did not inform *in vivo* activity (Figure S12).

LNPs delivered *in vivo* to murine striatum are taken up by multiple cell types

Previous studies have demonstrated similar performance of LNPs *in vitro* and *in vivo* in the brain[10,11,17]; however, the potency of the LNP formulations we optimized to deliver siRNA and mRNA to primary neurons *ex vivo* did not translate to the brain *in vivo*. We hypothesized this discordance could be explained by the presence of non-neuronal cells *in vivo*, particularly in the context of glial-mediated immune responses that do not occur in *ex vivo* primary neuron cultures. To characterize LNP distribution and activity in the brain *in vivo*, we delivered an MC3 DSPC formulation (the composition with the best *in vivo* activity for both siRNA and mRNA cargoes) containing FLuc mRNA to murine striatum by stereotaxic unilateral injection. Cells expressing luciferase (Luc+) were present throughout the brain parenchyma ipsilateral to the injected hemisphere (Figure S13A), demonstrating that our approach effectively delivered LNPs to the striatum and proximal structures. Luciferase expression was detectable in cells in the corpus callosum (Figure S13B) and adjacent to the lateral ventricle (Figure S13C), and in the striatum in cells with neuron-like morphology (Figure S14D).

The presence of Luc+ cells in both neuron-rich (striatum) and neuron-poor (corpus callosum) regions suggested LNPs were taken up by a variety of cell types. To determine the identity of these Luc+ cells, we used immunostaining to label neurons (NeuN), astrocytes (GFAP), microglia (Iba1), and oligodendrocytes (GST-P) in luciferase-expressing brain sections (Figure 8A). Most co-labelled Luc+ cells were neurons (14.7% [7.1%] NeuN+/Luc+ cells) and oligodendrocytes (13.8% [9.0%] GST-P+/Luc+ cells), while a smaller proportion of Luc+ cells were astrocytes (8.1% [5.1%] GFAP+/Luc+ cells) and microglia (8.9% [5.8%] Iba1+/Luc+ cells) (Figure 8B). These data show that, in addition to neurons, the evaluated MC3 DSPC LNP formulation also effectively delivered mRNA to at least three other major cell types in the brain.

DISCUSSION

Here, we report the development and optimization of effective and non-toxic LNP compositions that enable siRNA and mRNA delivery to primary neurons *ex vivo* and to the brain *in vivo*. We utilized a

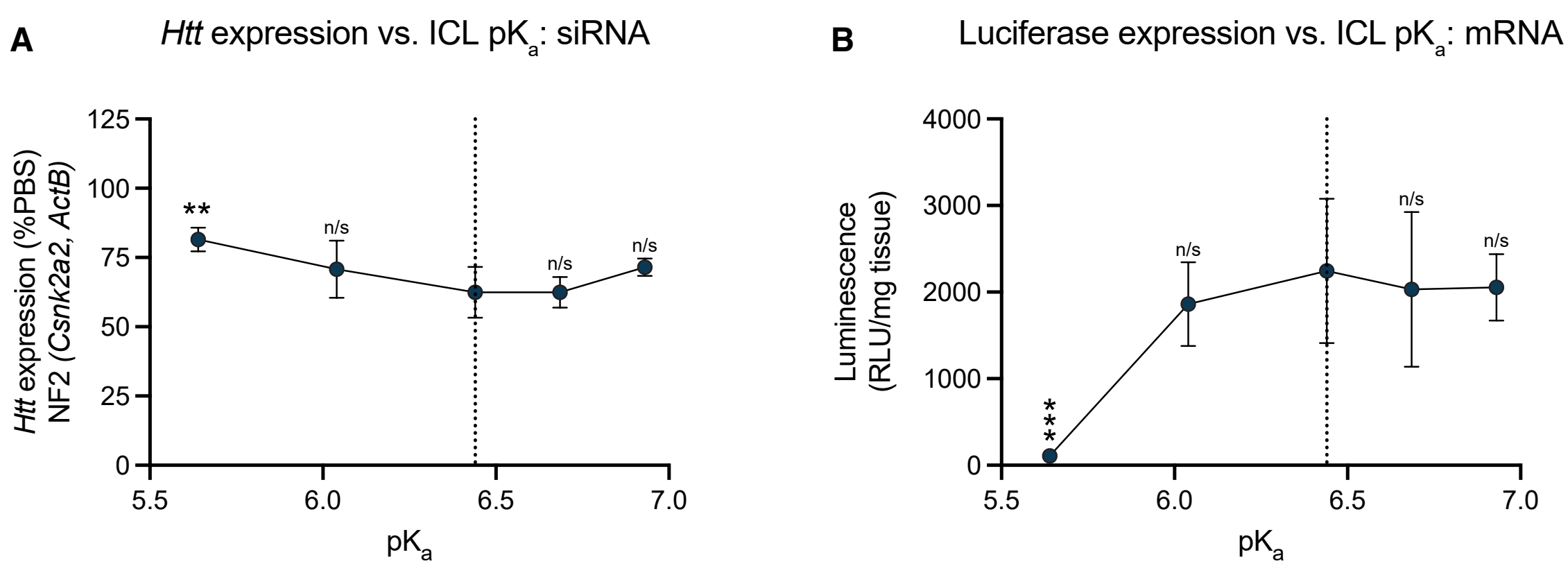

Figure 7. Ionizable cationic lipid apparent pK_a differentially impacts LNP potency *in vivo* in striatum

(A) Htt siRNA-containing pK_a-variant LNPs knocked down *Htt* in striatum with apparent pK_a-dependent efficacy (one-way ANOVA, $p = 0.0094$). The apparent $pK_a = 5.64$ formulation was less potent than the benchmark formulation (denoted by the vertical dotted line at X = 6.44); one-way ANOVA with Dunnett post-test, $p = 0.0059$), while the remaining formulations performed similarly (one-way ANOVA with Dunnett post-test, 6.03 vs. 6.44, $p = 0.3210$; 6.69 vs. 6.44, $p > 0.9999$; 6.93 vs. 6.44, $p = 0.2549$) (see also Figure S11A). *Htt* expression in the striata of treated mice is normalized to the level of *Htt* expression measured in the striata of PBS-injected control mice. 1,250 ng of Htt siRNA was delivered per 0.5 μL injection. $N = 4$ biological replicates ($N = 2$ technical replicates) per condition. Brains were collected for analysis at seven days post-treatment. (B) FLuc-containing pK_a-variant LNPs induced luciferase expression in striatum with apparent pK_a-dependent efficacy (one-way ANOVA, $p = 0.0010$). The apparent pK_a = 5.64 formulation was ineffective compared with the benchmark formulation (denoted by the vertical dotted line at X = 6.44; one-way ANOVA with Dunnett post-test, $p = 0.0007$), while the remaining formulations performed similarly (one-way ANOVA with Dunnett post-test, 6.03 vs. 6.44, $p = 0.7933$; 6.69 vs. 6.44, $p = 0.9649$; 6.93 vs. 6.44, $p = 0.9775$) (see also Figure S11B). Luminescence in the striata of treated mice is shown relative to the background signal measured in the striata of PBS-injected control mice and normalized to RLU/mg tissue. 500 ng of FLuc mRNA was delivered per 0.5 μL injection. $N = 4$ biological replicates ($N = 1$ technical replicate) per condition. Brains were collected for analysis at 4 h post-treatment. Data represent the mean (SD).

novel approach for benchtop siRNA LNP loading that we developed[21] and show microfluidic rapid mixing is most effective for mRNA LNP loading. We systematically evaluated LNP formulations of siRNA and mRNA prepared with a broad range of ionizable cationic lipids and phospholipids to assess the effect of lipid composition on formulation potency and cell viability in primary neurons. We measured nucleic acid encapsulation and particle size to evaluate LNP biophysical characteristics in the context of lipid compositions and corresponding potencies. Finally, we measured the activity of *ex vivo*-optimized LNPs in murine striatum *in vivo* to assess the degree to which LNP screening undertaken in *ex vivo* primary neurons informed *in vivo* activity.

Although the benchtop mixing method we previously developed for LNP loading is efficient for siRNA, mRNA loading using the same method produced LNPs with decreased activity in comparison with a conventionally prepared formulation. Relative to siRNA, differences in mRNA molecular size (1,921 nucleotides vs. a 42 nucleotide 21-mer), structure (single-stranded vs. double-stranded), chemical modifications (e.g., pseudouridine),[32] and resulting variations in charge density may necessitate the fast flow rate and turbulence inherent to rapid-mixing to produce highly efficacious LNPs.[33] siRNA and mRNA formulations were also prepared using different amine-to-phosphate (N/P) ratios (N/P = 3 for siRNA and N/P = 6 for mRNA); as a result, the efficacy of LNP loading using the two methods (and patterns in the resultant biological activity of siRNA- and mRNA-containing formulations prepared using the same ionizable cationic lipid and phospholipid species) cannot be compared directly. It is clear, however, that while LNPs are amenable to accommodating various payloads, they must be appropriately designed and prepared for each payload type.

Compared witho LNPs with the same composition as the LNP siRNA drug Onpattro, our formulation screening strategy improved *ex vivo* neuronal *EGFP* reporter knockdown over 1.7-fold and reporter mRNA expression over 22-fold at equivalent nucleic acid doses. Different LNP compositions were optimal for delivery of each payload: MC3 DOPG LNPs were most effective for siRNA delivery, whereas DODMA DOPE LNPs were most effective for mRNA delivery. Neuronal viability was differentially affected by nucleic acid dose and formulation, and was relatively well-preserved with most LNP treatments.

To assess the effect of ionizable cationic lipid species on neuronal transfection, LNPs prepared with MC3 were compared with formulations containing DODMA and MF019, and, for siRNA only, the permanently cationic lipid DOTMA. DODMA-containing LNPs were less potent than the MC3 benchmark formulation for siRNA delivery, but were highly efficient for mRNA delivery. We observed a similar effect for MF019 formulations, where replacement of MC3 with MF019 reduced the effectiveness of siRNA-mediated *EGFP* knockdown, but strikingly improved mRNA formulation efficacy. Thioether-containing cationic lipids such as MF019 have been shown to modestly improve LNP transfection efficiency compared with a cationic lipid containing monounsaturated 18:1 oleyl chains by modifying membrane fluidity and the supramolecular packing

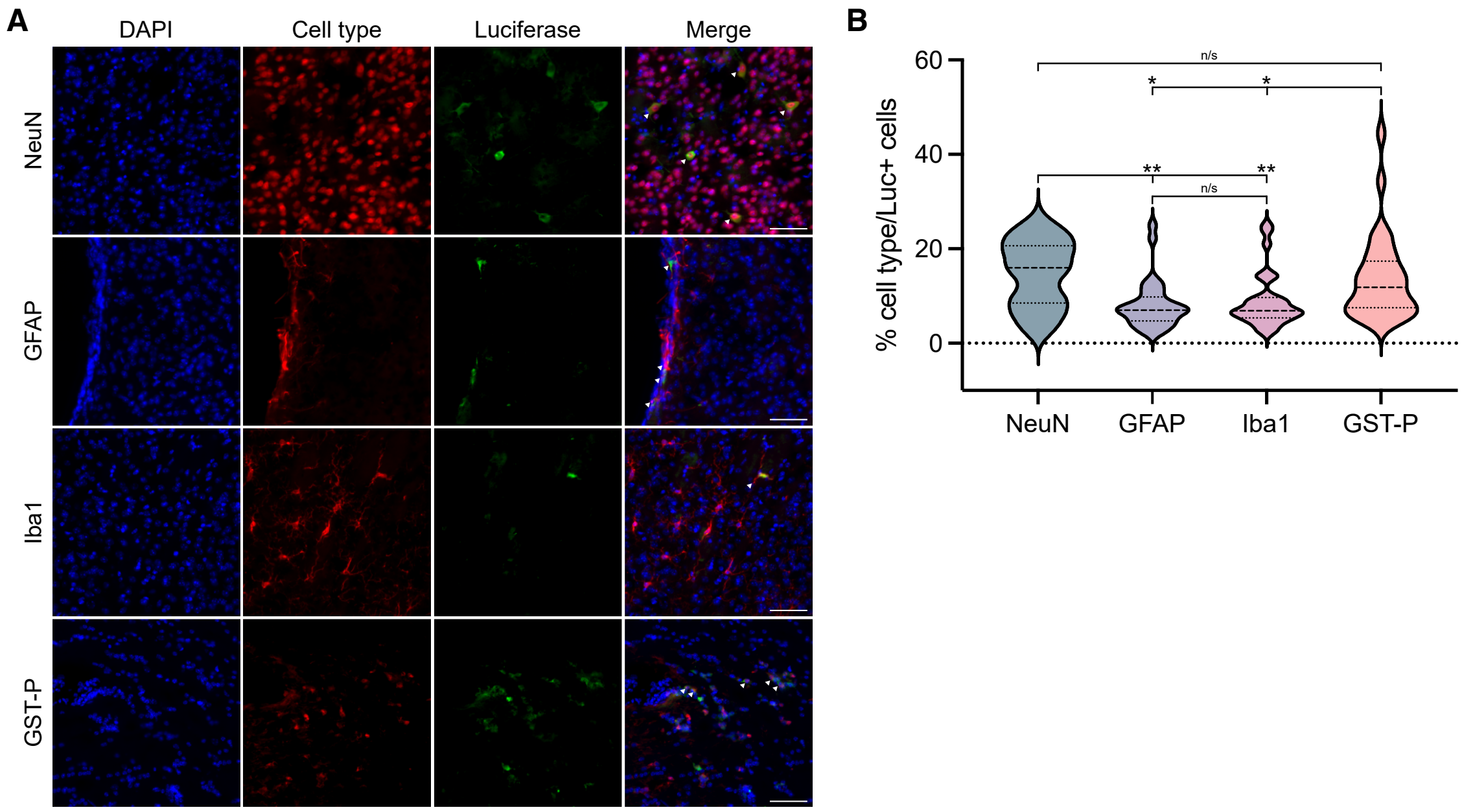

Figure 8. Neurons, astrocytes, microglia, and oligodendrocytes take up LNPs *in vivo*

(A) Following direct intrastriatal injection of MC3 DSPC LNPs containing FLuc mRNA, luciferase-expressing brain cells co-localized with cellular markers for neurons (NeuN), astrocytes (GFAP), microglia (Iba1), and oligodendrocytes (GST-P). Representative images of luciferase expression and cell type-specific markers for NeuN, GFAP, Iba1, and GST-P are shown. Cell nuclei are shown in blue, luciferase-expressing cells are shown in green, and cell type-specific markers are shown in red. Examples of luciferase expression and cell type-specific marker co-localization are indicated by white arrows for each cell type. Scale bars, 50 μm. (B) Of luciferase-expressing brain cells, 14.7% co-localized with NeuN+ cells, 8.1% co-localized with GFAP+ cells, 8.9% co-localized with Iba1+ cells, and 13.8% co-localized with GST-P+ cells. Luciferase-expressing cells most frequently co-localized with NeuN+ cells (one-way ANOVA with Tukey post-test, NeuN vs. GFAP, $p = 0.0027$; NeuN vs. Iba1, $p = 0.0099$) and GST-P+ cells (one-way ANOVA with Tukey post-test, GST-P vs. GFAP, $p = 0.0103$; GST-P vs. Iba1, $p = 0.0341$). An equivalent proportion of luciferase-expressing cells co-localized with NeuN+ and GST-P+ cells (one-way ANOVA with Tukey post-test, NeuN vs. GST-P, $p = 0.9577$) and GFAP+ and Iba1+ cells (one-way ANOVA with Tukey post-test, GFAP vs. Iba1, $p = 0.9752$). $N = 3$ biological replicates ($N = 27$–30 technical replicates/cell type-specific marker). 500 ng of FLuc mRNA was delivered per 0.5 μL injection. Brains were collected for analysis 4 h post-treatment. Data represent the mean (SD).

of lipids and nucleic acid, which must be balanced to ensure nucleic acid encapsulation and intracellular cargo release are both sufficient.[34] The optimal parameters for each of these factors for siRNA and mRNA delivery are likely unique due to differences in molecular size and structure between the molecules, which may contribute to the potency differences we observed between MF019 formulations containing siRNA and mRNA. Although DODMA contains monounsaturated 18:1 oleyl chains, siRNA and mRNA cargoes may contribute to the differential efficacy of DODMA-containing LNPs in a similar manner. Additional structural and functional studies are required to more clearly characterize the higher-order interactions that occur between ionizable cationic lipids, nucleic acids, and other LNP lipid excipients that impact neuronal delivery. MF019 was assessed as an alternative to MC3; MF019 acyl chain thioether moieties were designed to promote intracellular hydrolysis and accelerate lipid clearance from transfected cells. Although MF019 did not meaningfully impact neuronal viability *ex vivo*, the benefits of improved clearance may become apparent in the brain *in vivo*, or at higher nucleic acid doses than we evaluated.

The addition of DOTMA to MC3-based siRNA formulations reduced knockdown efficiency and increased toxicity, likely due to the presence of a permanent positive charge.[35] Although incorporating permanent cationic lipids in LNP formulations has been shown to improve nucleic acid encapsulation, this addition may also impede LNP activity by sequestering siRNA within particles and therefore limiting intracellular release.[36,37] While we observed increased siRNA encapsulation in formulations containing progressively larger molar percentages of DOTMA, siRNA encapsulation was comparable between MC3-only LNPs and LNPs containing a larger proportion of MC3 than DOTMA. It is likely that the presence of a small molar percentage of DOTMA in combination with MC3 prevented or limited the intracellular release of siRNA without meaningfully improving encapsulation, thus reducing the effective siRNA dose delivered by those formulations. The modest *EGFP* knockdown induced by LNPs containing intermediate DOTMA percentages may be attributed to increased siRNA encapsulation coupled with partial intracellular release enabled by MC3, while the lack of knockdown associated with the 45% DOTMA formulation may be attributed to failed intracellular siRNA release (despite a 99.1% [0.9%] encapsulation efficiency). The poor performance of DOTMA-containing siRNA LNPs and the notable toxicity of the formulations (which is a well-characterized feature of nucleic acid delivery vectors containing permanently charged cationic lipids)[38] do not

support the use of DOTMA-containing LNPs for siRNA delivery to primary neurons; as such, we did not evaluate DOTMA LNPs for mRNA delivery.

In contrast with *in vivo* gene silencing potencies measured in hepatocytes following systemic LNP administration,[15] our results show that higher ionizable cationic lipid apparent pK_as do not substantially impact the magnitude of LNP-mediated *EGFP* knockdown in *ex vivo* primary neurons (Figure 2C; Table S1). This is objectively unsurprising because these respective *in vivo* and *ex vivo* datasets comprise a number of important differences, including the absence of a neuronal competent immune response. The direct application of LNPs to target cells also lowers the delivery barrier for slightly positively charged particles (i.e., for formulations containing ionizable cationic lipids with higher apparent pK_as). For mRNA-containing LNPs, however, treatment with the MC3-only formulation (pK_a = 6.44) in the pK_a variant screen resulted in the most robust FLuc expression, while lower and higher pK_a variant formulations were statistically suboptimal at all doses (Figures 3B and S6). In contrast, DODMA-containing LNPs were more effective for mRNA delivery than those containing MC3. DODMA has an estimated pK_a of 6.59–7.0,[39,40] indicating that aspects of lipid chemistry aside from apparent pK_a contribute to LNP efficacy *ex vivo*. Considered together, these results suggest that mRNA-containing LNP systems may be more sensitive to ionizable cationic lipid apparent pK_a than systems containing siRNA, or that the methods used to assess the activity of each formulation type are not directly comparable. One consideration is the differences in the relative dynamic ranges of the outcome measures used to assess knockdown and expression levels: mRNA delivery typically exacerbates differences in activity between formulations (as every successful delivery attempt results in a positive signal), whereas knockdown requires siRNA delivery, engagement with the cognate mRNA, and RISC activation. The variable impact of apparent pK_a upon LNP activity in *ex vivo* primary neurons and *in vivo* in the brain is also demonstrated by the lack of statistically significant correlation between LNP activity and apparent pK_a when apparent pK_a is analyzed as a continuous independent variable (Table S1; siRNA LNP r^2 = 0.2586; mRNA LNP r^2 = 0.03549 [0.03 μg/mL FLuc mRNA], 0.05337 [0.1 μg/mL FLuc mRNA], 0.1662 [0.3 μg/mL FLuc mRNA], and 0.2661 [1.0 μg/mL FLuc mRNA]). While our results show that apparent pK_a affects LNP activity both *ex vivo* and *in vivo*, it is clear that other chemical and biological factors also contribute to formulation efficacy; ionizable cationic lipid apparent pK_a, therefore, is an important consideration when designing LNP systems for RNA delivery to the central nervous system, but is not the sole determinant of potency.

Enhanced potency of LNPs prepared with DOPC and DOPE is well-documented,[28,41,42] and both siRNA and mRNA LNP systems prepared using DOPC and DOPE transfected *ex vivo* primary neurons more effectively than those containing DSPC. DSPC is comprised of a quaternary amine head group and saturated phosphatidylcholine acyl chains connected by ester linkages, while DOPE has a primary amine head group and acyl chains with 1° of unsaturation (Figure S1). The features of DOPE favor the formation of hexagonal H_{II} phase lipid structures, which increase membrane fusogenicity and are critical for intracellular nucleic acid release from endocytosed LNPs.[41,43,44] DOPC was selected as an intermediate structural control between DPSC and DOPE; like DSPC, it contains a quaternary amine head group and, like DOPE, has acyl chains with 1° of unsaturation. Relative to DOPC formulations, the improved activity of DOPE LNPs may be attributed to the smaller primary amine head group, which confers cone-shaped geometry to DOPE and further promotes H_{II} phase formation.[45]

Because DSPC, DOPC, and DOPE are uncharged zwitterionic molecules, DOPG (which has a glycerol head group and unsaturated acyl chains) was evaluated as an alternative, negatively charged phospholipid. LNP siRNA systems containing DOPG were superior for neuronal siRNA delivery in combination with every ionizable cationic lipid we evaluated, and, depending on dose, were also effective for mRNA delivery. Anionic nanocomplexes containing DOPG have been used for siRNA delivery *in vitro* to Neuro-2A cells and *in vivo* in the brain and while DOPG may have contributed to formulation efficacy in these studies, the concurrent presence of other elements that also modify potency (such as PEGylated lipids, targeting peptides, and *in vivo* convection enhanced delivery) makes it difficult to elucidate a DOPG-specific transfection effect that also applies to our results.[46–48] For mRNA delivery, treatment with DOPG LNP formulations at higher concentrations did not improve luciferase expression and, with formulations containing MC3 and MF019, the 1.0 μg/mL mRNA dose produced a weaker reporter signal. This contradictory effect is likely caused by the PEG lipid, which dissociates from LNPs after treatment and, at sufficiently high concentrations, can impede *in vitro* transfection efficiency by interfering with binding interactions between LNPs and target cells.[16,49] Additional investigations concerning the mechanism by which DOPG improves LNP neuronal delivery efficacy are required to better inform the rational design of future neuron-specific LNP formulations.

At equivalent nucleic acid doses, we observed more toxicity associated with siRNA formulations than mRNA formulations. Although nucleic acid (and corresponding lipid) doses significantly affected neuronal health in siRNA and mRNA screens, mRNA LNPs were prepared at a higher N/P ratio than siRNA LNPs, indicating that lipid dose was not primarily responsible for this effect. Instead, saturation of endogenous RNAi machinery may have occurred at higher siRNA doses, which can disrupt endogenous miRNA processing and cause toxic effects.[50]

Particularly at the lowest nucleic acid doses we tested, LNP treatment modestly increased neuronal viability compared with untreated control neurons from the same culture, which may have been driven by ApoE addition. Supplementation with exogenous human ApoE isoforms 3 and 4 has been shown to exert neurotrophic and neuroprotective effects on rat hippocampal neuron cultures (assessed by neurite outgrowth and metabolic activity) through a mechanism

independent from ApoE-LDL receptor binding, suggesting that *ex vivo* primary neurons may benefit from the addition of ApoE to culture medium.[51] Other evidence suggests that ApoE4 (used in our study) impedes neurite outgrowth of murine cortical neurons through a mechanism that depends upon ApoE-LDL receptor binding, which requires the complexation of ApoE with additional lipids.[52] At the lowest nucleic acid concentrations we assessed, ApoE may be present in excess of the exogenous lipids in the LNP suspension, and could therefore be present both as free apolipoprotein and adsorbed to LNPs. Because the neurotrophic effects of ApoE are independent from ApoE-LDL receptor family interactions, free ApoE may improve neuronal health and account for the viability increases we observed. This effect may be dampened at higher doses, where the proportion of free ApoE is smaller, or increased lipid and nucleic acid concentrations make associated toxicities more prevalent. With some formulations, we also observed a subtle improvement in the viability of primary neurons treated with LNPs at 1.0 μg/mL mRNA, but not siRNA. This effect may be driven by exogenous cholesterol, which has been shown to promote the survival of rat primary neurons treated with inhibitors of cholesterol biosynthesis.[53] The LNP formulations used in our study were prepared with 39 mol% cholesterol, and the N/P ratios used for mRNA (N/P = 6) and siRNA (N/P = 3) LNPs mean that, at 1.0 μg/mL nucleic acid, LNP mRNA treatment introduced 0.24 nmol cholesterol/neuron while LNP siRNA treatment introduced 0.12 nmol cholesterol/neuron. Our data suggest the quantity of cholesterol introduced by LNP siRNA treatment at 1.0 μg/mL siRNA is insufficient to impact the viability of primary neurons, while the quantity of cholesterol introduced by LNP mRNA treatment at 1.0 μg/mL mRNA may support neuronal health. This effect was not observed for all LNP mRNA-treated primary neurons at 1.0 μg/mL mRNA; in cases where neuronal health declined with increased mRNA dose, it is likely that lipid-associated toxicity superseded the cholesterol-associated viability benefit.

With the exception of the pK_a variant screens, we did not observe consistent activity trends between *ex vivo*-optimized LNPs and the formulations we assessed *in vivo*, which may be a product of nuanced differences between the model systems. The primary neurons cultured for *ex vivo* formulation screening were isolated from microdissected cortex tissue, while *in vivo* screens were conducted in striatum. The relative proportions of neuronal subtypes in these regions are distinct: cortical neuron populations consist of approximately 20% GABAergic interneurons and 80% glutamatergic pyramidal neurons,[54] while striatal neuron populations consist of approximately 95% GABAergic medium spiny projection neurons, 1% cholinergic interneurons, and 4% aspiny GABAergic interneurons.[55] *Ex vivo* primary cortical neurons were also isolated from E17.5 cortices, while *in vivo* screening was undertaken in adult murine striatum. Although *ex vivo* LNP screens were conducted using metabolically stable neurons (Figure S14A) and *Ldlr* expression in embryonic and adult murine brain is comparable,[56] inherent differences in neuronal subtypes and cellular maturity may have contributed to the discrepancies in LNP activity we report. It is also likely that the presence of non-neuronal cells *in vivo* contributed to the lack of correlation we observed between *ex vivo* and *in vivo* LNP activity. While 14.7% (7.1%) of Luc+ cells *in vivo* were neurons, an equivalent proportion (13.8% [9.0%] GST-P+/Luc+ cells) were oligodendrocytes, and an additional 8.1% (5.1%) and 8.9% (5.8%) were, respectively, astrocytes and microglia.

The presence of differing lipoprotein species and abundance in primary neuron culture medium and the brain may have also influenced the *in vivo* translatability of our *ex vivo* formulation screening results. Studies conducted both *in vitro* using media prepared with $ApoE^{-/-}$ mouse serum and *in vivo* in $ApoE^{-/-}$ mice have clearly demonstrated that ApoE is required for LNP-mediated RNA delivery[7,16]; however, LNP activity *in vitro* is also impacted by the type of serum with which cells are cultured,[16,57] and, in general, discordance between *in vitro* and *in vivo* LNP potencies may be driven by the species-specific protein compositions of sera used in mammalian cell culture. Although primary neurons are not cultured in serum, recombinant ApoE is added to defined culture medium to facilitate LNP uptake *ex vivo*; *in vivo,* however, over 700 endogenous proteins, including ApoE, are measurable in murine cerebrospinal fluid.[58] While ApoE is present in both systems, ApoE binding affinity and resultant LNP uptake and activity *in vivo* may be altered by the complex protein composition of cerebrospinal fluid; as a result, LNP activity *in vivo* may also be impacted.

In our assessment of cell-type-specific LNP uptake in the brain, 45.5% of Luc+ cells co-localized with NeuN, GFAP, Iba1, and GST-P; therefore, a substantial proportion of Luc+ cells were not neurons, astrocytes, microglia, or oligodendrocytes. The markers used for cell type labeling may have limited identification of cells within the same lineage at various stages of maturation. For example, NeuN and GST-P, respectively, label mature neurons and oligodendrocytes; therefore, these markers do not label neural progenitor cells (NPCs) or oligodendrocyte precursor cells (OPCs), and the distribution pattern of the remaining Luc+ cells suggests a portion of them may be NPCs and OPCs. Many Luc+ cells were localized in the dorsolateral subventricular zone (SVZ), a region from which NPCs originate[59]; OPCs may also be borne from the SVZ in adult mammalian brain.[60] Proximity to the lateral ventricle also suggests a subset of Luc+ cells may be ependymal.[61]

The strong concentration of Luc+ cells within the corpus callosum indicates LNPs delivered directly to the brain parenchyma have affinity for white matter. A similar LNP distribution pattern has been reported following direct intrastriatal injection of an LNP formulation with a distinct lipid composition and molar ratio from the one we evaluated[62]; thus, LNP brain distribution may be at least partially agnostic of lipid composition. Whether white matter expression results from LNP transport through the parenchyma or reflux through the injection track augments delivery to tissues proximal to the needle track remains to be determined. Regardless, these findings suggest LNP-based therapies may be useful for treating CNS disorders associated with white matter.

In summary, our LNP formulation development approach improved *EGFP* reporter knockdown over 1.7-fold and FLuc mRNA expression over 22-fold in *ex vivo* primary neurons relative to a benchmark control formulation. Different LNP compositions were optimal for delivering each payload: MC3 DOPG LNPs were most effective for siRNA delivery, while DODMA DOPE LNPs were most effective for mRNA delivery. These formulations dramatically improved the efficiency of siRNA and mRNA delivery to neurons without substantially worsening neuronal health, demonstrating that LNPs are an effective and safe modality for delivering nucleic acid to *ex vivo* primary neurons. Because they enable gene knockdown and exogenous mRNA expression in the absence of toxicity, these optimized formulations will be useful tools for enabling post-treatment proof-of-concept regulatory and phenotypic studies in *ex vivo* primary neurons that may, in turn, accelerate the development of nucleic acid-based therapies for genetic neurological disorders affecting neurons. Although we report that lipid species-associated LNP activity in *ex vivo* primary neurons does not directly correlate with the activity of LNPs in the brain *in vivo*, we demonstrate a pattern of LNP activity associated with ionizable cationic lipid apparent pK_a that is consistent across both siRNA- and mRNA-containing LNPs and *ex vivo* and *in vivo* model systems, and show that LNPs effectively deliver RNA to a variety of cell types in the brain *in vivo*. While apparent pK_a is not the only factor that affects LNP potency in the brain, our results demonstrate that ionizable cationic lipids with apparent pK_a values between 6.03 and 6.93 may be suitable for use in LNP formulations designed for brain delivery. To our knowledge, this is the first evidence demonstrating the impact of apparent ionizable cationic lipid pK_a on LNP activity in the absence of serum and in the brain, which may inform the rational design of future LNP-enabled brain gene therapies.

MATERIALS AND METHODS

Materials

The ionizable cationic lipid 2,2-dilinoleyl-4-(2-dimethylaminoethyl)-[1,3]-dioxolane (KC2) was synthesized by Biofine International (Vancouver, BC). The ionizable cationic lipids MC2, MC3, and MC4 were synthesized as previously described.[15] The ionizable cationic lipid MF019 was synthesized as previously described.[26] The cationic lipid DOTMA and DSPC, DOPC, DOPE, and DOPG were purchased from Avanti Polar Lipids (Alabaster, AL). The ionizable cationic lipid DODMA was purchased from Cayman Chemicals (Ann Arbor, MI). Cholesterol was purchased from Sigma-Aldrich (St Louis, MO). (R)-2,3-bis(tetradecyloxy)propyl-1-(methoxy polyethylene glycol 2000) (PEG-DMG) was synthesized as previously described.[63] Structures of the cationic lipids and phospholipids used are shown in Figures 1 and S1, respectively.

Assay development

The longitudinal viability of primary neurons isolated from both mouse strains was measured to establish appropriate time points for LNP treatment and sample collection (Figure S14A). In both primary neuron genotypes, viability measurements (inferred based on ATP abundance) increased from DIV3 to DIV7, indicating high metabolic activity during neuronal maturation. Viability stabilized in mature primary neurons between DIV7 and DIV10[64] and progressively declined at DIV14 and DIV21, signifying cell death. All subsequent LNP treatments were performed using mature, metabolically stable primary neurons at DIV7, and reporter expression was assayed no later than DIV10.

LNP uptake is dependent upon adsorption of the lipoprotein ApoE, which is abundant in serum and facilitates endocytosis by binding LDL family receptors.[6–9] Because primary neurons are cultured and maintained *ex vivo* in serum-free medium, we assessed the effect of adding exogenous recombinant ApoE to culture medium on neuronal LNP uptake using an LNP formulation containing FLuc mRNA. Luciferase expression was significantly enhanced by the presence of ApoE, demonstrating the requirement of ApoE for effective LNP endocytosis by primary neurons (Figure S14B). To establish a protocol for ApoE addition to primary neuron culture medium, luciferase expression was measured in neurons where ApoE was added either together with, or immediately after, LNP treatment (Figure S14C). The timing of ApoE addition did not significantly impact luciferase expression, and all subsequent primary neuron treatments were performed by diluting, in order, ApoE and LNP formulations in culture medium before application to cells.

LNP preparation: Benchtop mixing

To prepare empty TCVs for benchtop mixing, lipids were dissolved in ethanol at a final combined concentration of 20 mM and combined with a 25 mM sodium acetate pH 4 aqueous buffer using a T-junction microfluidic mixer at a total flow rate of 20 mL/min and a flow rate ratio of 3:1 v/v aqueous:organic phases. The resulting suspension was dialyzed overnight against a 1,000-fold volume of 25 mM sodium acetate pH 4 buffer. To load particles, empty TCVs were combined with siRNA at 0.058 μg siRNA or 0.029 μg mRNA per μmole lipid. Lipid solutions were pipette-mixed with siRNA and incubated at room temperature for 10 min. Culture medium was added to the incubated TCV suspension before primary neuron treatment.

LNP preparation: Rapid mixing

To prepare LNPs loaded with nucleic acid, lipids were dissolved in ethanol at a final combined concentration of 10 mM. Purified siRNA and mRNA were dissolved in 25 mM sodium acetate pH 4 buffer to achieve the appropriate N/P ratio (siRNA = 3 and mRNA = 6). Lipid and nucleic acid solutions were combined using a T-junction microfluidic mixer at a total flow rate of 20 mL/min, and a flow rate ratio of 3:1 v/v aqueous:organic phases. The resulting suspension was dialyzed overnight against a 1,000-fold volume of PBS pH 7.4. Following dialysis, formulations were concentrated using Amicon centrifugal filtration units (Millipore Sigma, Burlington, MA) with a 10 kDa NMWCO.

LNP preparation: Compositions and nucleic acid cargoes

To compare the activity of LNPs prepared using rapid mixing and benchtop mixing, LNPs were formulated at 50/10/38.5/1.5 mol% KC2, DSPC, cholesterol, and PEG lipid to increase particle stability.[65] For formulation screening, all LNPs were formulated at 50/10/39/1

mol% ionizable cationic lipid, phospholipid, cholesterol, and PEG lipid. All siRNA formulation screening experiments were performed using LNPs loaded by benchtop mixing, and all mRNA formulation screening experiments were performed using LNPs prepared by rapid mixing. For all formulation screening studies, LNP formulations of siRNA were screened at 0.01 μg/mL GFP siRNA, while mRNA-containing formulations were screened across a range of mRNA doses (0.03–1.0 μg/mL). siRNA against GFP (Sense: 5′-ACAUGAAGCAGCACGACUUTT-3′; Antisense: 5′-AAGUCGUGCUGCUUCAUGUTT-3′) was purchased from Integrated DNA Technologies (Coralville, IA). Pre-designed Silencer Select siRNA against Htt, ID s67440 (Sense: 5′-GGCCUUACCUGGUGAAUCUTT-3′; Antisense: 5′-AGAUUCACCAGGUAAGGCCTG-3′) was purchased from ThermoFisher Scientific (Burnaby, British Columbia). *Photinus pyralis* FLuc (NCBI Gene ID: 116160065) mRNA incorporating an m7G cap analog, full substitution with 5-methoxyuridine-5′-triphosphate, and a poly(A) tail length of ∼100 nucleotides was purchased from APExBIO (Houston, TX) and Trilink BioTechnologies (San Diego, CA).

LNP characterization

Number-weighted LNP diameter was measured using dynamic light scattering with a Malvern Zetasizer NanoZS (Worcestershire, UK) instrument. Nucleic acid encapsulation efficiency was measured using the Quant-iT RiboGreen RNA assay (Life Technologies, Burlington, ON). LNP formulations were diluted in 1X TE (Invitrogen) alone, or 1X TE + 1% Triton X-100 (Sigma-Aldrich). Fluorescence intensity (Ex/Em: 485/510 nm) was measured following RiboGreen reagent addition using a POLARstar Omega microplate reader (BMG Labtech) to quantify free nucleic acid in intact (1X TE only) and lysed (1X TE + 1% Triton X-100) LNPs. Total lipid content was evaluated by cholesterol quantification using the Cholesterol E assay (Wako Chemicals, Richmond, VA). Nucleic acid concentration was measured by absorbance at 260 nm.

Animals

Transgenic *EGFP* C57BL/6 mice were a generous gift from Dr. Colin Ross. C57BL/6 (JAX #000664) mice were purchased from The Jackson Laboratory (Bar Harbor, ME). Mice were housed on ventilated racks in a specific pathogen-free barrier facility under a 12h light/dark cycle. Prior to breeding, mice were group-housed with littermates to a maximum of five mice per cage and given free access to food and water. To generate embryos for primary neuron cultures, male and female mice of appropriate ages were paired in clean single cages. Breeding pairs were separated immediately following visual detection of a seminal plug in the vaginal canal, and pregnant female mice were single-housed until collection. Mixed-sex cohorts of wild-type C57BL/6 mice between 3 and 6 months of age were used for all *in vivo* studies. All animal work was carried out with the approval of the Canadian Council for Animal Care and the University of British Columbia's Animal Care Committee (Protocol #A22-0167).

Primary neuron culture

Primary cortical neuron cultures were derived from wild-type and transgenic *EGFP*-expressing C57BL/6 mice at embryonic day 17.5. Cortices were isolated by dissection in ice-cold 1X Hank's balanced salt solution (HBSS; Gibco) and collected in Hibernate E medium (BrainBits) supplemented with 0.5 mM L-glutamine (Cytiva). Tissue was digested in a 0.05% (v/v) trypsin (Cytiva) solution in a 37°C water bath for 15 min. Following trypsin inactivation by fetal bovine serum (FBS) (Hyclone) addition, digested tissue was dissociated by trituration using a constrained 5-mL serological pipette and filtered through a 40-μm cell strainer (Falcon). Filtered cells were pelleted by centrifugation for 5 min at 800 RPM, washed once with 1X HBSS to remove FBS, and resuspended in warmed Neurobasal medium (Gibco) supplemented with 2% (v/v) 50X B27 (Gibco), 0.5 mM L-glutamine (Cytiva), and 0.5 mg/mL penicillin/streptomycin (Cytiva). Neurons were plated in poly-D-lysine-coated 24-well and 96-well plates (Falcon; Corning) at 1.5×10^5 cells/mL and maintained in a humidified atmosphere at 37°C and 5% CO_2. Hibernate E medium was purchased from BrainBits (Springfield, IL). All other cell culture reagents were purchased from Life Technologies (Burlington, ON).

Primary neuron LNP transfection

Neurobasal medium (Gibco) supplemented with 0.5 μg/mL recombinant human ApoE4 (PeproTech)[11] was used to dilute LNP formulations. To treat neurons, 50% of the plating medium was removed from each well and replaced with an equivalent volume of diluted LNP suspension. The reported nucleic acid doses and ApoE4 concentration represent the final concentration(s) in each well. Primary neurons were maintained *in vitro* for seven days prior to LNP treatment. Neurons treated with LNP siRNA formulations were collected for analysis 72 h following treatment, and neurons treated with LNP mRNA formulations were assayed after 24 h.

Quantitative RT-PCR

Primary neurons in 24-well plates were collected for RNA extraction by washing with 1X PBS (Gibco), scraping in RNA lysis buffer (Invitrogen) containing 1% 2-mercaptoethanol, and snap-freezing on dry ice. Total RNA was extracted from thawed samples using a PureLink RNA Mini kit (Invitrogen) according to the manufacturer's instructions. PureLink DNase (Invitrogen) was used to degrade residual genomic DNA on preparation columns to increase RNA yield and purity. RNA concentrations were measured using a NanoDrop 2000 spectrophotometer (Thermo Scientific). Reverse transcription was performed using the SuperScript VILO cDNA Synthesis Kit (Invitrogen). Amplification of cDNA was performed using FastSYBR Green Master Mix (Applied Biosystems) and the StepOne Plus Real-Time PCR System (Applied Biosystems). All samples were run in duplicate. mRNA levels were quantified by standard curve using 10-fold serial dilutions prepared from untreated control samples. Relative quantities of target mRNA were calculated as the ratio between target mRNA abundance and a normalization factor created using two control genes (*Csnk2a2* and *ActB*) based on GeNorm.[66] Quantitative RT-PCR primers were purchased from Integrated DNA Technologies (Coralville, IA). Primer sequences are listed in Table S2. All other quantitative RT-PCR reagents were purchased from Life Technologies (Burlington, ON).

Primary neuron viability assay

The viability of LNP siRNA-treated neurons was assayed 72 h after treatment using the CellTiter-Glo luminescent cell viability assay system (Promega) according to the manufacturer's instructions. Neuron plating/treatment medium was removed, replaced with 100 μL of warmed Neurobasal medium (Gibco), equilibrated to room temperature, and combined with an equivalent volume of CellTiter-Glo reagent. Cells were lysed by mixing on an orbital shaker for two minutes and incubated on the benchtop for an additional 10 min. Luminescence was measured using a POLARstar Omega microplate luminometer (BMG Labtech). The CellTiter-Glo luminescent cell viability assay system kit and associated reagents were purchased from Promega (Madison, WI).

Primary neuron luciferase assay

Luciferase assays were performed 24 h after LNP treatments. Luminescence was measured using the SteadyGlo Luciferase Assay System (Promega) according to the manufacturer's instructions. Neuron plating/treatment medium was removed, and neurons were gently washed with 1X PBS (Gibco) and scraped in 1X Glo Lysis Buffer (Promega). Equal volumes of neuron lysate and SteadyGlo luciferase reagent were combined in a 96-well plate (Corning), mixed on an orbital shaker for 30 s, and incubated on the benchtop for five minutes. Luminescence was measured using a POLARStar Omega microplate luminometer (BMG Labtech). The SteadyGlo Luciferase Assay System kit and associated reagents were purchased from Promega (Madison, WI).

Primary neuron combined viability and luciferase assay

For LNP siRNA-treated neurons, viability and luminescence were measured in succession using the ONE-Glo+Tox Luciferase Reporter and Cell Viability Assay kit (Promega) according to the manufacturer's instructions. Neuron plating/treatment medium was removed, replaced with 100 μL of warmed Neurobasal medium (Gibco), equilibrated to room temperature, combined with 20 μL of CellTiter-Fluor reagent (prepared as a 5X solution of GF-AFC substrate diluted in assay buffer), and incubated for 30 min at 37°C. Fluorescence intensity (Ex/Em: 400/510 nm) was quantified using a POLARstar Omega microplate reader (BMG Labtech). To measure luminescence, 100 μL of ONE-Glo reagent was added to each well, incubated for three minutes, and assayed using a POLARstar Omega microplate luminometer (BMG Labtech). The ONE-Glo+Tox Luciferase Reporter and Cell Viability Assay kit and associated reagents were purchased from Promega (Madison, WI).

Intraparenchymal LNP injections

In an induction chamber, mice were anesthetized with 5% inhaled isoflurane in oxygen (1 L/min) until the righting reflex was lost and the breathing pattern slowed and deepened. Anesthetized mice were immediately transferred to a stereotaxic apparatus equipped with an isoflurane vapourizer and a 37°C heating pad. Mice were maintained in a surgical plane of anesthesia with 3%–4% isoflurane in oxygen (1 L/min) throughout surgery. The surgical plane was routinely checked through toe pinch and blink reflexes. A sterile ophthalmic lubricant was applied to the eyes to prevent corneal drying, and a lubricated rectal probe was placed into the rectum to monitor temperature. Warmed 0.9% sodium chloride (w/v; 20 mL/kg) and Metacam (5 mg/kg) solutions were administered subcutaneously for supportive care and prophylactic analgesia, respectively. Hair was removed from the surgery site using an electric clipper, and the area was cleaned three times with a skin soap solution comprised of 4% chlorhexidine gluconate followed by 70% isopropanol (v/v). Bupivacaine (5 mg/kg) was administered to provide analgesia by infiltrating under the skin at the injection site after the first skin cleaning preparation. A small vertical incision in the skin of the head was made to expose the skull, and a small hole was drilled in the bone at predetermined coordinates to reach the striatum (+0.8 mm anterior-posterior, ±1.8 mm medial-lateral, and 3.5 mm dorsal-ventral from Bregma).[67] 0.5 μL of PBS (control) or LNP (treatment) containing siRNA or mRNA was bilaterally injected through burr holes using a 10 μL Hamilton syringe coupled to a 32-gauge glass microneedle at a rate of 250 nL/min. The microneedle was left in position for two minutes before initiating the injection, and left in place for five minutes after the injection was completed to allow diffusion of the solution. The needle was slowly removed from the brain, and a surgical drape with a fenestration for the head was placed on top of the animal to maintain sterility. Skin incisions were sutured using a monofilament suture (Monocryl 5-0). Animals were maintained on a 37°C heating pad and observed until fully recovered from anesthesia. Post-recovery, animals were housed individually with Hydrogel and food freely available on the bottom of cages, as well as regular food in the hopper and water *ad libitum*. Following the procedure, a surgery card was marked to indicate animal ID, procedures performed, drugs administered, and all health checks performed. Mice were monitored every 4–8 h during the first 24 h after surgery, and then twice per day for at least 48 h following surgery or until the mice were healthy and the incisions healed. Body weight, incision sites, and general health were assessed and recorded on dedicated monitoring sheets. Metacam (5 mg/kg) was subcutaneously administered daily for two days, and 0.9% sodium chloride (w/v; 20 mL/kg) was administered to prevent dehydration.

Brain microdissection

Four hours (mRNA) or seven days (siRNA) after injection, mice were injected intraperitoneally with a lethal dose of Avertin (750 mg/kg; 2,2,2-tribromoethanol; Millipore Sigma, Burlington, MA) and returned to their cages until deep anesthesia was reached. Anesthetized mice were immediately transferred to an induction chamber and euthanized with CO_2 at a flow rate of 6 L/min, followed by cervical dislocation. Brains were harvested and microdissected on ice to isolate cortex, hippocampus, striatum, and cerebellum tissues. Brain region- and hemisphere-separated tissues were collected in 1.7 mL Eppendorf tubes and snap-frozen on dry ice. Samples were stored at −80°C for downstream analysis.

Brain tissue homogenization

For tissues collected from LNP siRNA-treated mice, microdissected brain regions were placed in chilled Lysing Matrix D tubes (MP Biomedicals, Santa Ana, CA) and homogenized in 600 μL of RNA lysis buffer (Invitrogen) containing 1% 2-mercaptoethanol using a

FastPrep FP120 cell disruptor (MP Biomedicals, Santa Ana, CA). Homogenates were combined with an equal volume of 70% RNase-free ethanol (v/v) and RNA extraction was performed using a PureLink RNA Mini kit (Invitrogen) as described above according to the manufacturer's instructions. Quantitative RT-PCR was performed as described above. For tissues collected from LNP mRNA-treated mice, individual microdissected brain regions were combined with 75 μL of chilled 1X Glo Lysis Buffer (Promega) and manually Dounce homogenized. Samples were spun at maximum speed (>13,000 RPM) for five minutes to pellet the tissue, and 50 μL of tissue lysate was transferred to a 96-well plate (Corning). Samples were combined with an equal volume of SteadyGlo luciferase reagent (Promega), mixed on an orbital shaker for 30 s, and incubated on the benchtop for five minutes. Luminescence was measured using a POLARStar Omega microplate luminometer (BMG Labtech).

Immunohistochemistry

Four hours after injection, mice were anesthetized by intraperitoneal injection with a lethal dose of Avertin (750 mg/kg; 2,2,2-tribromoethanol; Millipore Sigma, Burlington, MA) and terminally perfused through the ascending aorta with cold 3% paraformaldehyde in 0.1M phosphate buffer at a flow rate of 5 mL/min for 10 min. Brains were post-fixed at 4°C in 3% paraformaldehyde overnight, transferred to 1X PBS, and stored at 4°C for downstream processing. Before immunostaining, brains were equilibrated in 30% sucrose in 1X PBS for 24–48 h at 4°C, embedded in O.C.T. compound (Fisher Scientific, Pittsburgh, PA, USA) and frozen on dry ice. 25 μm coronal sections were cut through the entire striatum using a CM3050 S cryostat (Leica), serially collected in 1X PBS +0.01% sodium azide, and stored at 4°C. Serial coronal sections spaced 150 μm apart were permeabilized in PBS-T (1X PBS + 0.1% Triton X-100), blocked at room temperature (RT) in PBS-T + 10% normal donkey serum (NDS) and incubated at 4°C for 12–20 h in PBS-T + 2% NDS with the following primary antibodies: firefly luciferase (Luc; Abcam Cat.# ab181640; 1:300), glial fibrillary acidic protein (GFAP; Sigma Cat.#G3893; 1:500), calcium adapter binding protein (Iba1; Invitrogen Cat.# PA5-21274; 1:200), neuronal nuclei (NeuN; Abcam Cat.# ab177487; 1:250) and glutathione S-transferase pi (GST-P; MBL Life Science Cat.# 311; 1:200). Immunofluorescent detection was achieved by incubation with appropriate species-specific Alexa Fluor 488 or Alexa Fluor 594 secondary antibodies (Invitrogen; 1:1,000) in PBS-T + 2% NDS at RT. Immunostained sections were incubated for five minutes at RT in 1X PBS + 4′,6-diamidino-2-phenylindole (DAPI; Millipore Sigma; 1:50,000), mounted on SuperFrost slides (Fisher Scientific, Pittsburgh, PA, USA), and coverslipped using ProLong Gold Antifade Mountant (Fisher Scientific, Pittsburgh, PA, USA). Images were captured using an Olympus BX61 Fluorescence and Transmittance Wide Field Microscope (Olympus Life Sciences, Tokyo, Japan). Cells were counted manually using the Fiji Cell Counter plugin (ImageJ2 v2.16.0/1.54p).

Statistics

All statistical analyses were performed using Prism v.9 software (GraphPad). Data are presented as mean (SD). p values of less than 0.05 were considered significant. The effect of ApoE supplementation on LNP uptake was assessed by unpaired t test. Formulation strategies were compared using one-way analysis of variance (ANOVA) with formulation method or mRNA dose as factors and Bonferroni post-hoc analysis to correct for multiple comparisons. siRNA cationic lipid and phospholipid screens were analyzed using one-way ANOVA with Dunnett and Tukey post-hoc analyses to respectively compare treatment means to control means, and to compare individual means, and to correct for multiple comparisons. siRNA viability measurements and mRNA ionizable cationic lipid and phospholipid screens and viability measurements were assessed using two-way ANOVA with Dunnett or Tukey post-hoc analyses to respectively compare treatment means with control means and to compare individual means, and to correct for multiple comparisons. LNP composition and siRNA or mRNA dose were examined as factors. Linear regression associations between datasets were assessed using Pearson correlation.

DATA AND CODE AVAILABILITY

The data that support the findings of this study are available from the corresponding author upon reasonable request.

ACKNOWLEDGMENTS

This work was funded by a NanoMedicines Innovation Network (NMIN) Project Grant (#2019-T2-03). S.B.T. was supported by an NMIN Doctoral Graduate Award and a Huntington Society of Canada Doctoral Graduate Award. J.A.K. was supported by an NMIN Postdoctoral Fellowship Award in Gene Therapy. The authors would like to thank Dr. Dominik Witzigmann for helpful discussions regarding experimental design.

AUTHOR CONTRIBUTIONS

S.B.T., J.A.K., T.L.P., and B.R.L. contributed to project conceptualization. S.B.T. performed all formal data analysis. S.B.T., J.A.K., T.L.P., and B.R.L. acquired the funding that supported this study. S.B.T. performed all primary neuron cultures, quantitative RT-PCR, luciferase assays and microscopy, and contributed to immunohistochemistry and image analysis. P.K. contributed to immunohistochemistry and image analysis. A.d.M.G. and T.L.P. performed *in vivo* intrastriatal LNP injections and brain microdissections. A.d.M.G. prepared brains for immunohistochemical analysis. D.Z.K. and J.A.K. prepared LNP formulations. F.S. and M.A.C. synthesized the ionizable cationic lipid MF019. P.K.W. provided technical support for primary neuron culture protocols. S.B.T. designed methodologies with input from J.A.K., T.L.P., and B.R.L. S.B.T., T.L.P., and B.R.L. contributed to project administration. P.R.C. and B.R.L. provided the resources and supervision required for this study. S.B.T. performed all data visualization, prepared the original draft of the manuscript, and reviewed and edited the submitted manuscript. All authors reviewed and edited the manuscript prior to submission.

DECLARATION OF INTERESTS

P.K.W. and B.R.L. are co-founders of Incisive Genetics, Inc. J.A.K., M.A.C., and P.R.C. are co-founders of NanoVation Therapeutics, Inc. D.Z.K., J.A.K., F.S., and M.A.C. are employees of NanoVation Therapeutics, Inc. T.L.P. is an employee of Polymorphic BioSciences, Inc. P.R.C. is the founder of Polymorphic BioSciences, Inc. J.A.K., T.L.P., P.K.W., P.R.C., and B.R.L. are among inventors of the patent entitled "Compositions and Systems Comprising Transfection-Competent Vesicles Free of Organic-Solvents and Detergents and Methods Related Thereto" (WO/2020/077007), which is related to this work.

SUPPLEMENTAL INFORMATION

Supplemental information can be found online at https://doi.org/10.1016/j.omtn.2026.103025.

REFERENCES

1. Feigin, V.L., Nichols, E., Alam, T., Bannick, M.S., Beghi, E., Blake, N., Culpepper, W.J., Dorsey, E.R., Elbaz, A., Ellenbogen, R.G., et al. (2019). Global, regional, and

national burden of neurological disorders, 1990-2016: a systematic analysis for the Global Burden of Disease Study 2016. Lancet Neurol. *18*, 459–480. https://doi.org/10.1016/S1474-4422(18)30499-X.

2. MacDonald, M.E., Ambrose, C.M., Duyao, M.P., Myers, R.H., Lin, C., Srinidhi, L., Barnes, G., Taylor, S.A., James, M., and Groot, N. (1993). A novel gene containing a trinucleotide repeat that is expanded and unstable on Huntington's disease chromosomes. Cell *72*, 971–983.
3. Blauwendraat, C., Nalls, M.A., and Singleton, A.B. (2020). The genetic architecture of Parkinson's disease. Lancet Neurol. *19*, 170–178. https://doi.org/10.1016/S1474-4422(19)30287-X.
4. Kunkle, B.W., Grenier-Boley, B., Sims, R., Bis, J.C., Damotte, V., Naj, A.C., Boland, A., Vronskaya, M., van der Lee, S.J., Amlie-Wolf, A., et al. (2019). Genetic meta-analysis of diagnosed Alzheimer's disease identifies new risk loci and implicates Abeta, tau, immunity and lipid processing. Nat. Genet. *51*, 414–430. https://doi.org/10.1038/s41588-019-0358-2.
5. Krichevsky, A.M., and Kosik, K.S. (2002). RNAi functions in cultured mammalian neurons. Proc. Natl. Acad. Sci. USA *99*, 11926–11929. https://doi.org/10.1073/pnas.182272699.
6. Bisgaier, C.L., Siebenkas, M.V., and Williams, K.J. (1989). Effects of apolipoproteins A-IV and A-I on the uptake of phospholipid liposomes by hepatocytes. J. Biol. Chem. *264*, 862–866.
7. Akinc, A., Querbes, W., De, S., Qin, J., Frank-Kamenetsky, M., Jayaprakash, K.N., Jayaraman, M., Rajeev, K.G., Cantley, W.L., Dorkin, J.R., et al. (2010). Targeted delivery of RNAi therapeutics with endogenous and exogenous ligand-based mechanisms. Mol. Ther. *18*, 1357–1364. https://doi.org/10.1038/mt.2010.85.
8. Elliott, D.A., Weickert, C.S., and Garner, B. (2010). Apolipoproteins in the brain: implications for neurological and psychiatric disorders. Clin. Lipidol. *51*, 555–573. https://doi.org/10.2217/CLP.10.37.
9. Herz, J. (2009). Apolipoprotein E receptors in the nervous system. Curr. Opin. Lipidol. *20*, 190–196. https://doi.org/10.1097/MOL.0b013e32832d3a10.
10. Tanaka, H., Nakatani, T., Furihata, T., Tange, K., Nakai, Y., Yoshioka, H., Harashima, H., and Akita, H. (2018). In Vivo Introduction of mRNA Encapsulated in Lipid Nanoparticles to Brain Neuronal Cells and Astrocytes via Intracerebroventricular Administration. Mol. Pharm. *15*, 2060–2067. https://doi.org/10.1021/acs.molpharmaceut.7b01084.
11. Rungta, R.L., Choi, H.B., Lin, P.J., Ko, R.W., Ashby, D., Nair, J., Manoharan, M., Cullis, P.R., and Macvicar, B.A. (2013). Lipid Nanoparticle Delivery of siRNA to Silence Neuronal Gene Expression in the Brain. Mol. Ther. Nucleic Acids *2*, e136. https://doi.org/10.1038/mtna.2013.65.
12. Kim, H.J., Seo, S.W., Chang, J.W., Lee, J.I., Kim, C.H., Chin, J., Choi, S.J., Kwon, H., Yun, H.J., Lee, J.M., et al. (2015). Stereotactic brain injection of human umbilical cord blood mesenchymal stem cells in patients with Alzheimer's disease dementia: A phase 1 clinical trial. Alzheimer's Dement. *1*, 95–102. https://doi.org/10.1016/j.trci.2015.06.007.
13. Anderson, E.J., Rouphael, N.G., Widge, A.T., Jackson, L.A., Roberts, P.C., Makhene, M., Chappell, J.D., Denison, M.R., Stevens, L.J., Pruijssers, A.J., et al. (2020). Safety and Immunogenicity of SARS-CoV-2 mRNA-1273 Vaccine in Older Adults. N. Engl. J. Med. *383*, 2427–2438. https://doi.org/10.1056/NEJMoa2028436.
14. Sahin, U., Muik, A., Derhovanessian, E., Vogler, I., Kranz, L.M., Vormehr, M., Baum, A., Pascal, K., Quandt, J., Maurus, D., et al. (2020). COVID-19 vaccine BNT162b1 elicits human antibody and T(H)1 T cell responses. Nature *586*, 594–599. https://doi.org/10.1038/s41586-020-2814-7.
15. Jayaraman, M., Ansell, S.M., Mui, B.L., Tam, Y.K., Chen, J., Du, X., Butler, D., Eltepu, L., Matsuda, S., Narayanannair, J.K., et al. (2012). Maximizing the potency of siRNA lipid nanoparticles for hepatic gene silencing in vivo. Angew. Chem. Int. Ed. Engl. *51*, 8529–8533. https://doi.org/10.1002/anie.201203263.
16. Kulkarni, J.A., Myhre, J.L., Chen, S., Tam, Y.Y.C., Danescu, A., Richman, J.M., and Cullis, P.R. (2017). Design of lipid nanoparticles for in vitro and in vivo delivery of plasmid DNA. Nanomedicine. *13*, 1377–1387. https://doi.org/10.1016/j.nano.2016.12.014.
17. Akita, H., Nakatani, T., Kuroki, K., Maenaka, K., Tange, K., Nakai, Y., and Harashima, H. (2015). Effect of hydrophobic scaffold on the cellular uptake and gene transfection activities of DNA-encapsulating liposomal nanoparticles via intracerebroventricular administration. Int. J. Pharm. *490*, 142–145. https://doi.org/10.1016/j.ijpharm.2015.05.043.
18. Okabe, M., Ikawa, M., Kominami, K., Nakanishi, T., and Nishimune, Y. (1997). 'Green mice' as a source of ubiquitous green cells. FEBS Lett. *407*, 313–319. https://doi.org/10.1016/s0014-5793(97)00313-x.
19. Leung, A.K.K., Tam, Y.Y.C., Chen, S., Hafez, I.M., and Cullis, P.R. (2015). Microfluidic Mixing: A General Method for Encapsulating Macromolecules in Lipid Nanoparticle Systems. J. Phys. Chem. B *119*, 8698–8706. https://doi.org/10.1021/acs.jpcb.5b02891.
20. Jeffs, L.B., Palmer, L.R., Ambegia, E.G., Giesbrecht, C., Ewanick, S., and MacLachlan, I. (2005). A scalable, extrusion-free method for efficient liposomal encapsulation of plasmid DNA. Pharm. Res. *22*, 362–372. https://doi.org/10.1007/s11095-004-1873-z.
21. Kulkarni, J.A., Thomson, S.B., Zaifman, J., Leung, J., Wagner, P.K., Hill, A., Tam, Y.Y.C., Cullis, P.R., Petkau, T.L., and Leavitt, B.R. (2020). Spontaneous, solvent-free entrapment of siRNA within lipid nanoparticles. Nanoscale *12*, 23959–23966. https://doi.org/10.1039/d0nr06816k.
22. Mirjalili Mohanna, S.Z., Djaksigulova, D., Hill, A.M., Wagner, P.K., Simpson, E.M., and Leavitt, B.R. (2022). LNP-mediated delivery of CRISPR RNP for wide-spread in vivo genome editing in mouse cornea. J. Control. Release *350*, 401–413. https://doi.org/10.1016/j.jconrel.2022.08.042.
23. Adair, B.A., Korecki, A.J., Djaksigulova, D., Wagner, P.K., Chiu, N.Y., Lam, S.L., Lengyell, T.C., Leavitt, B.R., and Simpson, E.M. (2023). ABE8e Corrects Pax6-Aniridic Variant in Humanized Mouse ESCs and via LNPs in Ex Vivo Cortical Neurons. Ophthalmol. Ther. *12*, 2049–2068. https://doi.org/10.1007/s40123-023-00729-6.
24. Schlich, M., Palomba, R., Costabile, G., Mizrahy, S., Pannuzzo, M., Peer, D., and Decuzzi, P. (2021). Cytosolic delivery of nucleic acids: The case of ionizable lipid nanoparticles. Bioeng. Transl. Med. *6*, e10213. https://doi.org/10.1002/btm2.10213.
25. Akinc, A., Maier, M.A., Manoharan, M., Fitzgerald, K., Jayaraman, M., Barros, S., Ansell, S., Du, X., Hope, M.J., Madden, T.D., et al. (2019). The Onpattro story and the clinical translation of nanomedicines containing nucleic acid-based drugs. Nat. Nanotechnol. *14*, 1084–1087. https://doi.org/10.1038/s41565-019-0591-y.
26. Ciufolini, M., and Ferguson, M.L. (2022). Sulfur-containing lipids. patent application PCT/CA2022/050042.
27. Karra, D., and Dahm, R. (2010). Transfection techniques for neuronal cells. J. Neurosci. *30*, 6171–6177. https://doi.org/10.1523/JNEUROSCI.0183-10.2010.
28. Kauffman, K.J., Dorkin, J.R., Yang, J.H., Heartlein, M.W., DeRosa, F., Mir, F.F., Fenton, O.S., and Anderson, D.G. (2015). Optimization of Lipid Nanoparticle Formulations for mRNA Delivery in Vivo with Fractional Factorial and Definitive Screening Designs. Nano Lett. *15*, 7300–7306. https://doi.org/10.1021/acs.nanolett.5b02497.
29. Paunovska, K., Sago, C.D., Monaco, C.M., Hudson, W.H., Castro, M.G., Rudoltz, T.G., Kalathoor, S., Vanover, D.A., Santangelo, P.J., Ahmed, R., et al. (2018). A Direct Comparison of in Vitro and in Vivo Nucleic Acid Delivery Mediated by Hundreds of Nanoparticles Reveals a Weak Correlation. Nano Lett. *18*, 2148–2157. https://doi.org/10.1021/acs.nanolett.8b00432.
30. Vonsattel, J.P., Myers, R.H., Stevens, T.J., Ferrante, R.J., Bird, E.D., and Richardson, E.P., Jr. (1985). Neuropathological classification of Huntington's disease. J. Neuropathol. Exp. Neurol. *44*, 559–577. https://doi.org/10.1097/00005072-198511000-00003.
31. Novelli, M., Galosi, S., Zorzi, G., Martinelli, S., Capuano, A., Nardecchia, F., Granata, T., Pollini, L., Di Rocco, M., Marras, C.E., et al. (2023). GNAO1-related movement disorder: An update on phenomenology, clinical course, and response to treatments. Parkinsonism Relat. Disord. *111*, 105405. https://doi.org/10.1016/j.parkreldis.2023.105405.
32. Karikó, K., Muramatsu, H., Welsh, F.A., Ludwig, J., Kato, H., Akira, S., and Weissman, D. (2008). Incorporation of pseudouridine into mRNA yields superior nonimmunogenic vector with increased translational capacity and biological stability. Mol. Ther. *16*, 1833–1840. https://doi.org/10.1038/mt.2008.200.
33. Evers, M.J.W., Kulkarni, J.A., van der Meel, R., Cullis, P.R., Vader, P., and Schiffelers, R.M. (2018). State-of-the-Art Design and Rapid-Mixing Production Techniques of Lipid Nanoparticles for Nucleic Acid Delivery. Small Methods *2*, 1700375. https://doi.org/10.1002/smtd.201700375.

34. Bouraoui, A., Berchel, M., Ghanem, R., Vié, V., Paboeuf, G., Deschamps, L., Lozach, O., Le Gall, T., Montier, T., and Jaffrès, P.A. (2019). Substitution of unsaturated lipid chains by thioether-containing lipid chains in cationic amphiphiles: physicochemical consequences and application for gene delivery. Org. Biomol. Chem. *17*, 3609–3616. https://doi.org/10.1039/c9ob00473d.

35. Lonez, C., Vandenbranden, M., and Ruysschaert, J.M. (2012). Cationic lipids activate intracellular signaling pathways. Adv. Drug Deliv. Rev. *64*, 1749–1758. https://doi.org/10.1016/j.addr.2012.05.009.

36. He, Y., Bi, D., Plantinga, J.A., Molema, G., Bussmann, J., and Kamps, J.A.A.M. (2022). Development of a Combined Lipid-Based Nanoparticle Formulation for Enhanced siRNA Delivery to Vascular Endothelial Cells. Pharmaceutics *14*, 2086. https://doi.org/10.3390/pharmaceutics14102086.

37. Suri, K., Pfeifer, L., Cvet, D., Li, A., McCoy, M., Singh, A., and Amiji, M.M. (2025). Oral delivery of stabilized lipid nanoparticles for nucleic acid therapeutics. Drug Deliv. Transl. Res. *15*, 1755–1769. https://doi.org/10.1007/s13346-024-01709-4.

38. Lv, H., Zhang, S., Wang, B., Cui, S., and Yan, J. (2006). Toxicity of cationic lipids and cationic polymers in gene delivery. J. Control. Release *114*, 100–109. https://doi.org/10.1016/j.jconrel.2006.04.014.

39. Carrasco, M.J., Alishetty, S., Alameh, M.G., Said, H., Wright, L., Paige, M., Soliman, O., Weissman, D., Cleveland, T.E., 4th, Grishaev, A., and Buschmann, M.D. (2021). Ionization and structural properties of mRNA lipid nanoparticles influence expression in intramuscular and intravascular administration. Commun. Biol. *4*, 956. https://doi.org/10.1038/s42003-021-02441-2.

40. Heyes, J., Palmer, L., Bremner, K., and MacLachlan, I. (2005). Cationic lipid saturation influences intracellular delivery of encapsulated nucleic acids. J. Control. Release *107*, 276–287. https://doi.org/10.1016/j.jconrel.2005.06.014.

41. Cullis, P.R., Hope, M.J., and Tilcock, C.P. (1986). Lipid polymorphism and the roles of lipids in membranes. Chem. Phys. Lipids *40*, 127–144. https://doi.org/10.1016/0009-3084(86)90067-8.

42. Farhood, H., Serbina, N., and Huang, L. (1995). The role of dioleoyl phosphatidylethanolamine in cationic liposome mediated gene transfer. Biochim. Biophys. Acta *1235*, 289–295. https://doi.org/10.1016/0005-2736(95)80016-9.

43. Wan, C., Allen, T.M., and Cullis, P.R. (2014). Lipid nanoparticle delivery systems for siRNA-based therapeutics. Drug Deliv. Transl. Res. *4*, 74–83. https://doi.org/10.1007/s13346-013-0161-z.

44. Hafez, I.M., Maurer, N., and Cullis, P.R. (2001). On the mechanism whereby cationic lipids promote intracellular delivery of polynucleic acids. Gene Ther. *8*, 1188–1196. https://doi.org/10.1038/sj.gt.3301506.

45. Cullis, P.R., and de Kruijff, B. (1979). Lipid polymorphism and the functional roles of lipids in biological membranes. Biochim. Biophys. Acta *559*, 399–420. https://doi.org/10.1016/0304-4157(79)90012-1.

46. Kenny, G.D., Bienemann, A.S., Tagalakis, A.D., Pugh, J.A., Welser, K., Campbell, F., Tabor, A.B., Hailes, H.C., Gill, S.S., Lythgoe, M.F., et al. (2013). Multifunctional receptor-targeted nanocomplexes for the delivery of therapeutic nucleic acids to the brain. Biomaterials *34*, 9190–9200. https://doi.org/10.1016/j.biomaterials.2013.07.081.

47. Tagalakis, A.D., Kenny, G.D., Bienemann, A.S., McCarthy, D., Munye, M.M., Taylor, H., Wyatt, M.J., Lythgoe, M.F., White, E.A., and Hart, S.L. (2014). PEGylation improves the receptor-mediated transfection efficiency of peptide-targeted, self-assembling, anionic nanocomplexes. J. Control. Release *174*, 177–187. https://doi.org/10.1016/j.jconrel.2013.11.014.

48. Tagalakis, A.D., Lee, D.H.D., Bienemann, A.S., Zhou, H., Munye, M.M., Saraiva, L., McCarthy, D., Du, Z., Vink, C.A., Maeshima, R., et al. (2014). Multifunctional, self-assembling anionic peptide-lipid nanocomplexes for targeted siRNA delivery. Biomaterials *35*, 8406–8415. https://doi.org/10.1016/j.biomaterials.2014.06.003.

49. Song, L.Y., Ahkong, Q.F., Rong, Q., Wang, Z., Ansell, S., Hope, M.J., and Mui, B. (2002). Characterization of the inhibitory effect of PEG-lipid conjugates on the intracellular delivery of plasmid and antisense DNA mediated by cationic lipid liposomes. Biochim. Biophys. Acta *1558*, 1–13. https://doi.org/10.1016/s0005-2736(01)00399-6.

50. Jackson, A.L., and Linsley, P.S. (2010). Recognizing and avoiding siRNA off-target effects for target identification and therapeutic application. Nat. Rev. Drug Discov. *9*, 57–67. https://doi.org/10.1038/nrd3010.

51. Puttfarcken, P.S., Manelli, A.M., Falduto, M.T., Getz, G.S., and LaDu, M.J. (1997). Effect of apolipoprotein E on neurite outgrowth and beta-amyloid-induced toxicity in developing rat primary hippocampal cultures. J. Neurochem. *68*, 760–769. https://doi.org/10.1046/j.1471-4159.1997.68020760.x.

52. Nathan, B.P., Jiang, Y., Wong, G.K., Shen, F., Brewer, G.J., and Struble, R.G. (2002). Apolipoprotein E4 inhibits, and apolipoprotein E3 promotes neurite outgrowth in cultured adult mouse cortical neurons through the low-density lipoprotein receptor-related protein. Brain Res. *928*, 96–105. https://doi.org/10.1016/s0006-8993(01)03367-4.

53. Michikawa, M., and Yanagisawa, K. (1999). Inhibition of cholesterol production but not of nonsterol isoprenoid products induces neuronal cell death. J. Neurochem. *72*, 2278–2285. https://doi.org/10.1046/j.1471-4159.1999.0722278.x.

54. Isaacson, J.S., and Scanziani, M. (2011). How inhibition shapes cortical activity. Neuron *72*, 231–243. https://doi.org/10.1016/j.neuron.2011.09.027.

55. Tepper, J.M., Tecuapetla, F., Koós, T., and Ibáñez-Sandoval, O. (2010). Heterogeneity and diversity of striatal GABAergic interneurons. Front. Neuroanat. *4*, 150. https://doi.org/10.3389/fnana.2010.00150.

56. Yue, F., Cheng, Y., Breschi, A., Vierstra, J., Wu, W., Ryba, T., Sandstrom, R., Ma, Z., Davis, C., Pope, B.D., et al. (2014). A comparative encyclopedia of DNA elements in the mouse genome. Nature *515*, 355–364. https://doi.org/10.1038/nature13992.

57. Witzigmann, D., Kulkarni, J.A., Leung, J., Chen, S., Cullis, P.R., and van der Meel, R. (2020). Lipid nanoparticle technology for therapeutic gene regulation in the liver. Adv. Drug Deliv. Rev. *159*, 344–363. https://doi.org/10.1016/j.addr.2020.06.026.

58. Dislich, B., Wohlrab, F., Bachhuber, T., Müller, S.A., Kuhn, P.H., Hogl, S., Meyer-Luehmann, M., and Lichtenthaler, S.F. (2015). Label-free Quantitative Proteomics of Mouse Cerebrospinal Fluid Detects beta-Site APP Cleaving Enzyme (BACE1) Protease Substrates In Vivo. Mol. Cell. Proteomics *14*, 2550–2563. https://doi.org/10.1074/mcp.M114.041533.

59. Lacar, B., Young, S.Z., Platel, J.C., and Bordey, A. (2010). Imaging and recording subventricular zone progenitor cells in live tissue of postnatal mice. Front. Neurosci. *4*, 43. https://doi.org/10.3389/fnins.2010.00043.

60. Jablonska, B., Aguirre, A., Raymond, M., Szabo, G., Kitabatake, Y., Sailor, K.A., Ming, G.L., Song, H., and Gallo, V. (2010). Chordin-induced lineage plasticity of adult SVZ neuroblasts after demyelination. Nat. Neurosci. *13*, 541–550. https://doi.org/10.1038/nn.2536.

61. Deng, S., Gan, L., Liu, C., Xu, T., Zhou, S., Guo, Y., Zhang, Z., Yang, G.Y., Tian, H., and Tang, Y. (2023). Roles of Ependymal Cells in the Physiology and Pathology of the Central Nervous System. Aging Dis. *14*, 468–483. https://doi.org/10.14336/AD.2022.0826-1.

62. Sawamura, M., Tachikawa, K., Hikawa, R., Akiyama, H., Kaji, S., Yasuda, K., Leu, A.I., Hong, H., Mukthavaram, R., Chivukula, P., et al. (2024). A lipid nanoparticle-based oligodendrocyte-specific mRNA therapy. Mol. Ther. Nucleic Acids *35*, 102380. https://doi.org/10.1016/j.omtn.2024.102380.

63. Akinc, A., Zumbuehl, A., Goldberg, M., Leshchiner, E.S., Busini, V., Hossain, N., Bacallado, S.A., Nguyen, D.N., Fuller, J., Alvarez, R., et al. (2008). A combinatorial library of lipid-like materials for delivery of RNAi therapeutics. Nat. Biotechnol. *26*, 561–569. https://doi.org/10.1038/nbt1402.

64. da Cruz, M.T.G., Simões, S., and de Lima, M.C.P. (2004). Improving lipoplex-mediated gene transfer into C6 glioma cells and primary neurons. Exp. Neurol. *187*, 65–75. https://doi.org/10.1016/j.expneurol.2003.12.013.

65. Kulkarni, J.A., Darjuan, M.M., Mercer, J.E., Chen, S., van der Meel, R., Thewalt, J.L., Tam, Y.Y.C., and Cullis, P.R. (2018). On the Formation and Morphology of Lipid Nanoparticles Containing Ionizable Cationic Lipids and siRNA. ACS Nano *12*, 4787–4795. https://doi.org/10.1021/acsnano.8b01516.

66. Vandesompele, J., De Preter, K., Pattyn, F., Poppe, B., Van Roy, N., De Paepe, A., and Speleman, F. (2002). Accurate normalization of real-time quantitative RT-PCR data by geometric averaging of multiple internal control genes. Genome Biol. *3*, RESEARCH0034. https://doi.org/10.1186/gb-2002-3-7-research0034.

67. Paxinos, G., and Franklin, K.B. (2019). Paxinos and Franklin's the Mouse Brain in Stereotaxic Coordinates (Academic press).

OMTN, Volume *37*

Supplemental information

Design and development of lipid nanoparticle formulations for brain gene therapy

Sarah B. Thomson, Alissandra de Moura Gomes, Pardis Kazemian, Daniel Z. Kurek, Jayesh A. Kulkarni, Fariba Sadaati, Pamela K. Wagner, Terri L. Petkau, Marco A. Ciufolini, Pieter R. Cullis, and Blair R. Leavitt

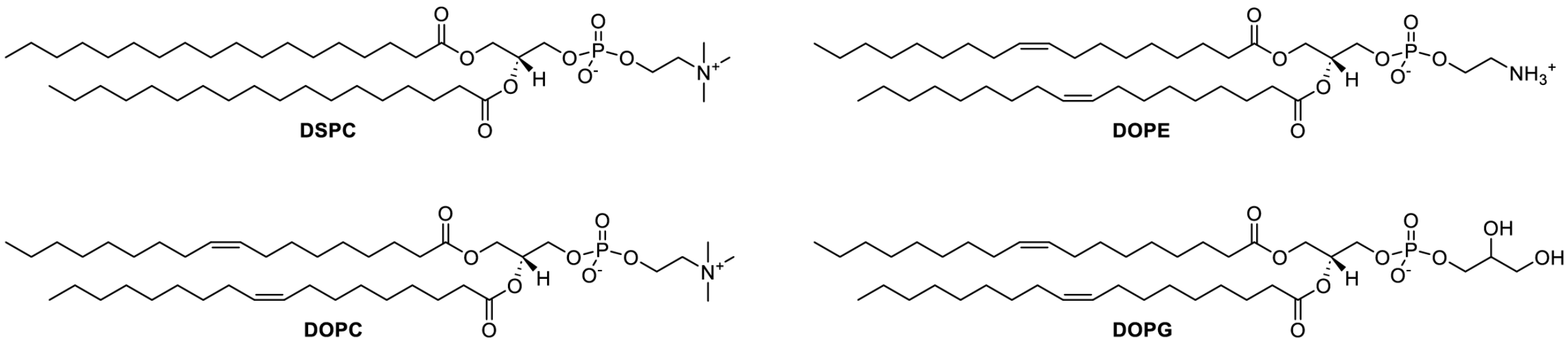

Figure S1. Structures of the phospholipids used for LNP formulation screening.

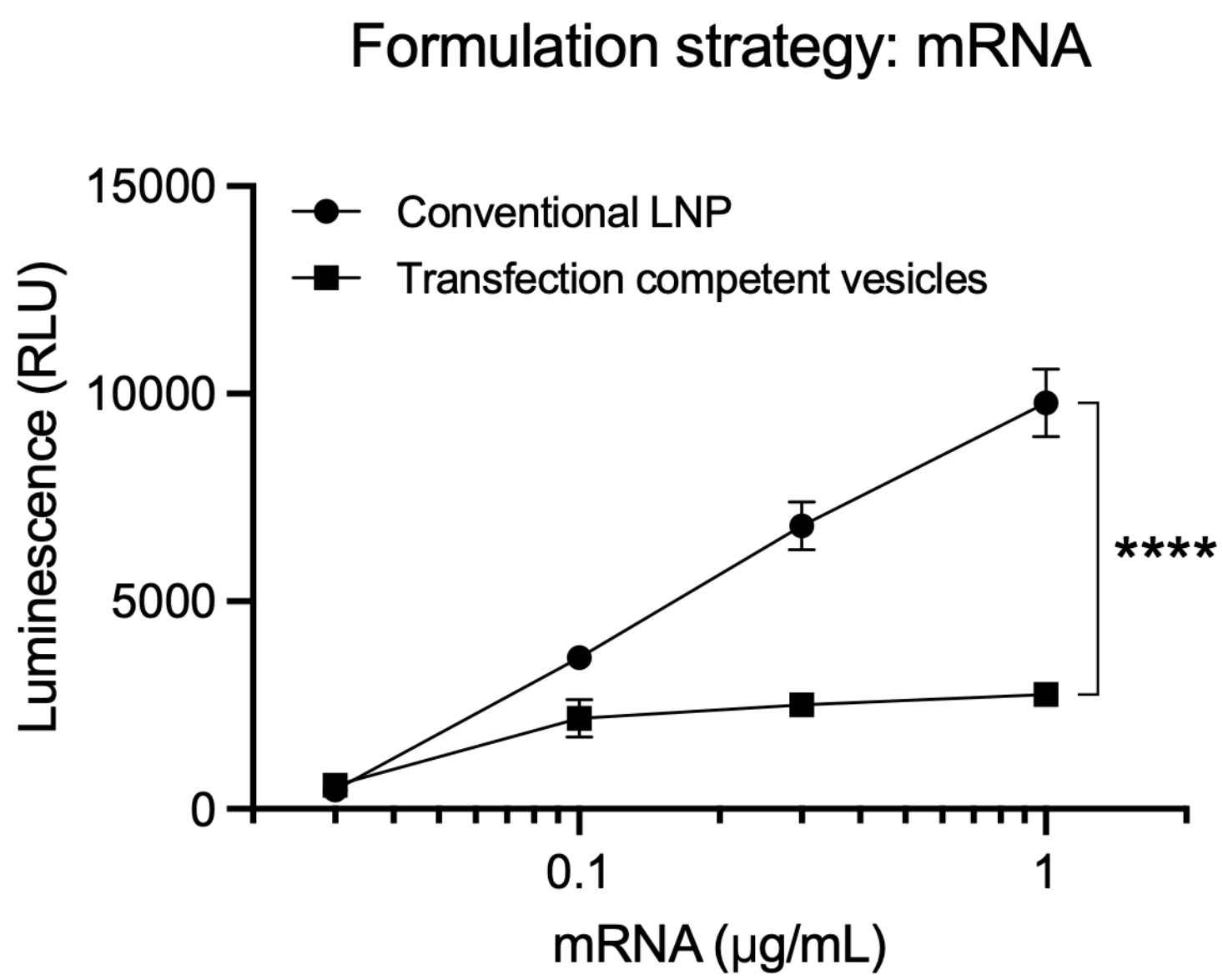

Figure S2. Rapid mixing and benchtop mixing strategies for LNP loading with FLuc mRNA were evaluated. LNPs prepared using rapid mixing had improved activity in primary neurons compared to LNPs loaded using benchtop mixing (2-way ANOVA, Loading method: $F_{(1, 24)} = 472.1$, $p<0.0001$). N=3-4 biological replicates (N=1 technical replicate) per condition. mRNA treatments were performed at 0.03-1.0 µg/mL FLuc mRNA. Data represent the mean (SD).

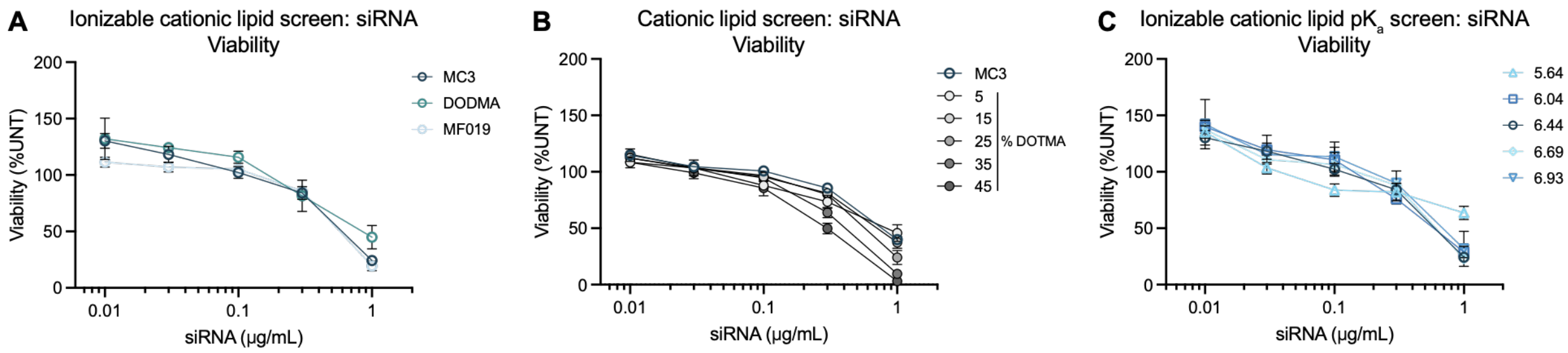

Figure S3. siRNA-containing LNPs prepared with distinct ionizable cationic lipid species differentially affect primary neuron viability. (A) Neuronal viability was impacted by ionizable cationic lipid species and siRNA dose (2-way ANOVA, Composition: $F_{(2,45)} = 16.5$, p<0.0001; Dose: $F_{(4,45)} = 296.2$, p<0.0001). (B) Increasing the DOTMA content of MC3 LNPs progressively worsened neuronal viability (2-way ANOVA, Composition: $F_{(4,75)} = 45.59$, p<0.0001). (C) Ionizable cationic lipid apparent pK$_a$ did not differentially affect neuronal viability (2-way ANOVA, pK$_a$: $F_{(4,75)} = 1.551$, p=0.1962). The viability of treated neurons is normalized to the viability measured in untreated primary neurons. N=4 biological replicates (N=1 technical replicate) per condition. siRNA treatments were performed at 0.01-1.0 µg/mL GFP siRNA. Data represent the mean (SD).

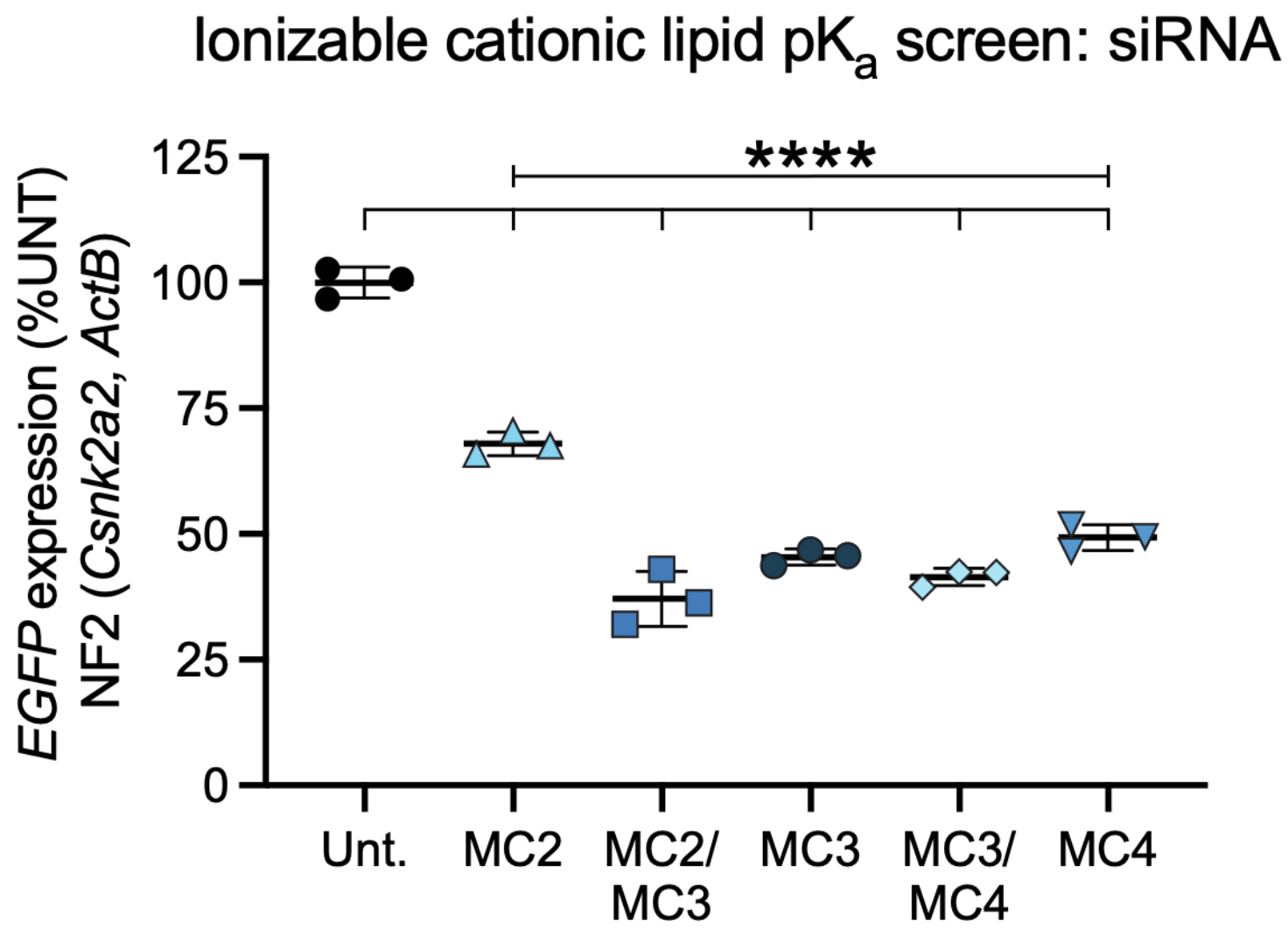

Figure S4. Ionizable cationic lipid apparent pK$_a$ variably impacts the potency of siRNA-containing LNPs in primary neurons. siRNA-containing LNPs prepared using ionizable cationic lipids with a range of apparent pK$_a$s (MC2=5.64; MC2/MC3=6.04; MC3=6.44); MC3/MC4=6.69; MC4=6.93) and containing GFP siRNA knock down *EGFP* in primary neurons with variable efficacy. *EGFP* expression in treated neurons is normalized to the level of *EGFP* expression measured in untreated primary neurons. N=3 biological replicates (N=2 technical replicates) per condition. siRNA treatments were performed at 0.01 µg/mL GFP siRNA. Data represent the mean (SD).

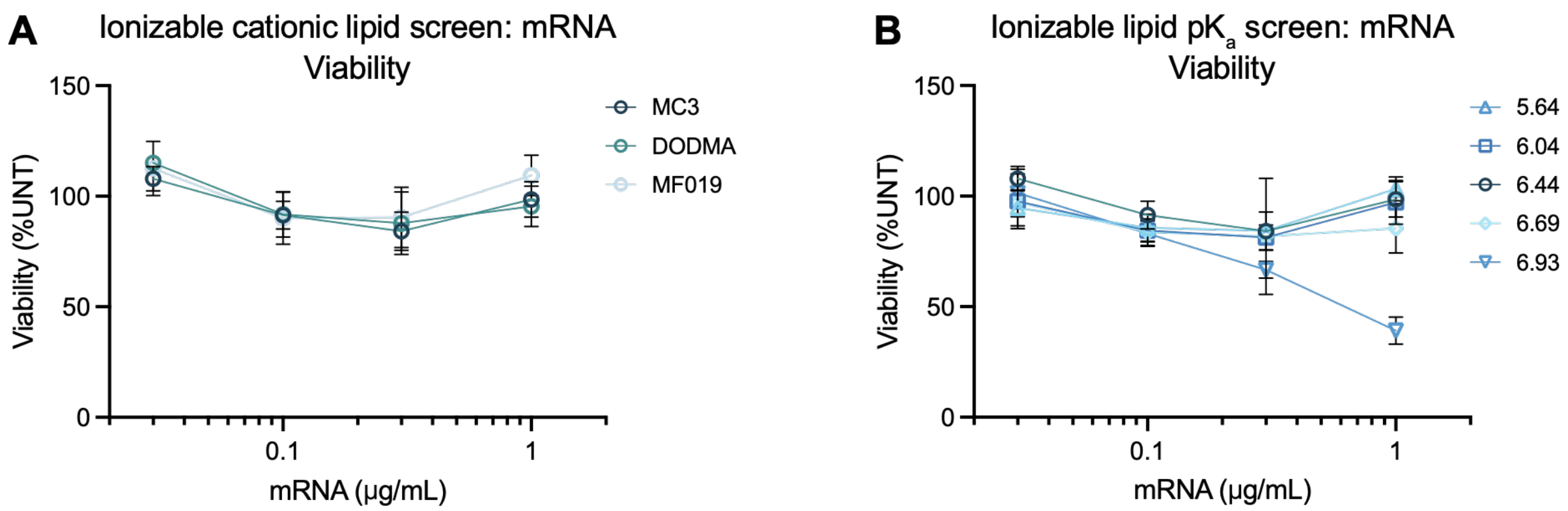

Figure S5. mRNA-containing LNPs prepared with distinct ionizable cationic lipid species differentially affect the viability of primary neurons. (A) Neuronal viability was not impacted by ionizable cationic lipid species (2-way ANOVA, Composition: $F_{(2,36)} = 1.108$, p=0.3413), but worsened with increased mRNA dose (2-way ANOVA, Dose: $F_{(3,36)} = 14.88$, $p<0.0001$). (B) Neuronal viability was affected by both ionizable cationic lipid apparent pK_a (2-way ANOVA, pK_a: $F_{(4,60)} = 14.65$, $p<0.0001$) and mRNA dose (2-way ANOVA, pK_a: $F_{(3,60)} = 17.39$, $p<0.0001$). N=4 biological replicates (N=1 technical replicate) per condition. mRNA treatments were performed at 0.03-1.0 µg/mL FLuc mRNA. Data represent the mean (SD).

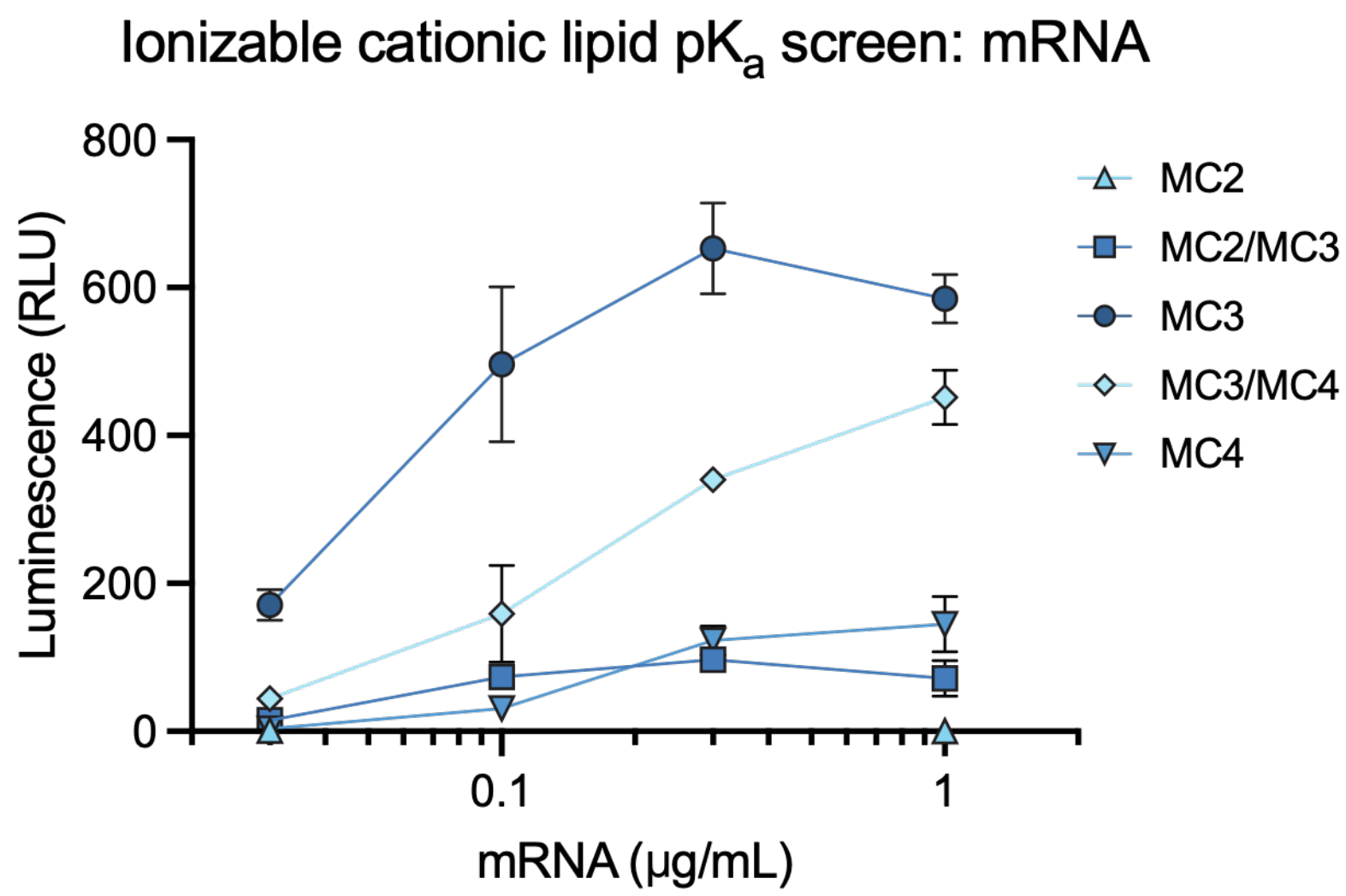

Figure S6. Ionizable cationic lipid apparent pK$_a$ variably impacts the potency of mRNA-containing LNPs in primary neurons. Ionizable cationic lipid apparent pK$_a$ variably impacts the efficacy of LNP-mediated mRNA delivery to primary neurons. LNPs prepared using ionizable cationic lipids with varied apparent pK$_a$s (MC2 = 5.64; MC2/MC3 = 6.03; MC3 = 6.44; MC3/MC4 = 6.69; MC4 = 6.93) and containing FLuc mRNA induced luciferase expression in primary neurons with pK$_a$-dependent efficacy (1-way ANOVA with Dunnett post-test, MC2, MC2/MC3, MC3/MC4, and MC4 vs. MC3 $p<0.0001$). N=4 biological replicates (N=1 technical replicate) per condition. mRNA treatments were performed at 0.03-1.0 µg/mL FLuc mRNA. Data represent the mean (SD).

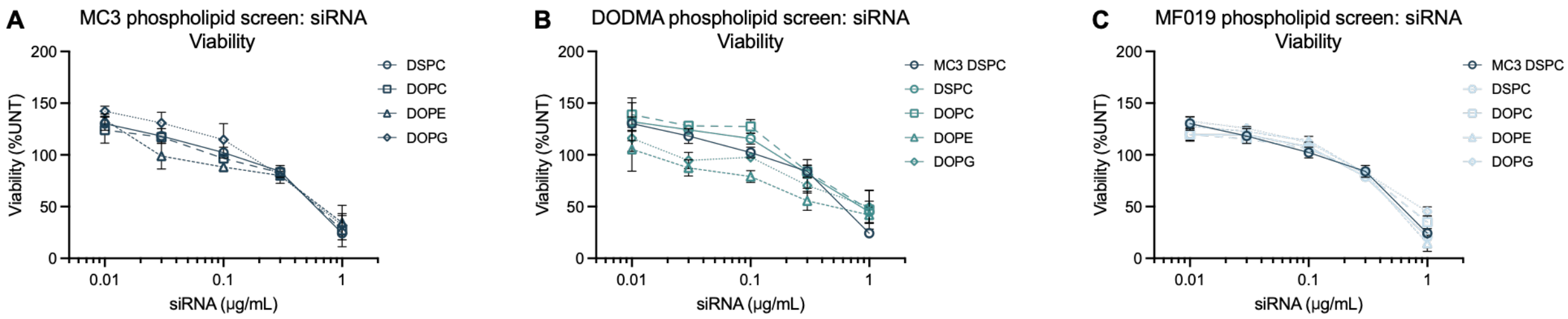

Figure S7. siRNA-containing LNPs prepared with distinct phospholipid species differentially affect primary neuron viability. (A) Neuronal viability was affected by phospholipid species (2-way ANOVA, Phospholipid: $F_{(3,60)}$ = 7.639, p=0.0002) and GFP siRNA dose (2-way ANOVA, Dose: $F_{(4,60)}$ = 311.2, p<0.0001) in the MC3 phospholipid screen. (B) Neuronal viability was affected by phospholipid species (2-way ANOVA, Phospholipid: $F_{(3,60)}$ = 30.48, p<0.0001) and GFP siRNA dose (2-way ANOVA, Dose: $F_{(4,60)}$ = 119.8, p<0.0001) in the DODMA phospholipid screen. (C) Neuronal viability was affected by phospholipid species (2-way ANOVA, Phospholipid: $F_{(3,60)}$ = 22.58, p<0.0001) and GFP siRNA dose (2-way ANOVA, Dose: $F_{(4,60)}$ = 1422, p<0.0001) in the MF019 phospholipid screen. For all viability assays, N=4 biological replicates (N=1 technical replicate) per condition. siRNA treatments were performed at 0.01-1.0 µg/mL GFP siRNA. Data represent the mean (SD).

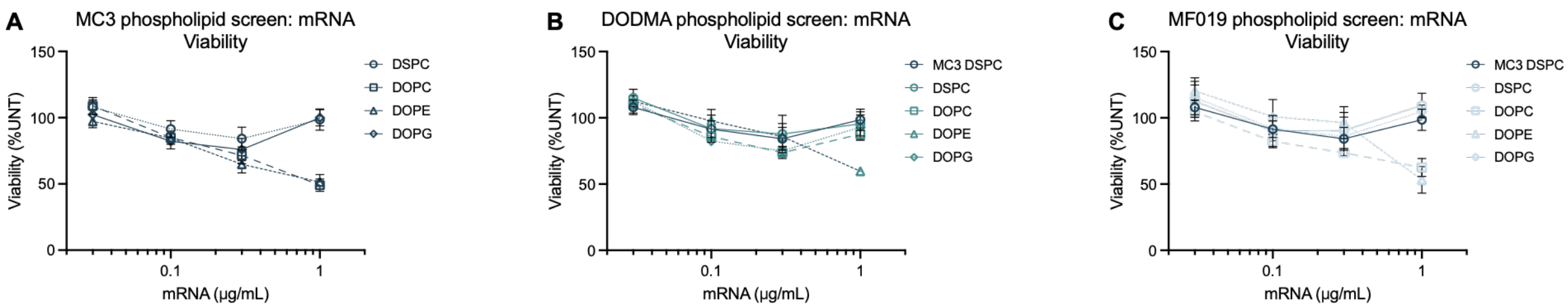

Figure S8. mRNA-containing LNPs prepared with distinct phospholipid species differentially affect primary neuron viability. (A) In the MC3 phospholipid screen, neuronal viability was affected by phospholipid species (2-way ANOVA, Phospholipid: $F_{(3,48)}$ = 36.72, p<0.0001) and mRNA dose (2-way ANOVA, Dose: $F_{(3,48)}$ = 74.97, p<0.0001). (B) In the DODMA phospholipid screen, neuronal viability was affected by phospholipid species (2-way ANOVA, Phospholipid: $F_{(3,48)}$ = 5.042, p=0.0041) and FLuc mRNA dose (2-way ANOVA, Dose: $F_{(3,48)}$ = 64.65, p<0.0001). (C) In the MF019 phospholipid screen, neuronal viability was affected by phospholipid species (2-way ANOVA, Phospholipid: $F_{(3,47)}$ = 12.75, p<0.0001) and FLuc mRNA dose (2-way ANOVA, Dose: $F_{(3,47)}$ = 28.51, p<0.0001). N=4 biological replicates (N=1 technical replicate) per condition. mRNA treatments were performed at 0.03-1.0 µg/mL FLuc mRNA. Data represent the mean (SD).

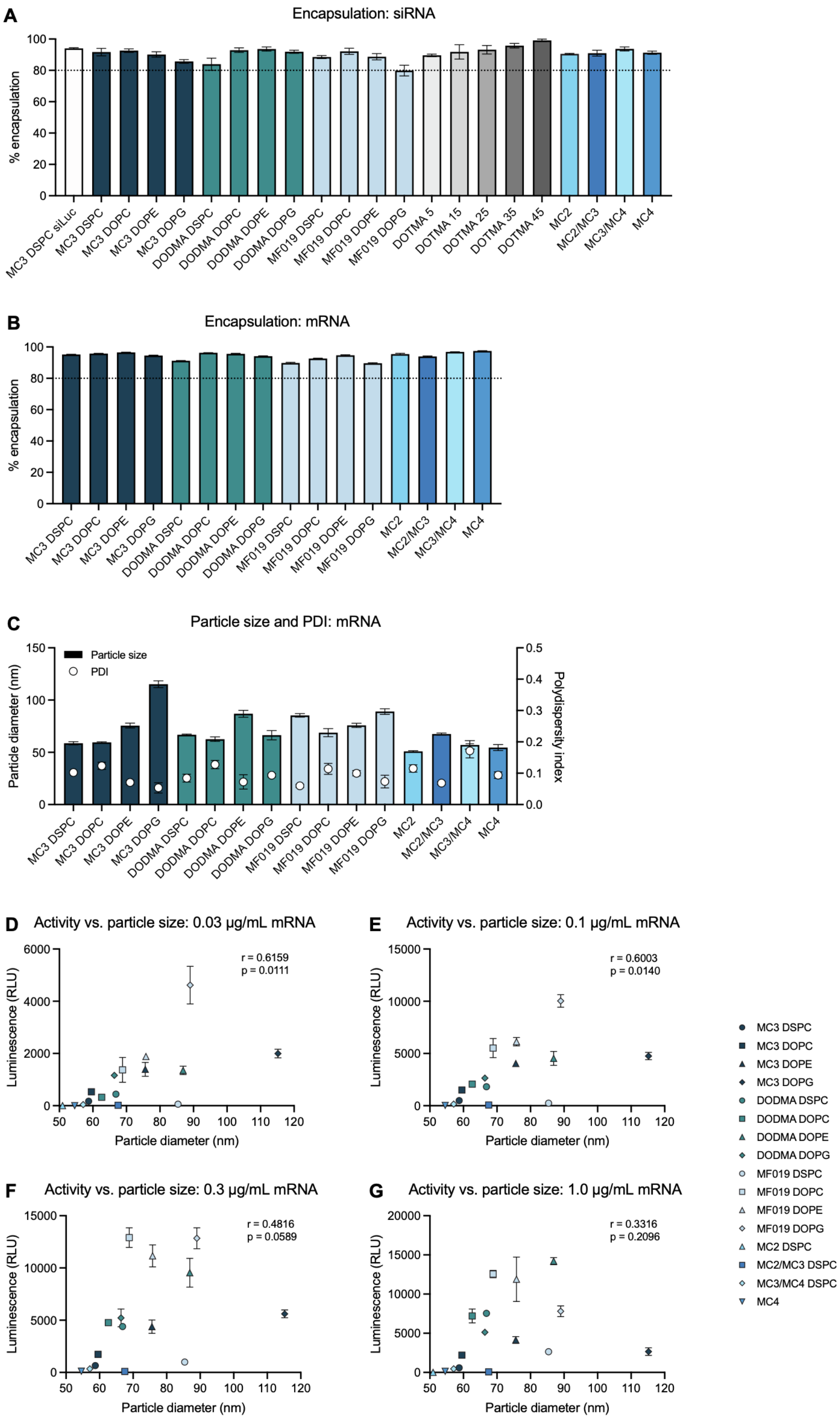
A
Encapsulation: siRNA
% encapsulation
MC3 DSPC siLuc
MC3 DSPC
MC3 DOPC
MC3 DOPE
MC3 DOPG
DODMA DSPC
DODMA DOPC
DODMA DOPE
DODMA DOPG
MF019 DSPC
MF019 DOPC
MF019 DOPE
MF019 DOPG
DOTMA 5
DOTMA 15
DOTMA 25
DOTMA 35
DOTMA 45
MC2
MC2/MC3
MC3/MC4
MC4
B
Encapsulation: mRNA
% encapsulation
C
Particle size and PDI: mRNA
Particle size
PDI
Particle diameter (nm)
Polydispersity index
D
Activity vs. particle size: 0.03 μg/mL mRNA
r = 0.6159
p = 0.0111
E
Activity vs. particle size: 0.1 μg/mL mRNA
r = 0.6003
p = 0.0140
F
Activity vs. particle size: 0.3 μg/mL mRNA
r = 0.4816
p = 0.0589
G
Activity vs. particle size: 1.0 μg/mL mRNA
r = 0.3316
p = 0.2096
Luminescence (RLU)
Particle diameter (nm)
MC3 DSPC
MC3 DOPC
MC3 DOPE
MC3 DOPG
DODMA DSPC
DODMA DOPC
DODMA DOPE
DODMA DOPG
MF019 DSPC
MF019 DOPC
MF019 DOPE
MF019 DOPG
MC2 DSPC
MC2/MC3 DSPC
MC3/MC4 DSPC
MC4

Figure S9. LNP biophysical characteristics are not associated with the activity of GFP siRNA- or FLuc mRNA-containing formulations in *ex vivo* primary neurons. (A) RNA entrapment for all GFP siRNA formulations and (B) FLuc mRNA formulations screened in primary neurons was highly efficient (≥80%) for all formulations. (C) Number-weighted particle diameters and polydispersity indices for all FLuc mRNA formulations. (D-G) In FLuc LNP-treated primary neurons, the relationship between LNP activity and particle diameter depends on FLuc mRNA dose. Particle diameter is positively correlated with activity at 0.03 and 0.1 µg/mL mRNA (Pearson correlation: 0.03 µg/mL r=0.6159 and p=0.0111; 0.1 µg/mL r=0.6003 and p=0.0140), while no correlation was observed at 0.3 µg/mL and 1.0 µg/mL mRNA (Pearson correlation: 0.3 µg/mL r=0.4816 and p=0.0589; 1.0 µg/mL r=0.3316 and p=0.2096). N=3 technical replicates for all FLuc entrapment, particle diameter and PDI measurements. N=4 biological replicates (N=1 technical replicate) for all luminescence measurements. mRNA treatments were performed at 0.03-1.0 µg/mL FLuc mRNA. Data represent the mean (SD).

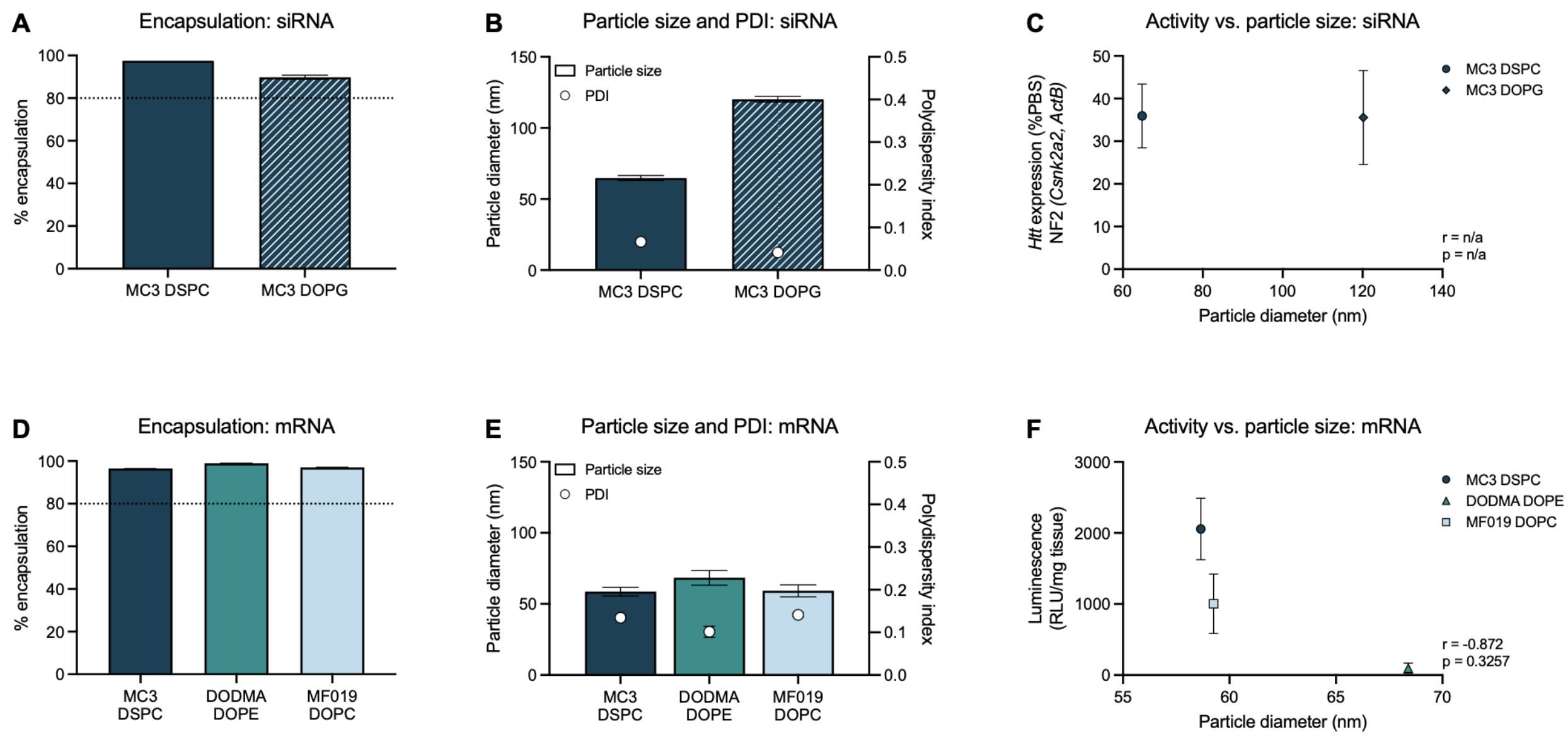

Figure S10. Biophysical characteristics of optimized and benchmark Htt siRNA and FLuc mRNA LNPs screened *in vivo* in striatum. (A) Htt siRNA entrapment was highly efficient (≥80%). (B) Number-weighted particle diameters and polydispersity indices for Htt siRNA formulations. (C) Marked differences in particle diameter between MC3 DSPC and MC3 DOPG LNPs did not impact *in vivo* potency in striatum. 1250 ng of Htt siRNA was delivered per 0.5 µL injection. Brains were collected for analysis seven days post-treatment. (D) FLuc mRNA entrapment was highly efficient (≥80%). (E) Number-weighted particle diameters and polydispersity indices for FLuc mRNA formulations. (F) FLuc mRNA particle diameter is not correlated with *in vivo* potency in striatum (Pearson correlation, r=-0.872 and p=0.3257). 500 ng of FLuc mRNA was delivered per 0.5 µL injection. Brains were collected for analysis four hours post-treatment. N=3 technical replicates for all entrapment, particle diameter and PDI measurements. For activity, N=3-4 biological replicates (for Htt siRNA, N=2 technical replicates; for FLuc mRNA, N=1 technical replicate) per condition. Data represent the mean (SD).

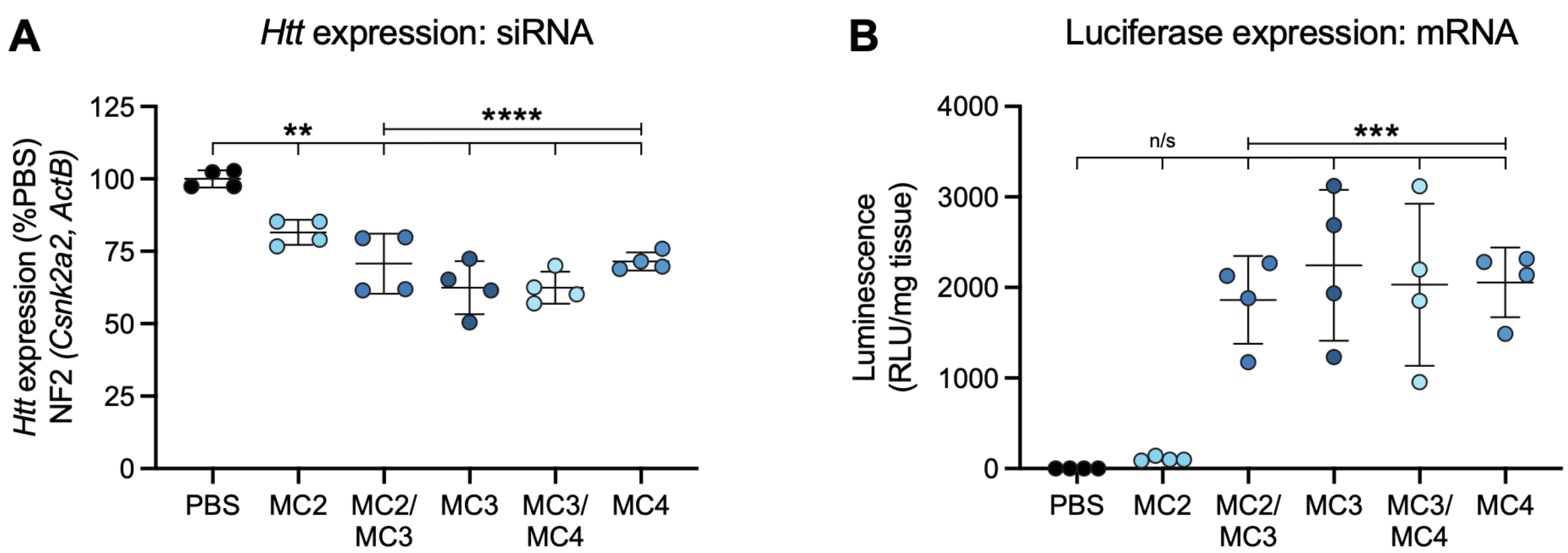

Figure S11. pK_a-variant siRNA- and mRNA-containing LNPs are differentially potent *in vivo* in striatum compared to a PBS-injected control group. LNPs were prepared using ionizable cationic lipids with varied apparent pK_as (MC2 = 5.64; MC2/MC3 = 6.03; MC3 = 6.44; MC3/MC4 = 6.69; MC4 = 6.93. (A) Compared to the PBS-injected control group, pK_a-variant LNPs containing Htt siRNA knocked down striatal *Htt* with variable efficacy (1-way ANOVA with Dunnett post-test, PBS vs. MC2 p=0.0039; PBS vs. MC2/MC3, MC3, MC3/MC4, and MC4 p<0.0001). *Htt* expression in the striata of treated mice is normalized to the level of *Htt* expression measured in the striata of PBS-injected control mice. 1250 ng of Htt siRNA was delivered per 0.5 µL injection. Brains were collected for analysis seven days post-treatment. (B) Compared to the PBS-injected control group, FLuc mRNA-containing LNPs prepared with MC2/MC3, MC3, MC3/MC4, and MC4 induced striatal luciferase expression (1-way ANOVA with Dunnett post-test, PBS vs. MC2/MC3 p=0.0008; PBS vs. MC3 p=0.0001; PBS vs. MC3/MC3 p=0.0003; PBS vs. MC4 p=0.0003), while the MC2 formulation did not (1-way ANOVA with Dunnett post-test, PBS vs. MC2 p=0.9989). Luminescence in the striata of treated mice is shown relative to the background signal measured in the striata of PBS-injected control mice and normalized to RLU/mg tissue. 500 ng of FLuc mRNA was delivered per 0.5 µL injection. Brains were collected for analysis at four hours post-treatment. For quantitative RT-PCR, N=4 biological replicates (N=2

technical replicates) per condition. For luciferase assays, N=4 biological replicates (N=1 technical replicate) per condition. Data represent the mean (SD).

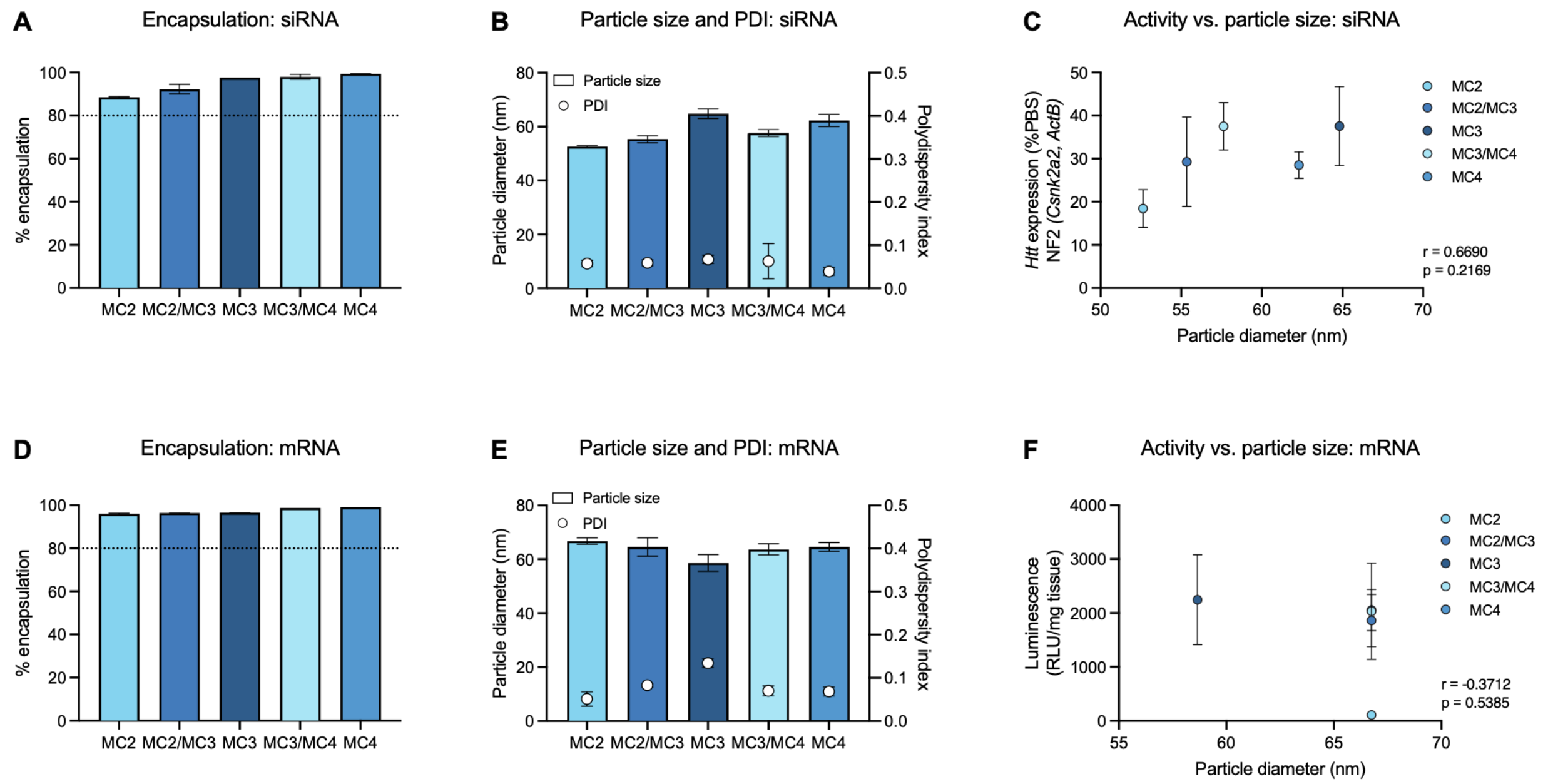

Figure S12. Biophysical characteristics of pK_a variant siRNA and mRNA LNPs screened in striatum *in vivo*. (A) pK_a variant Htt siRNA entrapment was highly efficient (≥80%). (B) Number-weighted particle diameters and polydispersity indices for pK_a variant Htt siRNA formulations. (C) pK_a variant Htt siRNA particle diameter is not correlated with *in vivo* potency in striatum (Pearson correlation, r=0.6690 and p=0.2169). *Htt* expression in the striata of treated mice is normalized to the level of *Htt* expression measured in the striata of PBS-injected control mice. 1250 ng of Htt siRNA was delivered per 0.5 µL injection. Brains were collected for analysis seven days post-treatment. (D) pK_a-variant FLuc mRNA entrapment was highly efficient (≥80%). (E) Number-weighted particle diameters and polydispersity indices for pK_a variant FLuc mRNA formulations. (F) pK_a variant FLuc mRNA particle diameter is not correlated with *in vivo* potency in striatum (Pearson correlation, r=-0.3712 and p=0.5385). Luminescence in the striata of treated mice is shown relative to the background signal measured in the striata of PBS-injected control mice and normalized to RLU/mg tissue. 500 ng of FLuc mRNA was delivered per 0.5 µL injection. Brains were collected for analysis at four hours post-treatment. N=3 technical replicates for all

entrapment, particle diameter and PDI measurements. For activity, N=4 biological replicates (for Htt siRNA, N=2 technical replicates; for FLuc mRNA, N=1 technical replicate) per condition. Data represent the mean (SD).

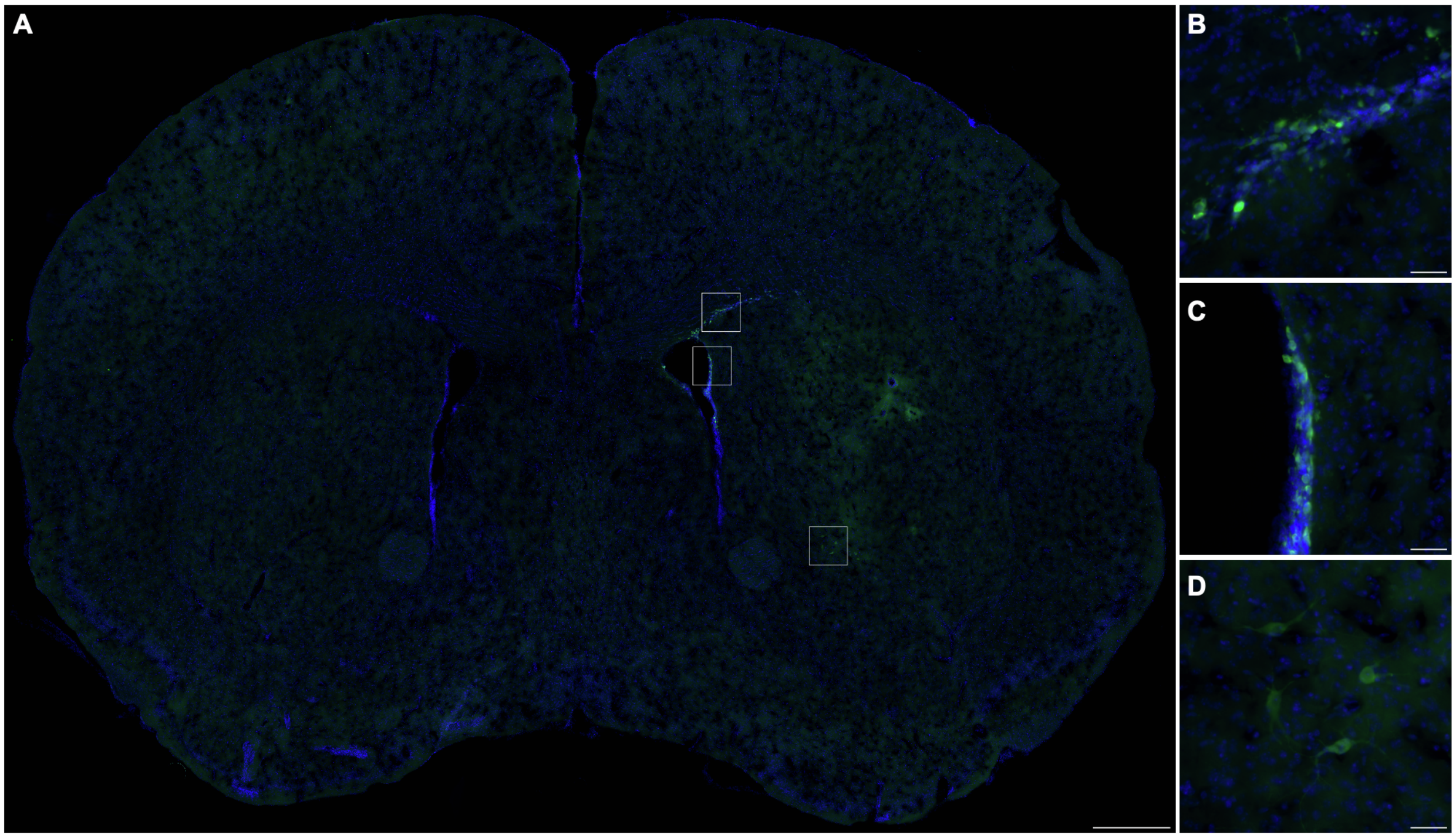

Figure S13. Direct intrastriatal LNP delivery results in luciferase expression in cells throughout and proximal to the striatum. (A) Luciferase expression is detectable in the brain following direct injection of MC3 DSPC LNPs containing FLuc mRNA. Luciferase-expressing cells are present in the corpus callosum (B), adjacent to the lateral ventricle (C) and throughout the striatum (D). Cell nuclei are shown in blue and luciferase-expressing cells are shown in green. 500 ng of FLuc mRNA was delivered per 0.5 µL injection. Brains were collected for analysis four hours post-treatment. The insets outlined in A are presented in the same vertical order in panels B-D. In A, scale bar = 500 µm; in B-D, scale bar = 50 µm.

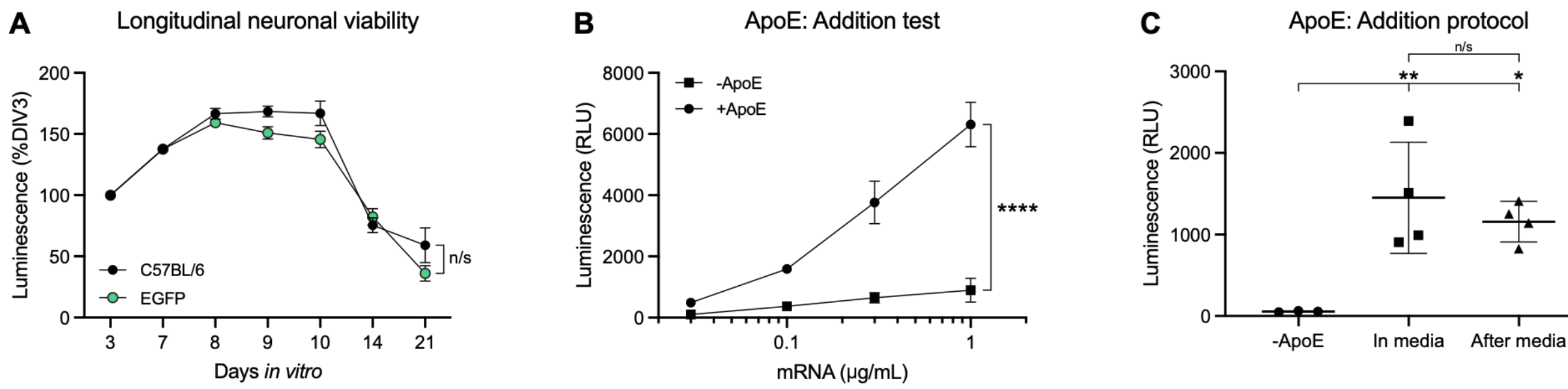

Figure S14. Assay development for LNP treatment of *ex vivo* primary neurons. (A) The viability of primary neurons isolated from C57BL/6 and transgenic *EGFP* E17.5 embryos was measured over 21 days *in vitro* (DIV) to identify appropriate time points for LNP treatment and sample collection. Time significantly impacted neuronal viability (2-way ANOVA, Days *in vitro*: $F_{(2.711,\ 27.11)}$ = 677.4, p<0.0001), while genotype did not (2-way ANOVA, Genotype: $F_{(10,\ 60)}$ = 1.962, p=0.0539). Data are presented as %DIV3. N=6 biological replicates for all measurements. (B) Luciferase expression in C57BL/6 neurons was assayed following treatment with LNPs containing FLuc mRNA in the absence or presence of ApoE to assess the impact of ApoE on neuronal LNP transfection (2-way ANOVA, ApoE: $F_{(1,19)}$ = 29.37, p<0.0001). N=3 biological replicates for all measurements. (C) ApoE was added to primary neuron media before or immediately following LNP dilution to establish an ApoE addition protocol appropriate for neuronal LNP transfection. Primary neurons were treated with LNPs containing FLuc mRNA at 0.1 µg/mL. -ApoE = ApoE not added to media; In media = ApoE added to media before LNP dilution; After media = ApoE added to media following LNP dilution (1-way ANOVA with Tukey post-test, -ApoE vs. In media p=0.0084; -ApoE vs. After media p=0.0284; In media vs. After media p=0.6363). N=3-4 biological replicates for all measurements. Data represent the mean (SD).

Table S1. LNP activity is not significantly correlated with ionizable cationic lipid apparent pK_a. In *ex vivo* primary neurons and *in vivo* in the brain, LNP activity and apparent pK_a are not significantly correlated. Relationships between LNP activity and apparent pK_a were assessed by Pearson correlation for each RNA cargo and dose evaluated in the pK_a variant LNP formulation series (see also Figures 2C, 3B, S4, S6 and S12).

Experimental system (RNA)	Comparison	R	95% confidence interval	p
Ex vivo (*GFP* siRNA)	*GFP* knockdown vs. apparent pK_a	-0.5058	-0.9601 to 0.6779	0.3816
Ex vivo (Luc mRNA)	Luminescence vs. apparent pK_a (0.03 µg/mL mRNA)	0.1884	-0.8322 to 0.9181	0.7616
Ex vivo (Luc mRNA)	Luminescence vs. apparent pK_a (0.1 µg/mL mRNA)	0.2310	-0.8180 to 0.9248	0.7085
Ex vivo (Luc mRNA)	Luminescence vs. apparent pK_a (0.3 µg/mL mRNA)	0.4077	-0.7412 to 0.9487	0.4956
Ex vivo (Luc mRNA)	Luminescence vs. apparent pK_a (1.0 µg/mL mRNA)	0.5158	-0.6725 to 0.9608	0.3737
In vivo (*Htt* siRNA)	*Htt* knockdown vs. apparent pK_a	-0.6632	-0.9750 to 0.5281	0.2224
In vivo (Luc mRNA)	Luminescence vs. apparent pK_a	0.8006	-0.2781 to 0.9862	0.1036

Table S2. Quantitative RT-PCR primer sequences.

Target	Forward (5' to 3')	Reverse (5' to 3')
Csnk2a2	CCACATAGACCTAGATCCACACTTCA	AGGTGCCTGTTCTCACTATGGATAA
ActB	CCAGCCTTCCTTCTTGGGTAT	TGTGTTGGCATAGAGGTCTTTACG
EGFP	CAGAAGAACGGCATCAAGG	ACGAACTCCAGCAGGACCAT
Htt	TGGTAATGACAGTTGAGGCCC	CCAGGTTGTACTGCAATGGCT
